# The epidemiology and burden of ten mental disorders in countries of the Association of Southeast Asian Nations (ASEAN), 1990–2021: findings from the Global Burden of Disease Study 2021

**DOI:** 10.1016/S2468-2667(25)00098-2

**Published:** 2025-05-27

**Authors:** GBD 2021 ASEAN Mental Disorders Collaborators, Anna Szücs, Stephanie C C van der Lubbe, Jorge Arias de la Torre, Jose M Valderas, Simon I Hay, Catherine Bisignano, Brooks W Morgan, Swetha Acharya, Qorinah Estiningtyas Sakilah Adnani, Geminn Louis Carace Apostol, Muhammad Shahzad Aslam, Yuni Asri, Zaw Zaw Aung, Gading Ekapuja Aurizki, Atif Amin Baig, Amiel Nazer C Bermudez, Muthia Cenderadewi, Pojsakorn Danpanichkul, Ferry Efendi, Crystal Amiel M Estrada, Nelsensius Klau Fauk, Ni Kadek Yuni Fridayani, Fernando Barroga Garcia, Faizul Hasan, Umar Idris Ibrahim, Muhana Fawwazy Ilyas, Nahlah Elkudssiah Ismail, Jazlan Jamaluddin, Roland Dominic G Jamora, Jost B Jonas, Kehinde Kazeem Kanmodi, Kashif Ullah Khan, Fireza Husnul Khotimah, Yun Jin Kim, Maria Dyah Kurniasari, Christina Yeni Kustanti, Dian Kusuma, Tri Laksono, Jerrald Lau, Arianna Maever Loreche, Zheng Feei Ma, Joemer C Maravilla, Roy Rillera Marzo, Richard James Maude, Thantrira Porntaveetus, Setyaningrum Rahmawaty, Alina Rodriguez, Bedanta Roy, Afeez Abolarinwa Salami, Yoseph Leonardo Samodra, Chandrashekhar T Sreeramareddy, Vetriselvan Subramaniyan, Thitiporn Sukaew, Desy Sulistiyorini, Lourdes Bernadette C Sumpaico-Tanchanco, Anggi Lukman Wicaksana, Yen Jun Wong, Mustafa Z Younis, Yves Miel H Zuniga, Christopher J L Murray, Damian F Santomauro, Marie Ng

## Abstract

**Background:**

The Association of Southeast Asian Nations (ASEAN), a geopolitical and economic network of ten member states, recognises mental disorders as a health priority; however, sparse epidemiological data hinder the development of effective strategies to reduce their prevalence and burden. We aimed to examine the prevalence, morbidity, and disease burden associated with ten mental disorders from 1990 to 2021 in the ASEAN.

**Methods:**

As part of the Global Burden of Diseases, Injuries, and Risk Factors Study (GBD 2021), we analysed estimates for depressive disorders, anxiety disorders, bipolar disorders, schizophrenia, autism spectrum disorders, conduct disorder, attention-deficit hyperactivity disorder (ADHD), eating disorders, idiopathic developmental intellectual disability, and other mental disorders in ten ASEAN member states (Brunei, Cambodia, Indonesia, Laos, Malaysia, Myanmar, the Philippines, Singapore, Thailand, and Viet Nam). Case definitions were based on Diagnostic and Statistical Manual of Mental Disorders or ICD criteria. Prevalence estimates by age, sex, year, and location were derived using DisMod-MR 2.1, a Bayesian meta-regression modelling tool. Disease burden was quantified by estimating years lived with disability (YLDs), years of life lost (YLLs), and disability-adjusted life-years (DALYs). Estimates are presented with 95% uncertainty intervals (UIs).

**Findings:**

In 2021, 80·4 million (95% UI 73·8–87·2) cases of mental disorders were reported across ASEAN countries, representing a 70·0% (63·5–77·2) increase since 1990. The age-standardised prevalence of mental disorders was 11·9% (10·9–12·9) in 2021, ranging from 10·1% (9·1–11·3) in Viet Nam to 13·2% (11·6–15·3) in Malaysia, with anxiety and depressive disorders being the most common. The age-standardised prevalence of mental disorders increased by 6·5% (3·7–9·8) between 1990 and 2021. Mental disorders accounted for 11·2 million (8·5–14·3) DALYs in 2021, representing an 87·4% (81·1–94·0) increase since 1990. The 10–14 years age group had the highest disease burden attributable to mental disorders, which accounted for 16·3% (12·7–20·5) of total DALYs in this age group. The largest relative increases in the number of cases of mental disorders between 1990 and 2021 were seen in older adults (182·8% [174·9–192·1] among those aged ≥70 years), despite small relative changes in prevalence in these age groups.

**Interpretation:**

The increase in mental disorder prevalence and burden found in this study might partly reflect recent improvements in detection. However, mental disorders now rank among the top ten causes of disease burden in all ASEAN countries except Myanmar, underscoring the urgent need for a comprehensive intersectoral approach to address prevention and treatment gaps across entire populations.

**Funding:**

Gates Foundation.

## Introduction

Mental disorders affect individual health and societal welfare.^1^ In 2021, mental disorders contributed to 17·2% of the total years lived with disability (YLDs) worldwide.^2^, ^3^ To “promote mental health and well-being” is a part of UN Sustainable Development Goal 3.^4^ Recent international efforts, such as the WHO Special Initiative for Mental Health started in 2019,^5^ have striven to increase access to mental health care especially in low-income and middle-income countries, where essential mental health infrastructure is lacking.^5^ However, even in high-income countries with well implemented mental health-care systems, substantial gaps in diagnosis and treatment remain.^6^

As the world's fifth-largest economy,^7^ and with a population 1·5 times larger than that of the EU,^8^ the Association of Southeast Asian Nations (ASEAN) is an economic and geopolitical network comprising ten countries of diverse historical, political, cultural, and religious backgrounds: Brunei, Cambodia, Indonesia, Laos, Malaysia, Myanmar, the Philippines, Singapore, Thailand, and Viet Nam. The ASEAN is home to 672 million people, close to 9% of the world population.^9^ It encompasses countries with varying socioeconomic levels: lower-middle-income nations, namely Cambodia, Myanmar, and Laos, ranked 148th, 144th, and 139th, respectively, out of 192 countries on the UN Human Development Index (HDI);^10^ middle-income countries, namely the Philippines (113th), Indonesia (112th), and Viet Nam (107th); and upper-middle to high-income countries, namely Thailand (66th), Malaysia (63rd), Brunei (55th), and Singapore (9th). Despite these differences, the promotion of mental health is a key health priority for the region as per the ASEAN Post-2015 Health Development Agenda (health priority 5 in the 5-year goals for 2021–25).^11^

**Research in context**

**Evidence before this study**

We searched PubMed and Google Scholar for studies published from database inception until Oct 21, 2024, on mental disorders in the Association of Southeast Asian Nations (ASEAN) or any of its member states using the search terms “mental disorders”, “anxiety”, “depress*”, “bipolar”, “schizophren*”, “behav* disorder”, “autis*”, “attention-deficit hyperactivity disorder”, “ADHD”, “eating disorders”, and “idiopathic developmental intellectual disability” separated with “AND” from the terms “ASEAN”, “Brunei”, “Darussalam”, “Myanmar”, “Cambodia”, “Indonesia”, “People's Democratic Republic of Lao”, “Lao PDR”, “Malaysia”, “Philippines”, “Singapore”, “Thailand”, and “Viet Nam”. Searches revealed little but increasing research on the prevalence and burden of mental disorders in the ASEAN region. Country-specific studies within ASEAN varied in terms of study design and assessment measures used, leading to heterogeneous findings. Across all studies, anxiety disorders had the highest prevalence, ranging from approximately 5% to 20%, followed by depressive disorders, with prevalence estimates between 2% and 15%. No study to date has analysed the burden of disease associated with mental disorders in the ASEAN.

**Added value of this study**

This study offers a systematic overview of the prevalence and burden of mental disorders by age and sex across all ASEAN member states. Our results show geographical, temporal, sex-specific, and age-specific variations in the epidemiology of mental disorders. The findings in this study concerningly show a high burden of mental disorders in younger populations and a large relative increase in the number of cases in older populations. By highlighting these issues, this work provides quantitative evidence in support of policy reform.

**Implications of all the available evidence**

With the next ASEAN Post-2015 Health Development Agenda 5-year plan for 2026–30 in sight, our findings call for greater investment in addressing the growing public health challenge of mental disorders. Since mental disorders at the population level are primarily driven by social, environmental, and structural factors, the ASEAN must leverage its geopolitical influence to facilitate multisectoral and intergovernmental policy changes to overcome current barriers to mental health care. These changes include context-appropriate mental health promotion targeting culturally anchored stigma and misconceptions, in addition to measures ensuring equitable access to mental health care within the community.

National strategies for the development of mental health care vary considerably within the ASEAN.^12^, ^13^ Lower-middle-income nations tend to have little mental health infrastructure, and it has been disrupted by recent armed conflicts and natural catastrophes.^14^, ^15^, ^16^ While middle-income countries have started investing in mental health care over the past two decades,^17^, ^18^ underdiagnosis and undertreatment remain common.^19^, ^20^ Upper-middle-income and high-income countries possess mental health infrastructures, yet struggle with other barriers to mental health care, such as suboptimal levels of public knowledge and awareness,^21^, ^22^ sociocultural stigma,^23^ and insufficient insurance coverage and access to services.^24^ These disparities became particularly evident during the COVID-19 pandemic.^25^

Understanding the current state and trajectories of mental disorders in the ASEAN is crucial to devising appropriate health interventions and policies. However, a comprehensive analysis of the prevalence and disease burden of mental disorders in the region is needed. To address this gap, we leveraged the Global Burden of Diseases, Injuries, and Risk Factors Study (GBD) 2021 to examine the prevalence, mortality, and morbidity of ten mental disorders by age and sex from 1990 to 2021 in the ten ASEAN countries to provide epidemiological insights into the landscape of mental disorders in the region. This Article was produced as part of the GBD Collaborator Network and in accordance with the GBD Protocol.^26^

## Methods

### Overview

GBD 2021 systematically evaluated 371 diseases and injuries, along with 88 risk factors, across 204 countries and territories.^3^, ^27^ The study estimates various metrics, such as incidence, prevalence, cause-specific deaths, years of life lost (YLLs; representing fatal health burden), YLDs (representing non-fatal health burden), and disability-adjusted life-years (DALYs; the sum of YLLs and YLDs), synthesising data from a large number of sources, including national surveys, disease registries, and published literature (appendix 1 pp 4–6). These metrics are broken down by age and sex for each country from 1990 to 2021. Further details about the analytical strategy of GBD 2021 can be found in previous publications^3^, ^27^ and in appendix 1 (pp 4–13).

This Article complies with GATHER recommendations (appendix 1 p 46).^28^ Data visualisations were done using Python version 3.11.9.

### Case definitions

The mental disorders included in GBD 2021 are depressive disorders (major depressive disorder and dysthymia), anxiety disorders (a combined estimate of all subtypes), bipolar disorders (a combined estimate of all subtypes), schizophrenia, autism spectrum disorders, conduct disorder, attention-deficit hyperactivity disorder (ADHD), eating disorders (anorexia nervosa and bulimia nervosa), idiopathic developmental intellectual disability, and a category for other mental disorders (an aggregate group of personality disorders without other comorbid mental disorders). To ensure comparability in measurement, cases were defined using criteria from the Diagnostic and Statistical Manual of Mental Disorders (DSM) and the ICD. Details for the exact codes used for each cause are summarised in appendix 1 (p 13) and in the GBD online method appendices.^29^

### Data sources

Data were gathered from multinational and national surveys, vital registration systems (for causes of death), and published literature consisting of both scientific publications and grey literature identified through systematic searches. Sources were required to be representative of the general population, and the definitions of causes in the data had to align with the case definitions described above. Data on prevalence, incidence, or mortality were extracted by age and sex whenever possible. Relevant data were identified for all ASEAN countries except Brunei and Cambodia, with temporal coverage varying across countries. Details of the included data sources and data selection considerations can be found in appendix 1 (pp 5–6, 14).

### Prevalence

Estimating the prevalence of mental disorders involved a two-step process. First, epidemiological estimates from various studies identified through systematic reviews were evaluated for potential biases stemming from differences in diagnostic tools (eg, diagnostic interviews versus symptom scales), recall periods (point versus past-year prevalence), and types of interviewer (health professionals versus lay interviewers). Adjustments to address these biases included using pooled differences in the logit-transformed prevalence of gold-standard and alternative estimates, and conducting network meta-analyses to ascertain the magnitude of deviations and derive suitable correction factors.^30^ Second, the bias-corrected data were analysed using DisMod-MR 2.1, a Bayesian meta-regression epidemiological modelling tool.^3^ Specific adjustment was made to account for the effect of COVID-19. Further details of the estimation process are available in appendix 1 (pp 14–15) and in previous publications.^3^, ^30^, ^31^

### Severity proportions

Severity proportions were calculated to reflect the different levels of disability (or sequelae) associated with a specific disorder, such as mild, moderate, and severe presentations. For conduct disorder, ADHD, autism spectrum disorders, bipolar disorders, and schizophrenia, severity distributions were derived from various epidemiological data, expert feedback, systematic reviews, and meta-analyses. For example, to obtain the severity proportion for schizophrenia, data from a systematic review were analysed using meta-regression to derive pooled estimates of the overall proportion of acute and residual states of schizophrenia. Severity proportions were applied to the total prevalent cases, and prevalence estimates for each level of severity were calculated using DisMod-MR 2.1. Further details on severity proportions are available in previous publications.^3^, ^29^, ^30^

### Mortality and years of life lost

Mortality was estimated for eating disorders, specifically anorexia nervosa, using the GBD standard Cause of Death Ensemble model (CODEm), which attributes the unique, most direct cause to each death. Mortality resulting from indirect causes was not attributed or accounted for.^30^, ^31^ YLLs were calculated by multiplying the estimated number of deaths due to the specific cause by the standard life expectancy at the corresponding age. This metric provides quantification of premature mortality. The CODEm model and relevant metrics are detailed in our previous publication.^27^

### Years lived with disability

YLDs were calculated by multiplying the severity-specific prevalence by their respective disability weights. Disability weight represents the relative health loss due to a particular health state. It is expressed on a scale from 0 (no health loss) to 1 (equivalent to death). Disability weight estimates were obtained from a global community-based survey and other online surveys conducted in multiple countries.^32^ Because independence was assumed when estimating the burden attributed to each cause, to account for the co-occurrence of diseases and more accurately reflect the differences in disability experienced by individuals with multiple sequelae, a simulation was conducted to determine comorbidity correction factors. The factors were subsequently used to adjust the final YLD estimates. YLDs reflect the non-fatal effect of diseases and injuries and provide quantification of the magnitude of health loss associated with each outcome. Details of the calculations can be found in previous publications.^3^, ^30^

Disability-adjusted life-years (DALYs) were calculated by summing YLLs and YLDs according to location, year, age, sex, and cause, reflecting both fatal and non-fatal health burden. For mental disorders not recognised as causes of death (ie, all except for anorexia nervosa), YLLs were not estimated, and YLDs were equal to DALYs. Age-standardised rates per 100 000 people were estimated using the GBD world population age standard. Changes in prevalence and burden over time were assessed by comparing shifts in age-standardised rates and total numbers. YLDs, YLLs, and DALYs were estimated by sex, across 20 age groups (from birth to age ≥95 years in 5-year intervals), and for every year from 1990 to 2021. For all estimates, 95% uncertainty intervals (UIs) were derived using the 2·5th and 97·5th percentiles from 500 draws of the posterior distribution generated by Markov chain Monte Carlo sampling.

### Role of the funding source

The funders of the study had no role in study design, data collection, data analysis, data interpretation, or writing of the report.

## Results

In 2021, there were an estimated 80·4 million (95% UI 73·8–87·2) cases of mental disorders in the ASEAN (table). The highest numbers of cases were observed in Indonesia (32·9 million [30·3–35·8] cases), the Philippines (14·0 million [12·9–15·1]), and Viet Nam (10·1 million [9·09–11·3]), proportional to the countries’ overall population sizes (appendix 1 p 16). Compared with 1990, the total number of cases of mental disorders in the ASEAN increased by 70·0% (63·5–77·2; table).

**Table:** Total and sex-disaggregated number of prevalent cases and age-standardised prevalence of mental disorders in 1990 and 2021, and percentage change from 1990 to 2021, in the ASEAN and each of its member countries

		**1990**		**2021**		**Percentage change, 1990–2021**	
		Prevalent cases, millions	Age-standardised prevalence, %	Prevalent cases, millions	Age-standardised prevalence, %	Number of prevalent cases	Age-standardised prevalence
ASEAN		47·3 (42·9 to 52·3)	11·2% (10·2 to 12·3)	80·4 (73·8 to 87·2)	11·9% (10·9 to 12·9)	70·0% (63·5 to 77·2)	6·5% (3·7 to 9·8)
	Male	22·6 (20·4 to 25·0)	10·7% (9·8 to 11·7)	37·6 (34·5 to 40·8)	11·3% (10·4 to 12·3)	66·2% (60·1 to 72·7)	5·5% (2·7 to 8·3)
	Female	24·6 (22·3 to 27·3)	11·6% (10·6 to 12·7)	42·7 (39·1 to 46·8)	12·5% (11·4 to 13·7)	73·4% (66·5 to 81·8)	7·5% (4·2 to 11·2)
Brunei		0·0265 (0·0242 to 0·0292)	11·0% (10·1 to 11·9)	0·0512 (0·0454 to 0·0578)	11·7% (10·5 to 13·1)	93·3% (79·7 to 110·1)	6·9% (−0·2 to 15·5)
	Male	0·0146 (0·0133 to 0·0161)	11·3% (10·4 to 12·3)	0·0270 (0·0243 to 0·0301)	12·0% (10·8 to 13·4)	85·4% (74·4 to 98·6)	6·2% (0·4 to 13·1)
	Female	0·0119 (0·0108 to 0·0133)	10·6% (9·7 to 11·7)	0·0242 (0·0211 to 0·0279)	11·4% (10·0 to 13·2)	102·9% (83·7 to 125·6)	7·5% (−1·8 to 18·8)
Cambodia		1·12 (0·993 to 1·27)	12·4% (11·2 to 13·8)	2·12 (1·86 to 2·44)	12·9% (11·3 to 14·8)	89·0% (70·7 to 113·1)	3·8% (−5·8 to 16·6)
	Male	0·498 (0·435 to 0·561)	11·5% (10·4 to 12·8)	0·968 (0·854 to 1·09)	12·1% (10·7 to 13·6)	94·2% (76·6 to 117·2)	4·5% (−3·9 to 14·8)
	Female	0·626 (0·552 to 0·711)	13·0% (11·7 to 14·6)	1·16 (1·00 to 1·35)	13·6% (11·8 to 15·8)	84·8% (64·3 to 112·5)	4·0% (−6·9 to 18·4)
Indonesia		19·0 (17·3 to 20·8)	10·8% (9·9 to 11·8)	32·9 (30·3 to 35·8)	11·8% (10·9 to 12·8)	73·8% (67·2 to 82·1)	9·0% (5·7 to 13·0)
	Male	9·02 (8·12 to 9·90)	10·2% (9·3 to 11·1)	15·4 (14·2 to 16·8)	11·1% (10·2 to 12·1)	71·2% (65·0 to 78·5)	8·2% (5·2 to 11·9)
	Female	9·94 (9·02 to 10·9)	11·4% (10·4 to 12·4)	17·5 (16·1 to 19·2)	12·5% (11·5 to 13·6)	76·2% (68·6 to 85·0)	9·8% (6·0 to 14·7)
Laos		0·465 (0·414 to 0·525)	12·5% (11·3 to 13·9)	0·901 (0·782 to 1·04)	12·8% (11·1 to 14·8)	93·8% (73·2 to 116·8)	2·6% (−7·9 to 14·9)
	Male	0·221 (0·196 to 0·249)	12·1% (10·8 to 13·4)	0·439 (0·387 to 0·501)	12·5% (11·1 to 14·2)	98·6% (79·9 to 119·6)	3·9% (−5·5 to 13·7)
	Female	0·244 (0·216 to 0·276)	12·9% (11·6 to 14·5)	0·462 (0·391 to 0·547)	13·1% (11·1 to 15·4)	89·4% (66·2 to 115·2)	1·7% (−10·6 to 15·8)
Malaysia		1·85 (1·66 to 2·06)	11·7% (10·6 to 12·9)	4·13 (3·63 to 4·78)	13·2% (11·6 to 15·3)	122·8% (100·0 to 148·2)	12·6% (1·7 to 25·1)
	Male	0·870 (0·784 to 0·962)	10·9% (9·9 to 12·0)	1·95 (1·72 to 2·23)	12·3% (10·8 to 14·0)	124·7% (105·8 to 147·3)	12·5% (2·7 to 23·3)
	Female	0·983 (0·873 to 1·11)	12·5% (11·2 to 13·9)	2·17 (1·87 to 2·58)	14·1% (12·2 to 16·8)	121·1% (93·6 to 150·9)	13·3% (−0·3 to 28·1)
Myanmar		4·44 (3·94 to 5·01)	11·5% (10·3 to 12·8)	6·64 (5·72 to 7·63)	11·9% (10·3 to 13·6)	49·5% (35·0 to 67·2)	4·0% (−6·5 to 15·3)
	Male	2·12 (1·89 to 2·39)	11·0% (9·9 to 12·3)	3·00 (2·67 to 3·37)	11·3% (10·1 to 12·7)	41·2% (28·9 to 55·2)	2·6% (−5·7 to 12·4)
	Female	2·32 (2·04 to 2·61)	11·9% (10·5 to 13·3)	3·64 (3·11 to 4·25)	12·4% (10·6 to 14·5)	57·0% (39·4 to 77·7)	4·8% (−7·3 to 18·1)
Philippines		6·77 (6·15 to 7·41)	12·0% (11·0 to 13·0)	14·0 (12·9 to 15·1)	12·9% (11·9 to 13·9)	106·3% (100·6 to 113·1)	7·5% (5·2 to 10·3)
	Male	3·31 (2·99 to 3·62)	11·5% (10·5 to 12·5)	6·63 (6·10 to 7·17)	12·1% (11·2 to 13·1)	100·3% (95·1 to 106·2)	5·4% (3·4 to 7·9)
	Female	3·46 (3·15 to 3·80)	12·4% (11·4 to 13·4)	7·34 (6·76 to 7·96)	13·6% (12·6 to 14·6)	112·1% (105·7 to 119·6)	9·4% (6·7 to 12·6)
Singapore		0·372 (0·343 to 0·405)	12·3% (11·4 to 13·4)	0·653 (0·586 to 0·730)	12·3% (11·0 to 13·8)	75·6% (62·3 to 89·4)	−0·6% (−7·9 to 6·2)
	Male	0·185 (0·170 to 0·204)	12·5% (11·4 to 13·6)	0·333 (0·302 to 0·368)	12·8% (11·6 to 14·3)	79·4% (68·2 to 91·5)	2·7% (−3·4 to 8·2)
	Female	0·187 (0·170 to 0·205)	12·1% (11·2 to 13·3)	0·321 (0·283 to 0·367)	11·7% (10·3 to 13·3)	71·8% (55·4 to 90·9)	−3·4% (−12·4 to 6·2)
Thailand		7·02 (6·36 to 7·78)	12·4% (11·3 to 13·7)	8·88 (7·98 to 9·92)	12·8% (11·5 to 14·4)	26·4% (16·0 to 39·8)	3·3% (−4·6 to 13·1)
	Male	3·67 (3·26 to 4·05)	13·0% (11·6 to 14·3)	4·22 (3·81 to 4·69)	13·1% (11·8 to 14·7)	14·9% (5·4 to 26·6)	1·3% (−6·2 to 9·8)
	Female	3·35 (3·02 to 3·74)	11·8% (10·7 to 13·1)	4·66 (4·12 to 5·29)	12·5% (11·0 to 14·2)	38·9% (25·0 to 56·1)	5·5% (−4·4 to 17·5)
Viet Nam		6·26 (5·61 to 6·94)	9·9% (9·1 to 11·0)	10·1 (9·09 to 11·3)	10·1% (9·1 to 11·3)	61·1% (48·3 to 76·0)	1·9% (−5·0 to 10·3)
	Male	2·73 (2·47 to 3·04)	8·9% (8·1 to 9·8)	4·62 (4·20 to 5·10)	9·5% (8·6 to 10·5)	69·3% (58·2 to 82·1)	6·3% (0·5 to 13·4)
	Female	3·53 (3·15 to 3·94)	10·8% (9·7 to 12·0)	5·46 (4·83 to 6·22)	10·7% (9·5 to 12·2)	54·7% (40·5 to 71·9)	−0·8% (−9·2 to 9·5)

Values in parentheses are 95% uncertainty intervals. Count data are given to three significant figures. ASEAN=Association of Southeast Asian Nations.

**Figure 1:**
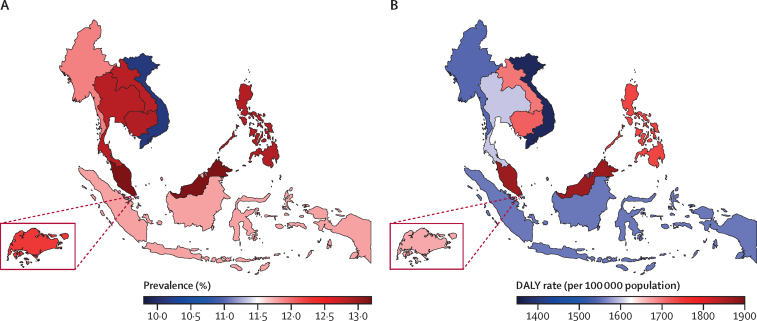
Age-standardised prevalence of and DALY rate attributable to mental disorders in the Association of Southeast Asian Nations, 2021 (A) Age-standardised prevalence. (B) Age-standardised DALY rate. DALY=disability-adjusted life-year.

With respect to age-standardised prevalence, an estimated 11·9% (95% UI 10·9–12·9) of the population of the ASEAN had a mental disorder in 2021 (table). Prevalence ranged from 10·1% (9·1–11·3) in Viet Nam to 13·2% (11·6–15·3) in Malaysia (figure 1A). In most ASEAN countries, the prevalence of mental disorders was higher in females than in males (table); however, the opposite was observed in Brunei (12·0% [10·8–13·4] for males *vs* 11·4% [10·0–13·2] for females), Singapore (12·8% [11·6–14·3] *vs* 11·7% [10·3–13·3]), and Thailand (13·1% [11·8–14·7] *vs* 12·5% [11·0–14·2]); appendix 1 p 17).

Between 1990 and 2021, the age-standardised prevalence across the ASEAN increased by 6·5% (95% UI 3·7–9·8; table). While most countries had relatively stable prevalence, increases were noted in Malaysia (12·6% [1·7–25·1]), Indonesia (9·0% [5·7–13·0]), and the Philippines (7·5% [5·2–10·3]). Across age groups, the largest increase in mental disorder prevalence was observed among people aged 15–19 years (appendix 1 p 18), where prevalence increased by 10·8% (6·9–15·1), from 12·2% (10·7–13·8) in 1990 to 13·6% (12·0–15·4) in 2021 (appendix 1 pp 23–26). The increase in prevalence was especially apparent between 2019 and 2021, coinciding with the COVID-19 pandemic (appendix 1 pp 23–26). Although the percentage change in the prevalence of mental disorders among adults aged 70 years and older was small, at 2·6% (–0·2 to 6·0), the relative increase in the number of cases was large, at 182·8% (174·9–192·1; appendix 1 p 18). Among the various conditions, anxiety disorders had the highest number of cases (29·1 million [24·5–34·5] cases), followed by depressive disorders (21·3 million [18·8–24·1]) and other mental disorders (10·3 million [8·01–12·9]; appendix 1 pp 27–28). These three conditions also had the highest age-standardised prevalence, estimated at 4·3% (3·6–5·1) for anxiety disorders, 3·1% (2·7–3·5) for depressive disorders, and 1·5% (1·2–1·9) for other mental disorders (appendix 1 pp 29–30). The seven remaining mental disorders all had a prevalence of less than 1%.

Variations existed between males and females in the prevalence of different mental disorders. The age-standardised prevalence of anxiety disorders was 1·7 times higher in females (5·4% [95% UI 4·5–6·4]) than in males (3·1% [2·6–3·7]; appendix 1 pp 19, 29–30). For depressive disorders, the prevalence in females (3·5% [3·1–4·0]) was approximately 1·3 times higher than that in males (2·7% [2·3–3·0]). For eating disorders, the prevalence among females (0·2% [0·1–0·2]) was 1·4 times higher than that among males (0·1% [0·1–0·2]). For idiopathic developmental intellectual disability, the prevalence among females (0·8% [0·4–1·2]) was also 1·4 times higher than that among males (0·6% [0·2–0·9]). By contrast, a higher prevalence was observed among males than females for ADHD (2·4 times higher), autism spectrum disorders (2·0 times higher), conduct disorder (1·6 times higher), and other mental disorders (1·5 times higher). Bipolar disorder had a similar age-standardised prevalence among males and females (both at 0·3% [0·3–0·4]), as did schizophrenia (0·3% [0·3–0·4] for males and 0·3% [0·2–0·3] for females).

Across age groups (appendix 1 pp 20, 31–34), the most prevalent mental disorders in children younger than 5 years were idiopathic developmental intellectual disability (0·9% [95% UI 0·4–1·5]) and autism spectrum disorders (0·9% [0·7–1·0]). In children aged 5–9 years, ADHD became the most prevalent disorder, with an estimate of 1·9% (1·3–2·8). Among individuals aged 10 years or older, anxiety disorders were the most prevalent disorder, estimated at 4·1% (2·8–5·7) in those aged 10–14 years, and peaking at 5·4% (3·9–7·2) in those aged 20–24 years (appendix 1 p 31–34). The prevalence of depressive disorders also increased from adolescence onward, rising from 1·1% (0·7–1·6) in those aged 10–14 years to a peak of 5·1% (4·2–6·1) in those aged 55–59 years. In adults aged 85–89 years or older, the prevalence of depressive disorders surpassed that of anxiety disorders, with the highest estimated prevalence of 4·9% (3·4–6·7) for depressive disorders found in those aged 95 years or older.

Anorexia nervosa was the only condition to which both mortality and YLLs could be attributed as the underlying cause of death. In 2021, the total number of deaths attributed to anorexia nervosa in the ASEAN was estimated at 3·4 (95% UI 1·5–5·4)—ie, between two and five individuals—which accounted for approximately 1·5% of the global deaths directly attributable to anorexia nervosa (appendix 1 p 35).^27^ The total number of YLLs associated with eating disorders in the ASEAN was estimated at 186·4 (81·8–295·1), which translates to a rate of 0·03 age-standardised YLLs (0·01–0·04) per 100 000 population (appendix 1 p 36).

In 2021, mental disorders were associated with 11·2 million (95% UI 8·54–14·3) DALYs, corresponding to an age-standardised DALY rate of 1583·7 (1203·3–2016·3) per 100 000 population in the ASEAN (appendix 1 pp 37–38). The total mental disorder-attributable DALYs in the ASEAN constituted 7·2% of the global DALYs associated with mental disorders (155 million [95% UI 117–198] DALYs globally; appendix 1 p 21).^3^ Between 1990 and 2021, the number of DALYs attributed to mental disorders in the ASEAN increased by 87·4% (81·1–94·0; appendix 1 pp 22, 37–38), and their proportion of the total disease burden rose by 62·8% (49·1–77·8), from 3·0% (2·4–3·7) to 4·9% (3·9–6·1; appendix 1 pp 39–40). The age-standardised DALY rate per 100 000 population, on the other hand, increased by 10·5% (7·6–13·6; appendix 1 pp 37–38).

Comparing across ASEAN countries, the total number of DALYs associated with mental disorders in 2021 (appendix 1 pp 37–38) was highest in Indonesia (4·58 million [95% UI 3·45–5·84]), the Philippines (1·96 million [1·48–2·50]), and Viet Nam (1·44 million [1·08–1·85]). When considering age-standardised DALY rates (figure 1B; appendix 1 pp 37–38), Malaysia had the highest rate with an estimate of 1866·7 DALYs (1377·2–2476·7) per 100 000 individuals, followed by the Philippines with 1738·8 (1305·4–2201·4) per 100 000, and Cambodia with 1717·3 (1278·9–2235·9) per 100 000. Between 1990 and 2021, the largest increase in the burden of mental disorders was observed in Malaysia, where the total number of DALYs rose by 133·6% (110·3–158·8; appendix 1 pp 22, 37–38). The Philippines, Laos, and Cambodia also had increases of more than 100% in the number of DALYs. This increase in burden coincided with continuous population growth in all of these countries.^33^ In terms of age-standardised DALY rates, Myanmar, Malaysia, and Indonesia had the largest increases, each over 12%, with more notable increases between 2019 and 2021 (appendix 1 pp 37–38).

**Figure 2:**
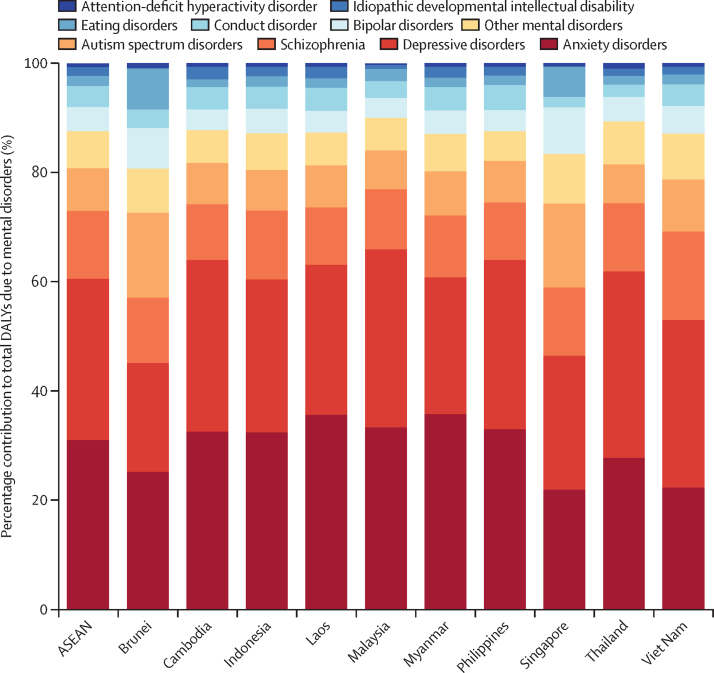
Relative contribution of DALYs attributable to Level 3 mental disorders to total mental disorder DALYs in the ASEAN and its member states, 2021 ASEAN=Association of Southeast Asian Nations. DALY=disability-adjusted life-year.

**Figure 3:**
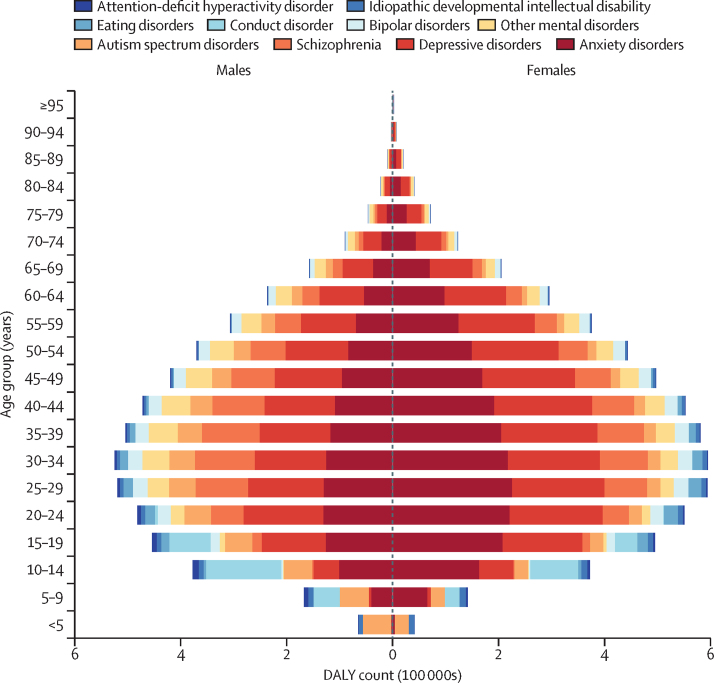
Number of DALYs by mental disorder, age, and sex in the Association of Southeast Asian Nations, 2021 DALY=disability-adjusted life-year.

The conditions contributing the most DALYs across ASEAN countries were anxiety disorders, accounting for 31·0% of the total DALYs due to mental disorders in the region, depressive disorders (29·6%), and schizophrenia (12·4%; figure 2; appendix 1 p 41). In females, anxiety disorders accounted for the largest proportion of DALYs among mental disorders, while depressive disorders were the leading contributors among males (figure 3; appendix 1 p 41). Among children and adolescents across ASEAN countries, the mental disorder burden was highest in those aged 10–14 years (16·3% [12·7–20·5] of total DALYs; appendix 1 pp 42–45), ranging from 13·8% of the total disease burden in Myanmar to over 20% in Malaysia (20·7%), Brunei (25·5%), and Singapore (28·2%).

**Figure 4:**
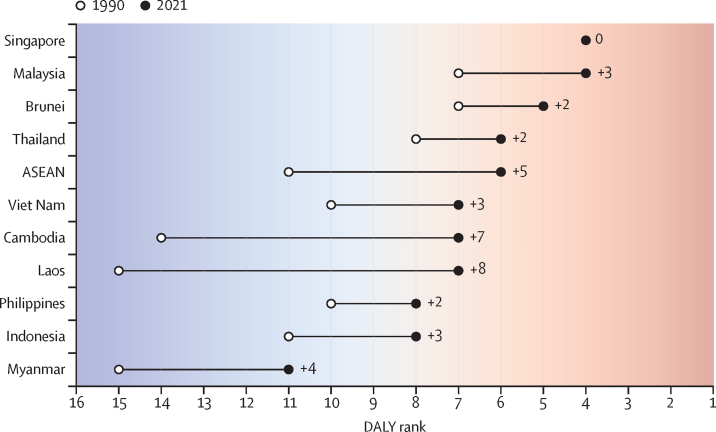
Rank of mental disorders among all Level 2 causes by number of DALYs, 1990 and 2021 The numbers represent the change in rank from 1990 to 2021. For example, in the ASEAN, mental disorders ranked 11th in 1990 and 6th in 2021, a +5 increase in rank. ASEAN=Association of Southeast Asian Nations. DALY=disability-adjusted life-year.

Overall, mental disorders were among the top ten causes of DALYs in 2021 in all ASEAN countries, except Myanmar, where they ranked 11th (figure 4). Mental disorders were in the top five major causes of disease burden in Singapore (contributing 9·2% [95% UI 7·6–10·8] of total DALYs), Brunei (7·6% [6·1–9·2]), and Malaysia (6·7% [5·3–8·4]) in 2021 (appendix 1 pp 39–40). The largest changes in the relative burden of mental disorders were seen in Laos (with rank increasing from 15th to seventh) and Cambodia (from 14th to seventh; figure 4). Between 1990 and 2021, the relative disease burden attributed to mental disorders rose from 1·4% (1·0–1·8) to 4·8% (3·4–6·3) in Laos and from 1·8% (1·4–2·2) to 4·9% (3·6–6·4) in Cambodia (appendix 1 pp 39–40). Upward shifts of at least two positions were observed in the DALY rankings of all countries except Singapore, where the rank of mental disorders remained unchanged.

## Discussion

To the best of our knowledge, this is the first study examining the prevalence and burden of mental disorders in the ASEAN. Our results indicated that in 2021, there were an estimated 80·4 million cases of mental disorders, reflecting an increase of approximately 70% since 1990. Age-standardised prevalence increased modestly by 6·5% during the same period, with a notable rise during the COVID-19 pandemic between 2019 and 2021. Across age groups, children and adolescents had the highest disease burden associated with mental disorders, whereas the number of cases increased most among older adults. Modest variability was observed across countries, with an overall age-standardised prevalence ranging from 10·1% in Viet Nam to 13·2% in Malaysia. The three most common mental disorders in the region were anxiety disorders, depressive disorders, and mental disorders categorised under “other mental disorders”. Overall, mental disorders ranked among the top ten causes of disease burden in all ASEAN countries except Myanmar, accounting for a fifth to a quarter of disease burden in children, adolescents, and young adults in several ASEAN countries, including Malaysia, Brunei, and Singapore.

The rise of mental disorders is driven by a complex interplay of individual, social, environmental, and structural factors.^34^ Some of the biggest drivers of mental disorders outlined in the 2018 *Lancet* Commission on global mental health and sustainable development^35^ pervade within the ASEAN, including poverty, food insecurity, and insufficient access to health care and education opportunities.^36^ The rapid socioeconomic development over the past decade has exacerbated inequalities in certain countries, deepening the rural–urban divide.^36^ Concurrently, political turmoil and violence in places such as Myanmar and the Philippines have triggered widespread trauma across the population.^15^, ^37^ Although not fully reflected in GBD 2021 estimates due to data limitations, the armed conflicts in Myanmar have had wide-ranging mental health implications.^15^, ^38^ The global surge in mental disorders due to the COVID-19 pandemic also affected ASEAN countries,^39^ despite the region's relatively low COVID-19 prevalence.^40^ Severe climate events have further heightened the population's susceptibility to mental disorders.^41^ In 2020, for instance, an estimated 2546·8 individuals per 100 000 population either died, went missing, or were directly affected by climate-related disasters.^42^ Certain parenting practices in the region, such as corporal punishment against children,^43^ might have had a detrimental effect on youth mental health, with long-term sequelae likely to persist into adulthood.^44^ Although improved mental health awareness and increased accessibility to screening and diagnosis could partly explain the rising prevalence of mental disorders,^16^, ^18^ the fundamental drivers of this public health crisis are the multifaceted socioenvironmental and structural determinants. These determinants demand a comprehensive, multisectoral, and intergovernmental approach to be properly addressed. As a geopolitical cooperative network with deep-rooted historical and cultural ties, the ASEAN should deploy collective efforts to foster regional stability, social progress, and resilience against environmental and climate threats, thereby safeguarding the mental wellbeing in the region.

In the past decade, many ASEAN countries have ramped up efforts to improve mental health care.^45^ The Philippines, for instance, adopted a Mental Health Act in 2018 to expand mental health services in collaboration with WHO.^17^ Indonesia extended full national health insurance coverage to mental disorders in 2014^46^ and launched a national initiative in 2010 to eliminate *pasung*—the traditional practice of secluding or physically restricting individuals with a mental illness, most commonly schizophrenia, for durations ranging from days to years.^18^ In Malaysia, new legislation implemented in 2010 brought compulsory mental health treatment up to international human rights standards.^47^ Although these policy changes are beginning to have a positive effect,^48^ social stigma and cultural barriers remain widespread,^18^, ^23^ and there is a great reluctance to seek professional care.^49^, ^50^ Even in Singapore, ASEAN's highest-income country,^10^ about 78·6% of adults with mental disorders were without appropriate mental health care according to a study from 2016.^24^ Since COVID-19, several ASEAN countries have launched targeted campaigns to encourage mental health care-seeking behaviours.^51^, ^52^, ^53^ There has also been a deliberate effort to enhance access to care by shifting mental health services from institutional to community settings.^54^, ^55^ However, the shortage of mental health professionals remains a prevailing challenge across the ASEAN.^45^ Psychiatrist-to-population ratios in the ASEAN range from 0·2 per 100 000 population in Myanmar and the Philippines to 3·7 per 100 000 in Brunei and 4·3 per 100 000 in Singapore, remaining well below the European average of 9·7 per 100 000.^56^ These shared challenges underscore the need for multilateral, multisectoral collaboration within the ASEAN to strengthen mental health research capacity, generating insights to inform best practices adaptable across diverse settings.^12^ Successful examples of such approaches exist in other regions.^57^ Nevertheless, actualising such an effort is a formidable undertaking, which requires strong political will and devoted stewards.

Consistent with the global literature,^58^ depressive disorders, anxiety disorders, and eating disorders were more prevalent among females than males in the ASEAN. These differences have been associated with a combination of biological, psychological, and environmental factors, such as exposure to childhood sexual abuse, intimate partner violence, and structural gender inequalities.^59^ Intimate partner violence against women is widespread within the ASEAN, with a lifetime prevalence ranging from 15% to 44% in six member states (Cambodia, Indonesia, Myanmar, the Philippines, Thailand, and Viet Nam).^60^ In Myanmar and Brunei, there is no specific law against domestic violence.^60^ Dedicated programmes and resources protecting women and girls against maltreatment are insufficient in many ASEAN countries, and gender inequality perpetrates across a wide range of areas, from gender preference at birth, to education and access to health care.^61^ This vulnerable situation for females calls for more research on the determinants of sex differences in mental health in the region to identify proper countermeasures.

The growing effect of mental disorders on adolescents is particularly concerning. Mental disorders constituted around a quarter of the total disease burden in those aged 10–19 years in high-income ASEAN countries, namely Brunei and Singapore. The age-specific prevalence of mental disorders had increased by more than 10% in those aged 15–19 years between 1990 and 2021, with a notable rise between 2019 and 2021. The trends observed in the ASEAN align with global trends of increasing psychological suffering in these age groups,^62^ partly attributed to social media use.^63^ Within the context of the ASEAN, however, other fundamental stressors, such as hunger, poverty, and low educational attainment might also have contributed.^64^, ^65^ In a 2017 school-based survey, over 55% of youth in the ASEAN reported experiencing hunger, the frequency of which is associated with psychological distress.^64^, ^65^ In 2021, the ASEAN, China, Japan, and South Korea reaffirmed their commitment to cooperation on mental health among adolescents and young children.^66^ The actualisation of these visions and missions is becoming increasingly urgent to avert the long-lasting effects of mental disorders on younger generations.

The prevalence of mental disorders among older adults is another pressing public health issue within the ASEAN, where the proportion of the population older than 60 years has been projected to exceed 22% by 2050.^67^ Our results indicate that, driven by longer life expectancy and an increasingly older population, the number of mental disorder cases in older adults has risen by 182·8% over the past 30 years, despite stagnant prevalence rates. The Kuala Lumpur Declaration on Ageing,^68^ adopted by all ASEAN member states in 2015, and the subsequent action plan developed in 2020^69^ provided a general framework to promote active and healthy ageing, but did not explicitly address mental health. In 2024, another ASEAN report focusing on the promotion of a healthy lifestyle recognised the need to improve both mental health and healthy ageing, again without mentioning the integration of these two objectives into joint policies.^70^ Mental disorders in older adults can indirectly precipitate physical illness and disability through self-neglect and self-harm.^71^, ^72^ Interventions and policies to protect the mental wellbeing of older adults are therefore indispensable in any national or regional healthy ageing strategy.

This study has a number of limitations. First, due to specific adjustments made to account for the effect of COVID-19 in GBD 2021, most changes in the prevalence and burden of mental disorders over time occurred between 2019 and 2021, primarily driven by the estimated increase in the prevalence and burden of major depressive disorder and anxiety disorders. For most disorders, there were insufficient data to model temporal changes across time and geographies between 1990 and 2019. As a result, many of the between-country differences in rates and changes over time were probably influenced by the pandemic adjustment. Countries more severely affected by the pandemic would have had larger increases than those less affected. Second, the estimates for certain countries and age groups have high uncertainty, as nationally representative epidemiological data on mental disorders was scarce or absent.^56^ In particular, among children and adolescents, mental health data are not routinely collected. For instance, Singapore only recently launched its first national psychiatric epidemiological study among youths in 2022.^73^ In other countries, national data on mental health are intermittently collected, with a higher scarcity of available data from before the COVID-19 pandemic.^74^ Considering the increase in mental disorder prevalence and burden, it is crucial for governments in the region to invest in better mental health screening and surveillance. Third, the rise in mental disorder cases is driven by multiple factors beyond the actual increase in disease onset, including demographic shifts, improved access to screening services, and advancements in diagnostic tools. In this study, the contrast between prevalent cases and age-standardised prevalence offers a glimpse into the variability potentially driven by population changes. However, future research could explore a more detailed decomposition of these underlying drivers. Fourth, in GBD, mental disorders are defined using either survey responses on the frequency of symptoms or clinical definitions based on DSM and ICD criteria. Given the low level of mental health literacy^21^, ^22^ and pervasive underdiagnosis^20^ in the region, the reported prevalence and burden probably underestimated the actual situation. Clinical definitions of mental disorders might not always be understood in the sociocultural context of the ASEAN, where some subpopulations tend to describe mental suffering in terms of physical rather than emotional pain or understand it in terms of their own spiritual beliefs.^75^ Finally, the burden of premature mortality was only estimated for anorexia nervosa. Mental disorders can result in premature mortality through self-harm;^76^ however, the current GBD framework does not account for this mediation effect, which, if considered, could potentially lead to higher estimated mortality.^1^

Our study highlights an important increase in the prevalence of mental disorders and the associated burden in ASEAN countries. Whereas national and international organisations have already made efforts to curb this trend in most of these nations, much remains to be done to overcome existing challenges as a region at the environmental, social, and structural levels. Interest in mental health is nonetheless growing within ASEAN nations, as evidenced by increasing national and regional research, which promises to result in a more precise understanding of the mental health landscape and subsequent evidence-based policy-making.

### GBD 2021 ASEAN Mental Disorders Collaborators

### Affiliations

### Contributors

### Data sharing

To download the input data used, and estimates generated in these analyses, please visit the Global Health Data Exchange GBD 2021 website at https://ghdx.healthdata.org/gbd-2021. Codes used for the analysis can be found at https://ghdx.healthdata.org/gbd-2021/code.

## Declaration of interests

Q E S Adnani reports Online Library Data Research funds from Universitas Padjadjaran, Bandung, Indonesia, under contract number 2152/UN6.3.1/PT.00/2024 and University Scientific Excellence Research funds from Universitas Padjadjaran, Bandung, Indonesia, 2025, outside the submitted work. N E Ismail reports leadership or fiduciary roles in board, society, committee, or advocacy groups, unpaid, with Malaysian Academy of Pharmacy as Bursar and Council Member and with Malaysian Pharmacists Society as Committee Member of Education Chapter; all outside the submitted work. R J Maude reports support for the present manuscript from Wellcome Trust (grant number 220211), which provides core funding for Mahidol Oxford Tropical Medicine Research and contributes to their salary. J L Samodra reports grants or contracts from NSTC Taiwan and Taipei Medical University, Taiwan; consulting fees from FK Unpar, Bandung, Indonesia; leadership or fiduciary roles in board, society, committee or advocacy groups, paid or unpaid with Benang Merah Research Center as Co-founder and Director; other support from Jago Beasiswa as a mentor; all outside the submitted work.
