# Supplementary material for: The epidemiology and burden of ten mental disorders in countries of the Association of Southeast Asian Nations (ASEAN), 1990–2021: findings from the Global Burden of Disease Study 2021
Source: Lancet Public Health. 2025 May 27;10(6):e480–91. doi: 10.1016/S2468-2667(25)00098-2 (PMC12127263; doi:10.1016/S2468-2667(25)00098-2)
Supplement: Supplementary appendix [file mmc1.pdf]

THE LANCET
Public Health

Supplementary appendix 1

This appendix formed part of the original submission and has been peer reviewed. We post it as supplied by the authors.

Supplement to: GBD 2021 ASEAN Mental Disorders Collaborators. The epidemiology and burden of ten mental disorders in countries of the Association of Southeast Asian Nations (ASEAN), 1990–2021: findings from the Global Burden of Disease Study 2021. *Lancet Public Health* 2025; **10:** e480–91.

Appendix 1

Supplement to: The epidemiology and burden of ten mental disorders in countries of the Association of Southeast Asian Nations: findings from the Global Burden of Disease Study 2021

Preamble

This appendix provides further methodological detail for “The epidemiology and burden of ten mental disorders in the Association of Southeast Asian Nations (ASEAN): A systematic analysis of the Global Burden of Disease Study 2021”.

Portions of this appendix have been reproduced or adapted from appendices of the following publications:

Ferrari AJ, Santomauro DF, Aali A, *et al*. Global incidence, prevalence, years lived with disability (YLDs), disability-adjusted life-years (DALYs), and healthy life expectancy (HALE) for 371 diseases and injuries in 204 countries and territories and 811 subnational locations, 1990–2021: a systematic analysis for the Global Burden of Disease Study 2021. *The Lancet* 2024; **403**: 2133-61. DOI:10.1016/S0140-6736(24)00757-8.

GBD 2019 Mental Disorders Collaborators. Global, regional, and national burden of 12 mental disorders in 204 countries and territories, 1990–2019: a systematic analysis for the Global Burden of Disease Study 2019. *The Lancet Psychiatry* 2022; **9**: 137–50.

Contents

Appendix 1

Preamble 2

Supplemental methods 4

Data sources specific to ASEAN countries 4

Model flow charts for each mental disorder based on GBD 2021 7

Mental disorders included in the GBD 2021 Study 13

Data selection considerations 14

Statistical analysis 14

Supplementary Results 16

GATHER Checklist 46

References 47

Supplemental methods

Data sources specific to ASEAN countries

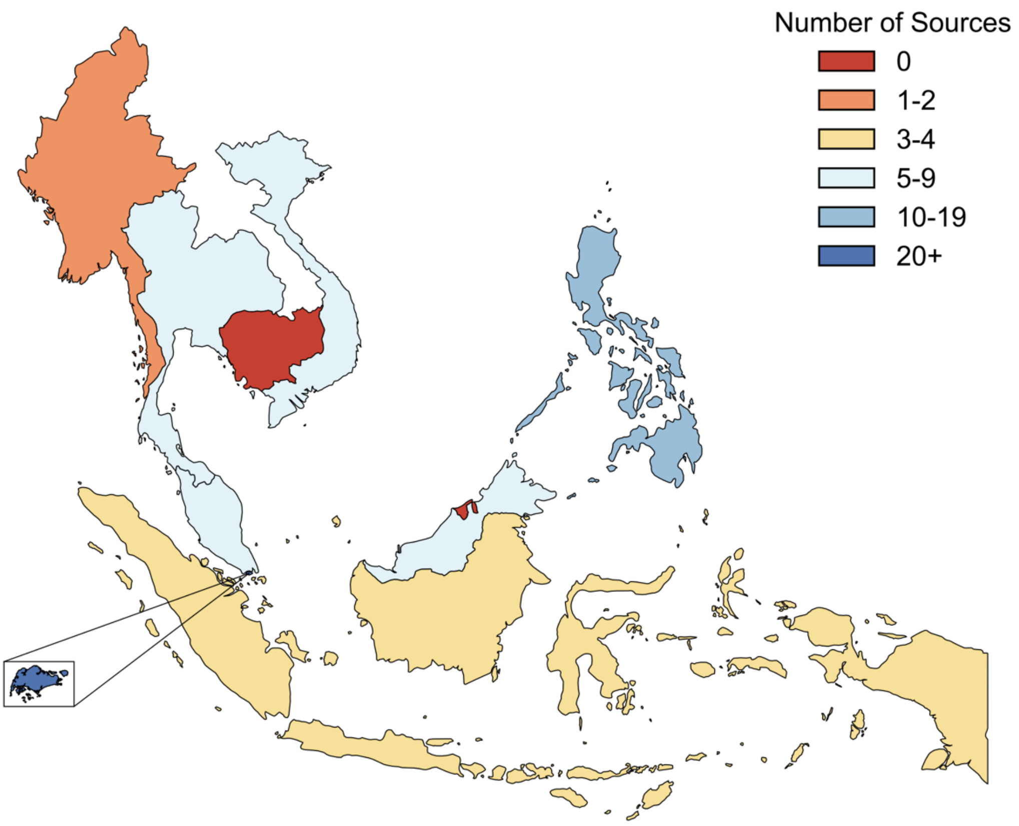

Figure S1 | Number of data sources used to estimate mental disorder prevalence 1990-2021.

Table S1 | Data sources used for estimation of mental disorder prevalences across the 10 ASEAN countries.

**Brunei**	
No data sources reported	
**Cambodia**	
No data sources reported	
**Indonesia**	
**Non-fatal health outcomes**	
1	Wignyosumarto S, Mukhlas M, Shirataki S. Epidemiological and clinical study of autistic children in Yogyakarta, Indonesia. Kobe J Med Sci. 1992; 38(1): 1-19.
2	Peltzer K, Pengpid S. High prevalence of depressive symptoms in a national sample of adults in Indonesia: Childhood adversity, sociodemographic factors and health risk behaviour. Asian J Psychiatr. 2018; 33: 52-59.
3	Kurihara T, Kato M, Reverger R, Tirta IGR. Seventeen-year clinical outcome of schizophrenia in Bali. Eur Psychiatry. 2011; 26(5): 333-8.
**Laos**	
**Non-fatal health outcomes**	
1	Phanthavong P, Naphayvong P, Reinharz D. Depression among last-year high school students in Vientiane, capital city of Lao PDR. Asia Pac J Public Health. 2015; 27(2): NP1995-1998.
2	World Health Organization (WHO). Laos World Health Survey 2003.
3	Charlson F, Choulamany C, Diminic S, Santomauro D, Raja S, Whiteford H. The prevalence of mental and substance use disorders in Lao PDR: findings from a cross-sectional survey. [Forthcoming]
**Malaysia**	
**Non-fatal health outcomes**	
1	Kasmini K KO, Krishnaswamy S, Ramli H, Hassan S. A prevalence survey of mental disorders among children in a rural Malaysian village. Acta Psychiatr Scand. 1993; 87(4): 253-7.
2	Krishnaswamy S, Kavitha S, Aziz JA, Indran H, Low WY, Ramachandran P, Indran T, Indran R, Patel V. Burden, Determinant and Impact of Common Mental Disorders in Malaysia. [Unpublished].
3	Institute for Public Health, Ministry of Health (Malaysia). Malaysia National Health and Morbidity Survey 2011.
4	World Health Organization (WHO). Malaysia World Health Survey 2003. Geneva, Switzerland: World Health Organization (WHO), 2005.
5	Stein Z, Belmont L, Durkin M. Mild mental retardation and severe mental retardation compared: experiences in eight less developed countries. Ups J Med Sci Suppl. 1987; 44: 89-96.
6	Kaur J, Cheong SM, Mahadir Naidu B, Kaur G, Manickam MA, Mat Noor M, Ibrahim N, Rosman A. Prevalence and correlates of depression among adolescents in Malaysia. Asia Pac J Public Health. 2014; 26(5 Suppl): 53S–62S.
7	Kader Maideen SF, Sidik SM, Rampal L, Mukhtar F. Prevalence, associated factors and predictors of depression among adults in the community of Selangor, Malaysia. PLoS One. 2014; 9(4): e95395.
**Myanmar**	
**Non-fatal health outcomes**	
1	World Health Organization (WHO). Myanmar World Health Survey 2003. Geneva, Switzerland: World Health Organization (WHO), 2005.
**Philippines**	
**Non-fatal health outcomes**	
1	Stein Z, Belmont L, Durkin M. Mild mental retardation and severe mental retardation compared: experiences in eight less developed countries. Ups J Med Sci Suppl. 1987; 44: 89-96.
2	World Health Organization (WHO). Philippines World Health Survey 2003. Geneva, Switzerland: World Health Organization (WHO), 2005.
**Causes of death (eating disorders only)**	
1	National Statistics Office (Philippines). Philippines Vital Registration - Deaths 2006.
2	National Statistics Office (Philippines). Philippines Vital Registration - Deaths 2007.
3	National Statistics Office (Philippines). Philippines Vital Registration - Deaths 2008.
4	National Statistics Office (Philippines). Philippines Vital Registration - Deaths 2009.
5	National Statistics Office (Philippines). Philippines Vital Registration - Deaths 2010.
6	National Statistics Office (Philippines). Philippines Vital Registration - Deaths 2011.
7	National Statistics Office (Philippines). Philippines Vital Registration - Deaths 2012.
8	Philippines Statistics Authority. Philippines Vital Registration - Deaths 2013.
9	Philippines Statistics Authority. Philippines Vital Registration - Deaths 2014.
10	Philippines Statistics Authority. Philippines Vital Registration - Deaths 2015.
11	Philippines Statistics Authority. Philippines Vital Registration - Deaths 2016.
12	Philippines Statistics Authority. Philippines Vital Registration - Deaths 2017.
13	Philippines Statistics Authority. Philippines Vital Registration - Deaths 2018.
**Singapore**	
**Non-fatal health outcomes**	
1	Chong SA, Abdin E, Vaingankar JA, Heng D, Sherbourne C, Yap M, Lim YW, Wong HB, Ghosh-Dastidar B, Kwok KW, Subramaniam M. A population-based survey of mental disorders in Singapore. Ann Acad Med Singap. 2012; 41(2): 49-66.
2	Ho CS, Jin A, Nyunt MSZ, Feng L, Ng TP. Mortality rates in major and subthreshold depression: 10-year follow-up of a Singaporean population cohort of older adults. Postgrad Med. 2016; 128(7): 642–7.
3	Subramaniam M, Verma S, Cheok C, Lee I-M, Wong J, Chong SA. Prevalence and correlates of psychotic symptoms among Asian males. Soc Psychiatry Psychiatr Epidemiol. 2012; 47(1): 137–44.
4	Subramaniam M, Abdin E, Sambasivam R, Vaingankar JA, Picco L, Pang S, Seow E, Chua BY, Magadi H, Mahendran R, Chong SA. Prevalence of Depression among Older Adults-Results from the Well-being of the Singapore Elderly Study. Ann Acad Med Singap. 2016; 45(4): 123-33.
5	Subramaniam M, Abdin E, Vaingankar JA, Chong SA. Prevalence, correlates, comorbidity and severity of bipolar disorder: results from the Singapore Mental Health Study. J Affect Disord. 2013; 146(2): 189–96.

6	Institute of Mental Health (Singapore). Singapore National Mental Health Study 2016-2018.
7	Fones CS KE, Ng TP, Ko SM. Studying the mental health of a nation: a preliminary report on a population survey in Singapore. Singapore Med J. 1998; 39(6): 251-5.
8	Subramaniam M, Abdin E, Vaingankar JA, Shafie S, Chua BY, Sambasivam R, Zhang YJ, Shahwan S, Chang S, Chua HC, Verma S, James L, Kwok KW, Heng D, Chong SA. Tracking the mental health of a nation: prevalence and correlates of mental disorders in the second Singapore mental health study. Epidemiol Psychiatr Sci. 2019; 1-10.
**Causes of death (eating disorders only)**	
1	Ministry of Health (Singapore). Singapore Causes of Death 1980.
2	Ministry of Health (Singapore). Singapore Causes of Death 1981.
3	Ministry of Health (Singapore). Singapore Causes of Death 1982.
4	Ministry of Health (Singapore). Singapore Causes of Death 1983.
5	Ministry of Health (Singapore). Singapore Causes of Death 1984.
6	Ministry of Health (Singapore). Singapore Causes of Death 1985.
7	Ministry of Health (Singapore). Singapore Causes of Death 1986.
8	Ministry of Health (Singapore). Singapore Causes of Death 1987.
9	Ministry of Health (Singapore). Singapore Causes of Death 1988.
10	Ministry of Health (Singapore). Singapore Causes of Death 1989.
11	Ministry of Health (Singapore). Singapore Causes of Death 1990.
12	Ministry of Health (Singapore). Singapore Causes of Death 1991.
13	Ministry of Health (Singapore). Singapore Causes of Death 1992.
14	Ministry of Health (Singapore). Singapore Causes of Death 1993.
15	Ministry of Health (Singapore). Singapore Causes of Death 1994.
16	Ministry of Health (Singapore). Singapore Causes of Death 1995.
17	Ministry of Health (Singapore). Singapore Causes of Death 1996.
18	Ministry of Health (Singapore). Singapore Causes of Death 1997.
19	Ministry of Health (Singapore). Singapore Causes of Death 1998.
20	Ministry of Health (Singapore). Singapore Causes of Death 1999.
21	Ministry of Health (Singapore). Singapore Causes of Death 2000.
22	Ministry of Health (Singapore). Singapore Causes of Death 2001.
23	Ministry of Health (Singapore). Singapore Causes of Death 2002.
24	Ministry of Health (Singapore). Singapore Causes of Death 2003.
25	Ministry of Health (Singapore). Singapore Causes of Death 2004.
26	Ministry of Health (Singapore). Singapore Causes of Death 2005.
27	Ministry of Health (Singapore). Singapore Causes of Death 2006.
28	Ministry of Health (Singapore). Singapore Causes of Death 2007.
29	Ministry of Health (Singapore). Singapore Causes of Death 2008.
30	Ministry of Health (Singapore). Singapore Causes of Death 2009.
31	Ministry of Health (Singapore). Singapore Causes of Death 2010.
32	Ministry of Health (Singapore). Singapore Causes of Death 2011.
33	Ministry of Health (Singapore). Singapore Causes of Death 2012.
34	Ministry of Health (Singapore). Singapore Causes of Death 2013.
35	Ministry of Health (Singapore). Singapore Causes of Death 2014.
36	Ministry of Health (Singapore). Singapore Causes of Death 2015.
37	Ministry of Health (Singapore). Singapore Causes of Death 2016.
38	Ministry of Health (Singapore). Singapore Causes of Death 2017.
39	World Health Organization (WHO). Singapore Vital Registration - Deaths 2019 ICD10. as it appears in WHO Mortality Database Version March 2021
**Thailand**	
**Non-fatal health outcomes**	
1	Benjasuwantep B RN, Visudhiphan P. Prevalence and clinical characteristics of attention deficit hyperactivity disorder among primary school students in Bangkok. J Med Assoc Thai. 2002; 85(Suppl 4): 1232-40.
2	Wacharasindhu A, Panyyayong B. Psychiatric disorders in Thai school-aged children: I Prevalence. J Med Assoc Thai. 2002; S125-136.
3	Phanthunane P, Vos T, Whiteford H, Bertram M, Udomratn P. Schizophrenia in Thailand: prevalence and burden of disease. Popul Health Metr. 2010; 8: 24.
4	Ministry of Public Health (Thailand). Thailand Burden of Disease and Injuries 1998-1999.
**Viet Nam**	
**Non-fatal health outcomes**	
1	Pollack AA, Weiss B, Trung LT. Mental health, life functioning and risk factors among people exposed to frequent natural disasters and chronic poverty in Vietnam. Br J Psychiatr Open. 2016; 2(3): 221–32.
2	Hoang VM, Le TV, Chu TTQ, Le BN, Duong MD, Thanh NM, Tac Pham V, Minas H, Bui TTH. Prevalence of autism spectrum disorders and their relation to selected socio-demographic factors among children aged 18-30months in northern Vietnam, 2017. Int J Ment Health Syst. 2019; 13: 29.
3	Hanoi School of Public Health, Ministry of Health (Vietnam), School of Population Health, University of Queensland (Australia). Vietnam Burden of Disease and Injury Study 2008.
4	Ministry of Health (Vietnam), University of Queensland (Australia). Vietnam Evidence Base for Health Policy (VINE) Project 2006-2010.
5	World Health Organization (WHO). Vietnam World Health Survey 2002-2003. Geneva, Switzerland: World Health Organization (WHO), 2005.

Note: For more details about data sources, see: https://ghdx.healthdata.org/gbd-2021/sources

Model flow charts for each mental disorder based on GBD 2021

Depressive disorders

Major depressive disorder

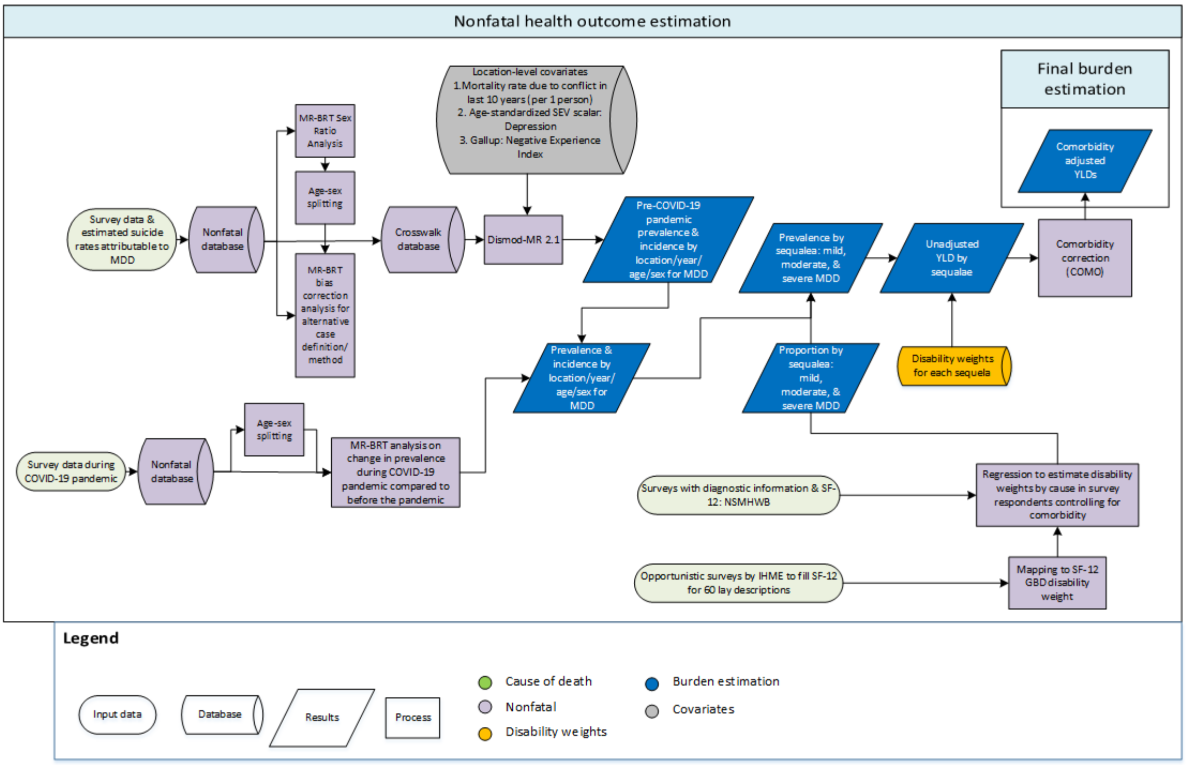

Dysthymia

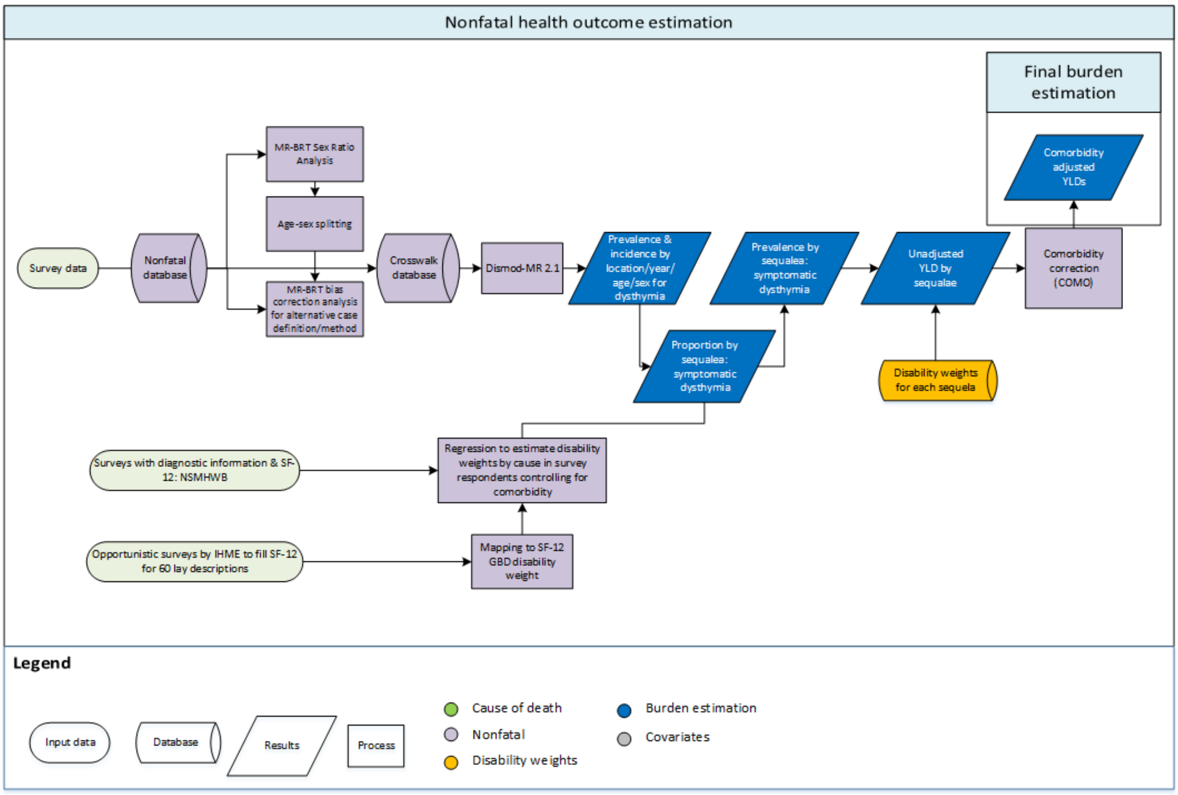

Anxiety disorders

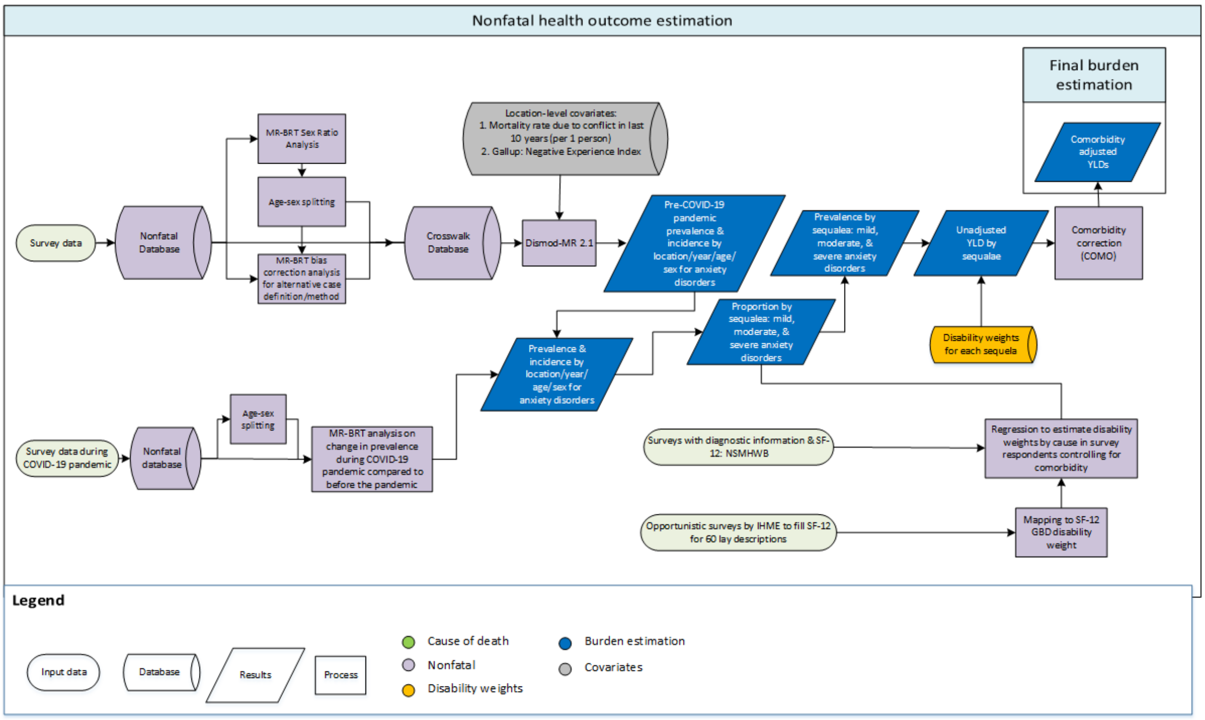

Bipolar disorders

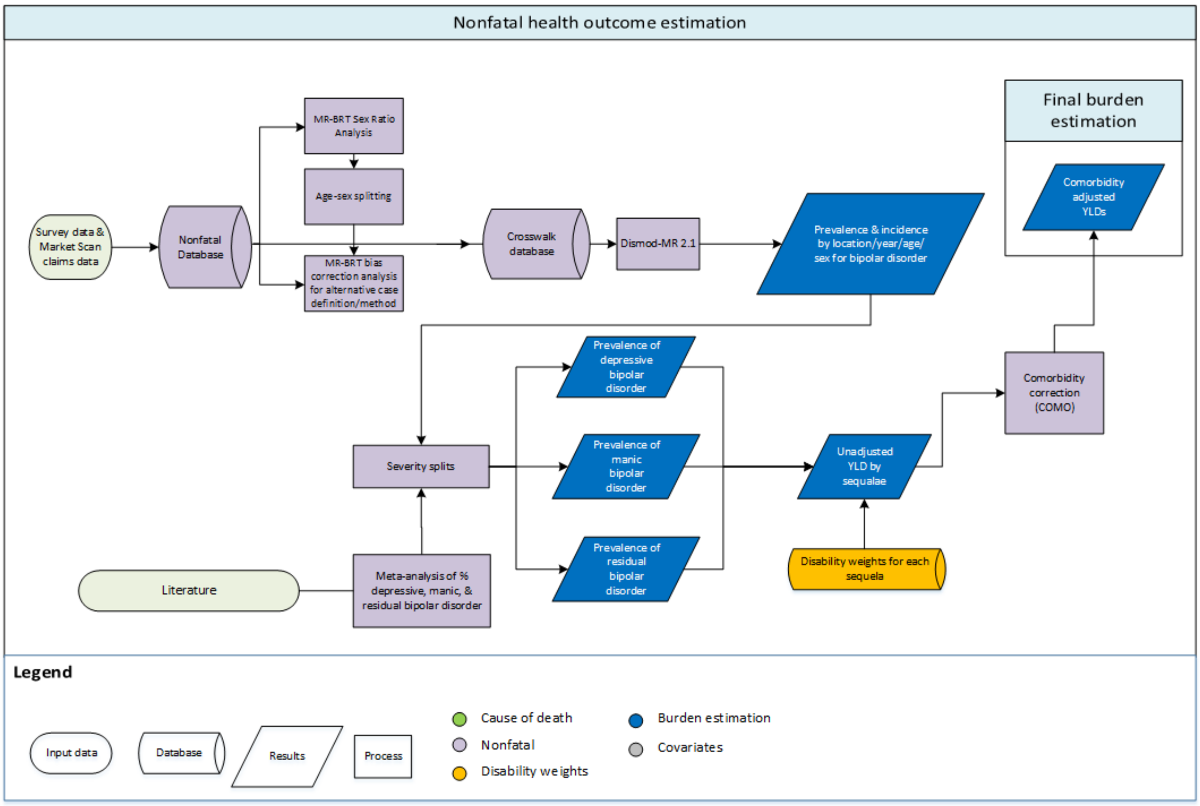

Schizophrenia

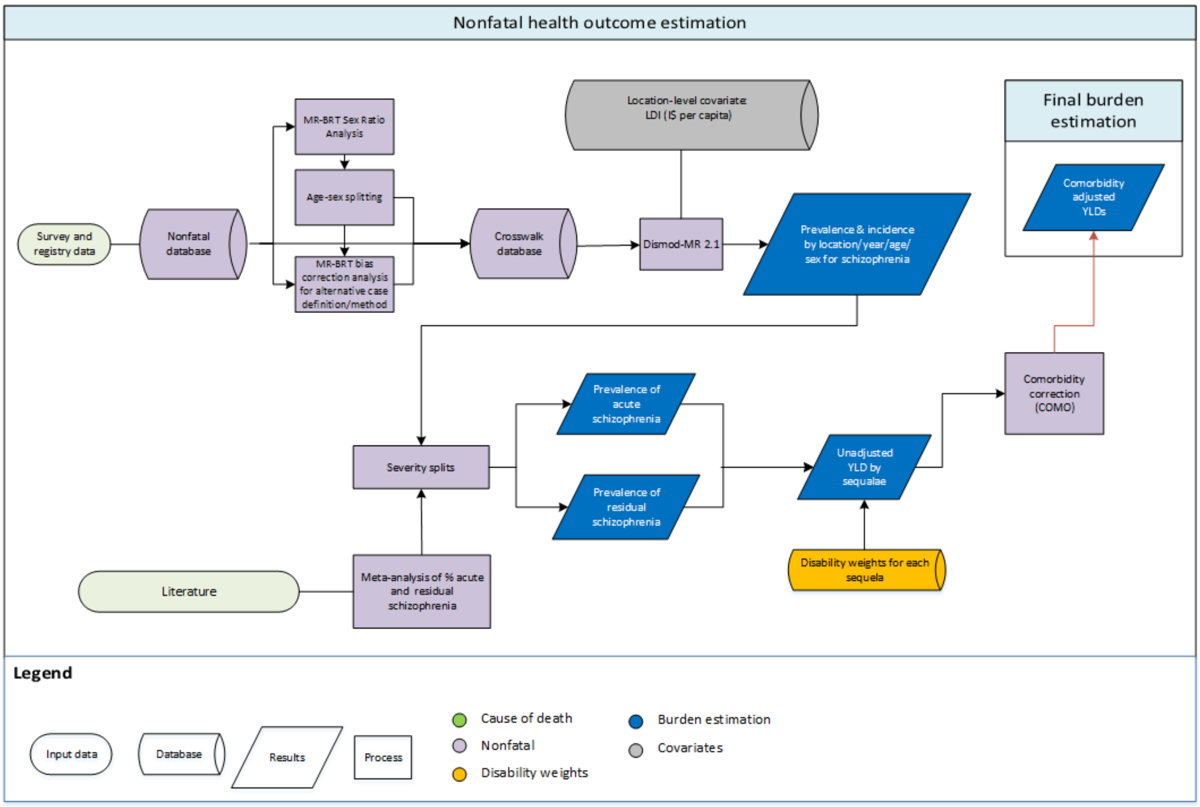

Autism spectrum disorders

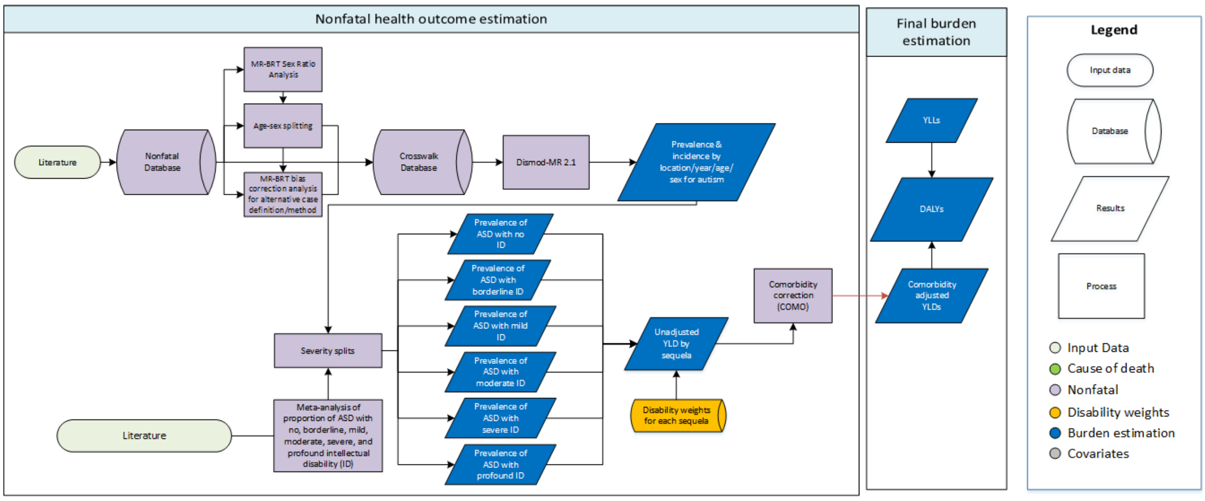

Conduct disorder

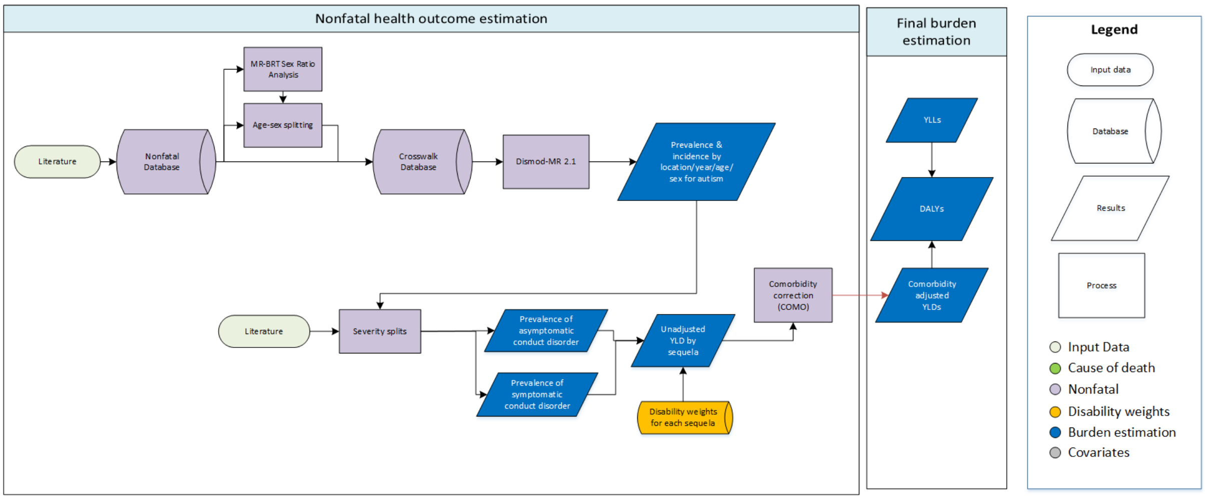

Attention-deficit hyperactivity disorder (ADHD)

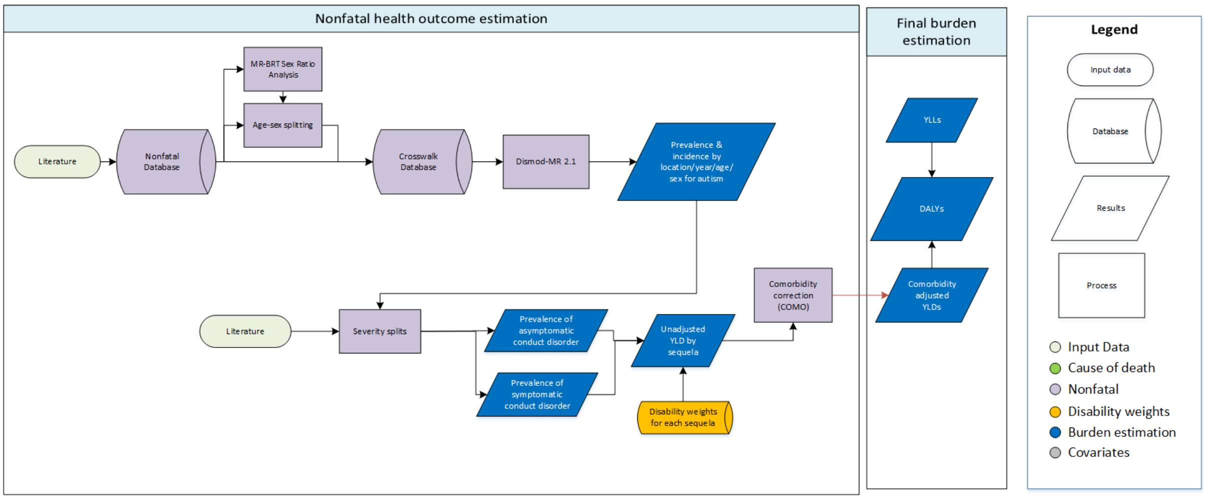

Eating disorders

Anorexia nervosa

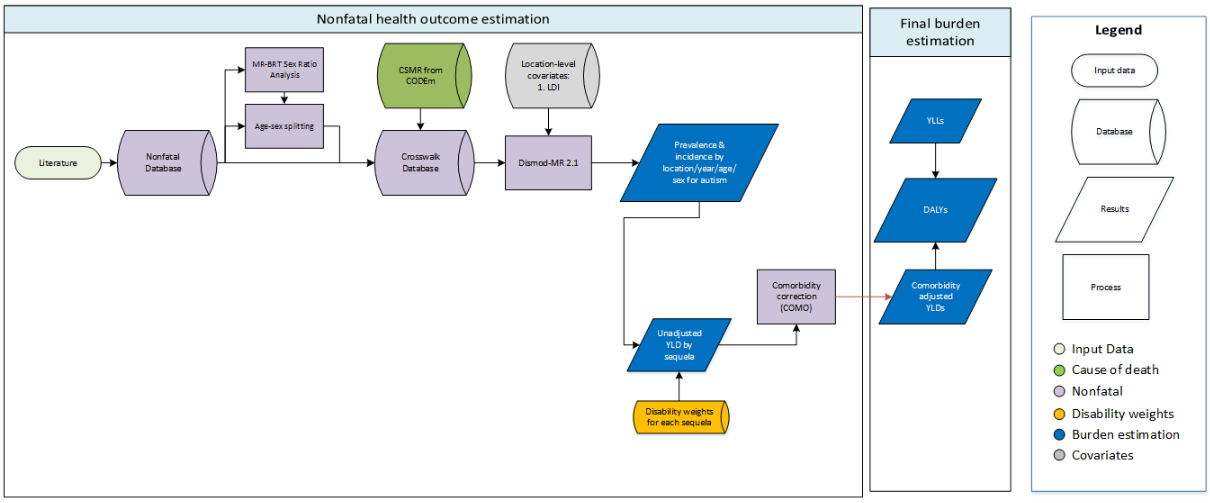

Bulimia nervosa

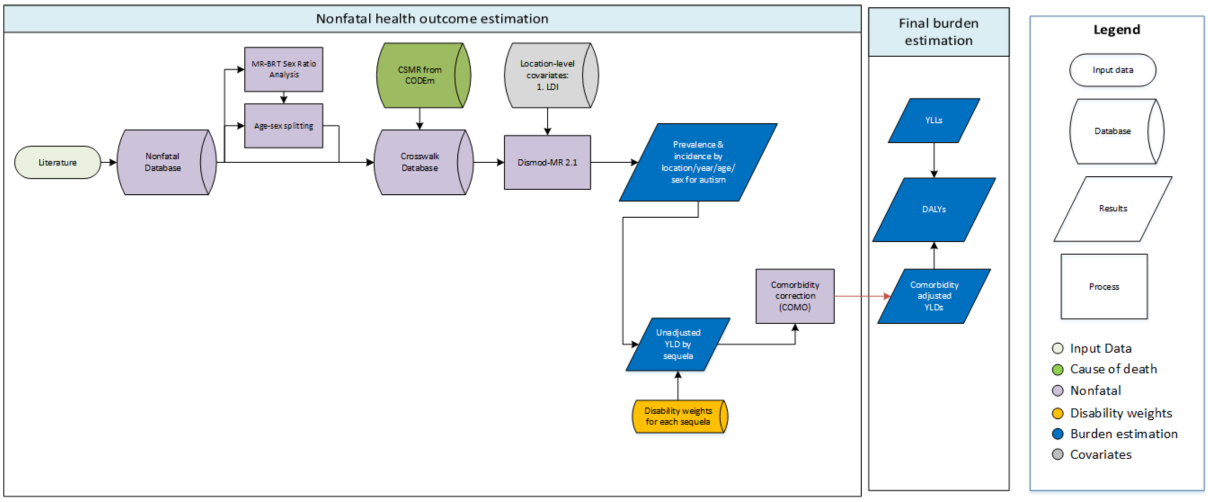

Idiopathic developmental intellectual disability (estimated as part of a broader intellectual disability category)

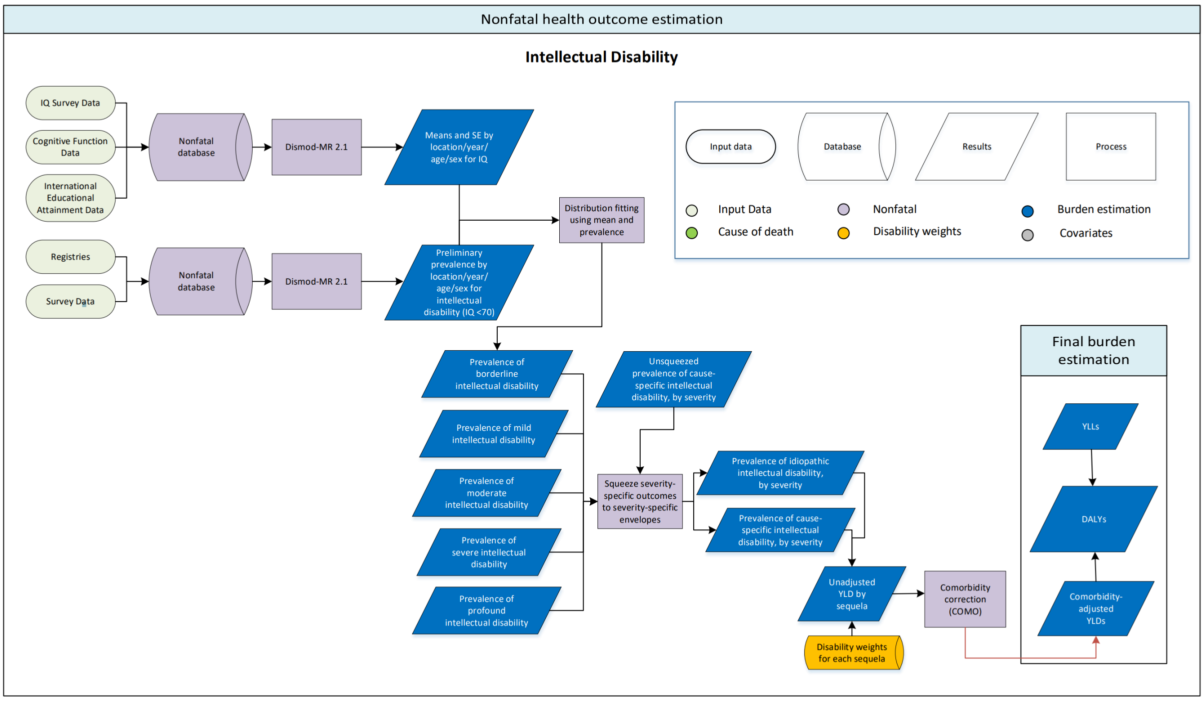

Other mental disorders

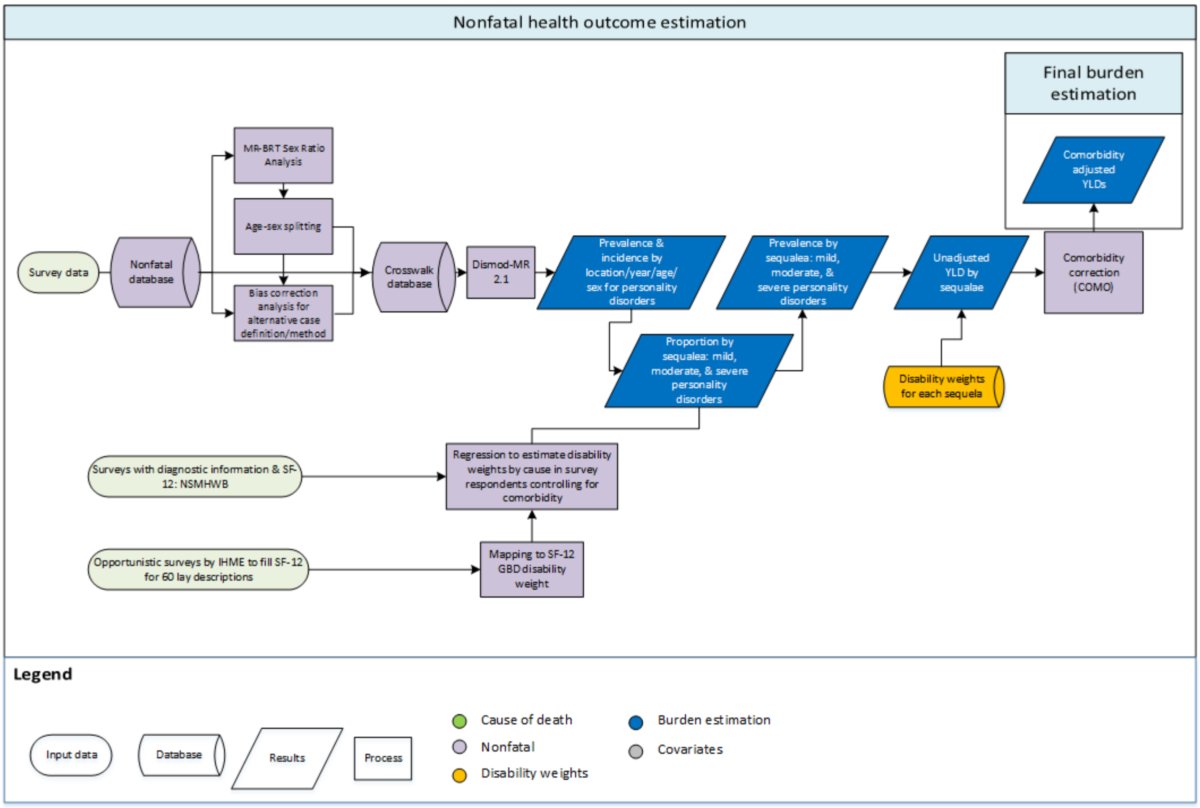

Mental disorders included in the GBD 2021 Study

Note: Diagnostic codes are indicated for DSM-IV-TR and ICD-10, but corresponding diagnoses referencing other versions of DSM (DSM-III, DSM-III-R, DSM-IV, DSM-5, and DSM-5-TR) or ICD (ICD-9, ICD-11) were also accepted.

Cause	Definition	Corresponding DSM-IV-TR and ICD-10 codes
**Major depressive disorder**	Involves the presence of at least one major depressive episode, which is the experience of either depressed mood or loss of interest/pleasure, for most of every day, for at least two weeks.	**DSM-IV-TR**: 296.21–24, 296.31–34<br>**ICD-10**: F32.0–9, F33.0–9
**Dysthymia**	Involves the experience of chronically depressed mood for most of the day, more days than not, for at least two years (or at least one year in children and adolescents).	**DSM-IV-TR**: 300.4<br>**ICD-10**: F34.1
**Anxiety disorders**	Involves experiences of intense fear and distress, typically in combination with other physiological symptoms. Anxiety disorders were modelled as a single cause for "any" anxiety disorder to avoid the double-counting of individuals meeting criteria for more than one anxiety disorder. Epidemiological estimates reporting an outcome for "any" or "total" anxiety disorders were included if they reported on at least three anxiety disorders.	**DSM-IV-TR**: 300.0-300.3, 208.3, 309.21, 309.81<br>**ICD-10**: F40-42, F43.0, F43.1, F93.0-93.2, F93.8.
**Schizophrenia**	Involves the experience of positive symptoms (e.g., delusions, hallucinations, thought disorder) and negative symptoms (e.g., flat affect, loss of interest, and emotional withdrawal).	**DSM-IV-TR**: 295.10-295.30, 295.60, 295.90<br>**ICD-10**: F20
**Bipolar disorder**	Bipolar I disorder is characterised by at least one manic episode, which can also alternate with a major depressive episode. Bipolar II disorder is characterised by hypomanic episodes alternating with major depressive episodes. Cyclothymia is characterised by subsyndromal hypomanic and major depressive episodes. Bipolar disorder not otherwise specified is characterised by clinically significant symptoms of bipolar disorder which do not meet the criteria for the other diagnoses. We estimated the burden for the entire spectrum of bipolar disorder simultaneously, rather than individually for each subtype of the disorder. At a minimum, epidemiological studies are needed to report on bipolar I and bipolar II disorders.	**DSM-IV-TR**: 296.0–296.7, 296.89, 301.13<br>**ICD-10**: F30.0-F30.9, F31.0–F31.6, F31.8–F31.9, F34.0
**Anorexia nervosa**	Characterised by refusal to maintain body weight at or above a minimally normal weight for age and height, intense fear of gaining weight, and disturbance in the way in which one's body weight or shape is experienced.	**DSM-IV-TR**: 307.1<br>**ICD-10**: F50.0-50.1
**Bulimia nervosa**	Characterised by recurrent episodes of binge eating and inappropriate compensatory behaviour to prevent weight gain. These must occur, on average, at least twice a week for three months.	**DSM-IV-TR**: 307.51<br>**ICD-10**: F50.2
**Conduct disorder**	Occurs in those under 18 years of age and is characterised by a pattern of antisocial behaviour that violates the basic rights of others or major age-appropriate societal norms.	**DSM-IV-TR**: 312.81-312.89<br>**ICD-10**: F91
**Attention-deficit hyperactivity disorder**	Characterised by persistent inattention and/or hyperactivity-impulsivity. Diagnosis requires six or more symptoms of inattention or hyperactivity-impulsivity to have persisted for at least six months in two or more settings, with at least some impairing symptoms being present prior to 7 years of age.	**DSM-IV-TR**: 314.0, 314.01<br>**ICD-10**: F90
**Autism Spectrum Disorder**	Characterised by pervasive impairment in several areas of development, including social interaction and communication skills, along with restricted and repetitive patterns of behaviours and/or interests. Symptoms must be present in the early developmental period, cause clinically significant impairment, and not be better explained by intellectual impairment or global developmental delay.	**DSM-IV-TR**: 299.00, 299.80, 299.8, 299.8, 299.10<br>**ICD-10**: F84.0, F84.1, F84.2, F84.3, F84.4, F84.5, F84.8, F84.9
**Other mental disorders**	A residual cause within GBD incorporates disability from an aggregate group of personality disorders. Personality disorders are characterised by pervasive, inflexible and maladaptive patterns of behaviour and inner experience, which are markedly different from what is considered to be acceptable in the individual's culture.	**DSM-IV-TR**: 300.3, 301.0; 301.2, 301.22, 301.5–301.9<br>**ICD-10**: F60
**Idiopathic developmental intellectual disability**	Idiopathic developmental intellectual disability modelled as part of the intellectual disability impairment envelope in GBD 2019. Idiopathic developmental intellectual disability arises from any unknown source after the prevalence of all other sources of Intellectual Disability is accounted for.	**Includes idiopathic intellectual disability of all levels of severity (based on IQ score)**: borderline (IQ score 70-85), mild (IQ score 50-69), moderate (IQ score 35-49), severe (IQ score 20-34), and profound (IQ score 0-19)

Data selection considerations

Data sources considered for mental disorders are primarily population-representative surveys based on diagnostic interviews, or using symptom scales with a downward adjustment towards diagnostic prevalence. This methodology minimises data bias resulting from changes in treatment seeking behaviours. However, diagnostic interviews can vary across geography and time and are subject to changes in stigma and mental health awareness. Unfortunately, there is no straightforward approach to validate and quantify such biases.

For the most of mental disorders, data sources that rely on passive case finding, such as registry or surveillance studies, were excluded due to their susceptibility to auxiliary factors that may confound the interpretation of trends. The only disorder for which registry data were included is schizophrenia, as most of the cases are detected due to the severity of this disorder.

To ensure geographical and temporal comparability, GBD adopts standard case definitions as described in the main text and in appendix p. 13. Most of the data included in the analysis follow DSM-IV or ICD-10 diagnostic criteria, as these criteria have been deployed for decades, and the transition to new criteria, DSM-5 or ICD-11, have been slow. In some instances, older studies were included in which DSM-III was used, but the volume of data is limited. The consistency in definition reduces the likelihood of artifacts in trend changes caused by shifts in clinical taxonomy.

Statistical analysis

Disease model—Bayesian meta-regression (DisMod-MR) 2.1

DisMod-MR 2.1 involves the application of mixed-effects models and compartmental modelling concepts to synthesise diverse data sources and produce comprehensive estimates of prevalence, incidence, remission, and excess mortality (in the case of anorexia nervosa; see below) across age, sex, location, and over time. Separate models were developed for each cause of mental disorders. Data gaps were addressed through three key features in the disease modelling process: (1) the use of covariates, (2) the incorporation of spatiotemporal relationships, and (3) the structural framework imposed by the compartmental model. Regarding covariates, relevant predictors were selected for each mental disorder based on established knowledge from the literature. Examples include the Gallup Negative Experience Index, exposure to childhood sexual abuse, and intimate partner violence. The analysis also incorporated spatial associations captured by the GBD geographical hierarchy reflecting general epidemiological patterns across ASEAN regions and countries. Temporal associations were integrated into the covariance structure of the models. Finally, the compartmental model, which necessitates internal consistency across metrics for convergence, allows for the interpolation of missing metrics in a systematic and coherent manner.

COVID-19 adjustment

A specific adjustment was made to account for the impact of COVID-19, particularly for major depressive disorder and anxiety disorders. A systematic literature review was conducted to identify all relevant studies reporting prevalence during the pandemic period, a two-step modelling process was implemented to determine an adjustment factor. In the first step, a COVID-19 impact indicator was estimated using a meta-regression model to quantify the relative scale of the pandemic's impact. In the second step, this indicator, along with other covariates, was used to develop a model predicting daily prevalence changes in 2020 and 2021. The estimated change was then applied to the unadjusted prevalence estimates to obtain daily prevalence, which was subsequently averaged to calculate the annual point prevalence for these years. Details on the adjustment process can be found in GBD 2021 online methods appendices.[1,2]

Supplementary results

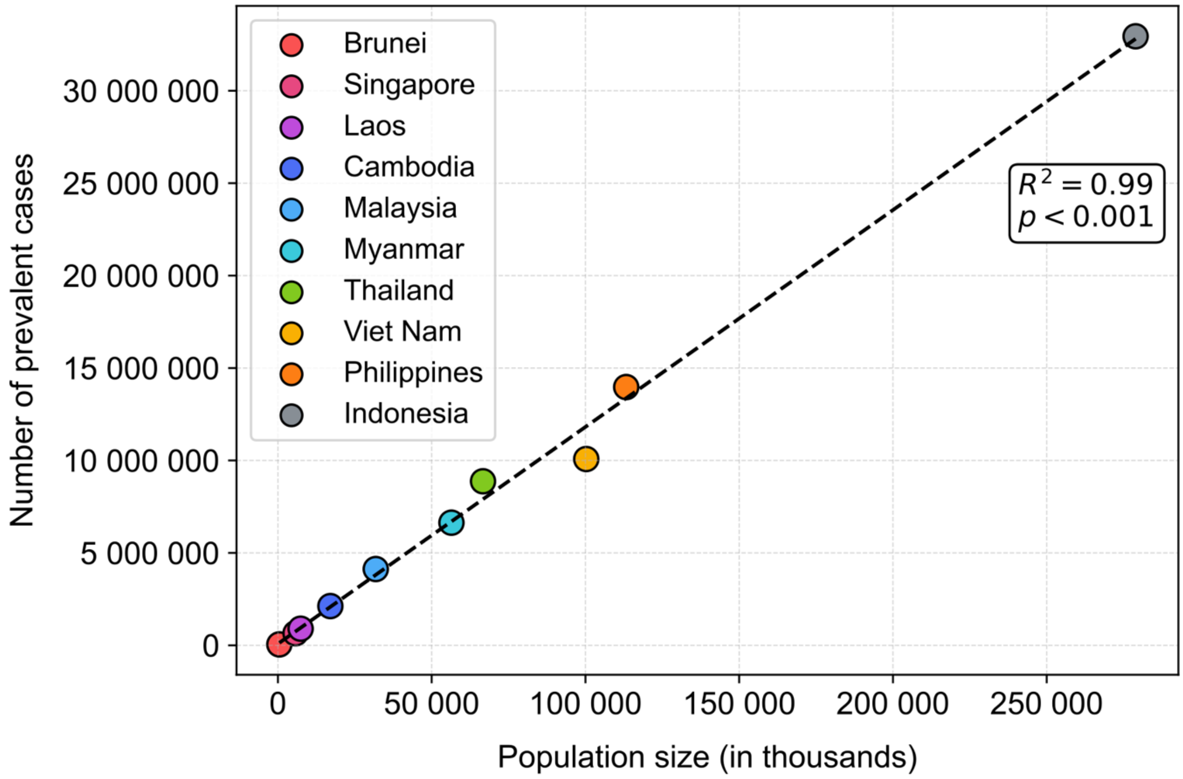

Figure S2 | Number of prevalent cases of mental disorders as a function of the population size (in thousands) for each ASEAN member state, 2021. ASEAN= Association of Southeast Asian Nations.

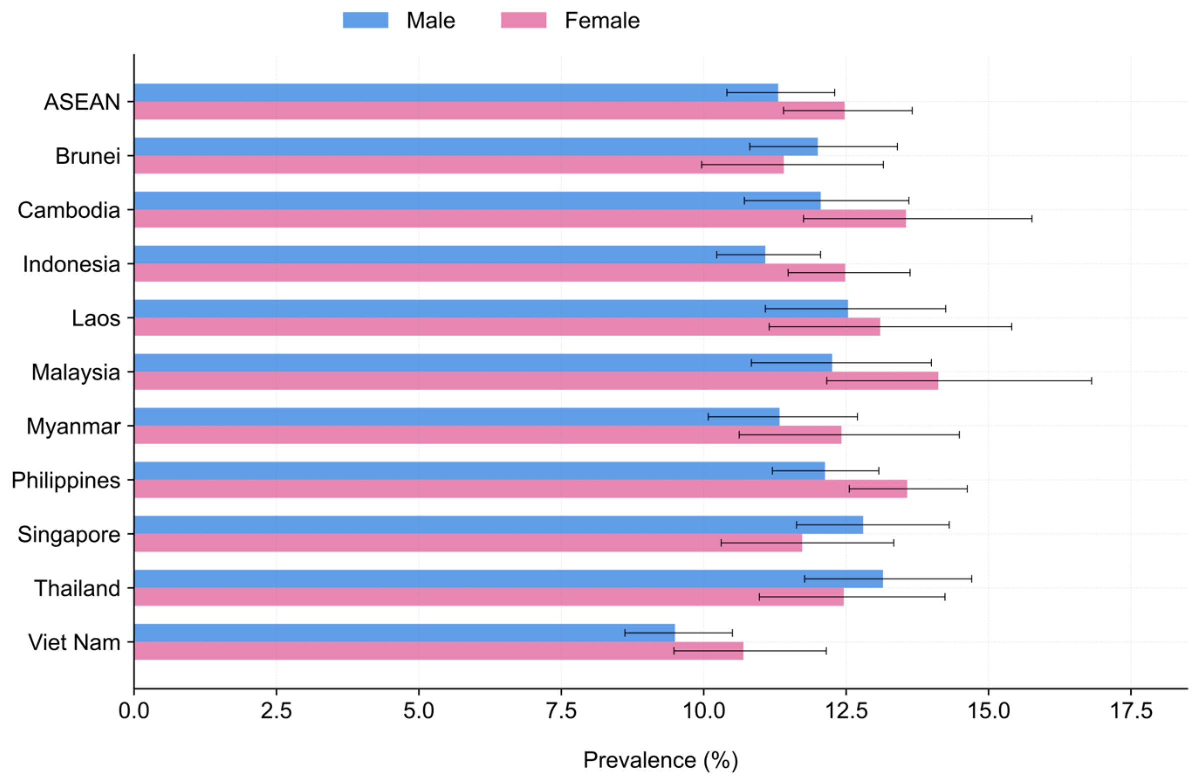

Figure S3 | Age-standardised prevalence (in %) of mental disorders in 2021 for ASEAN and its member states. The error bars represent the 95% uncertainty interval. ASEAN=Association of Southeast Asian Nations.

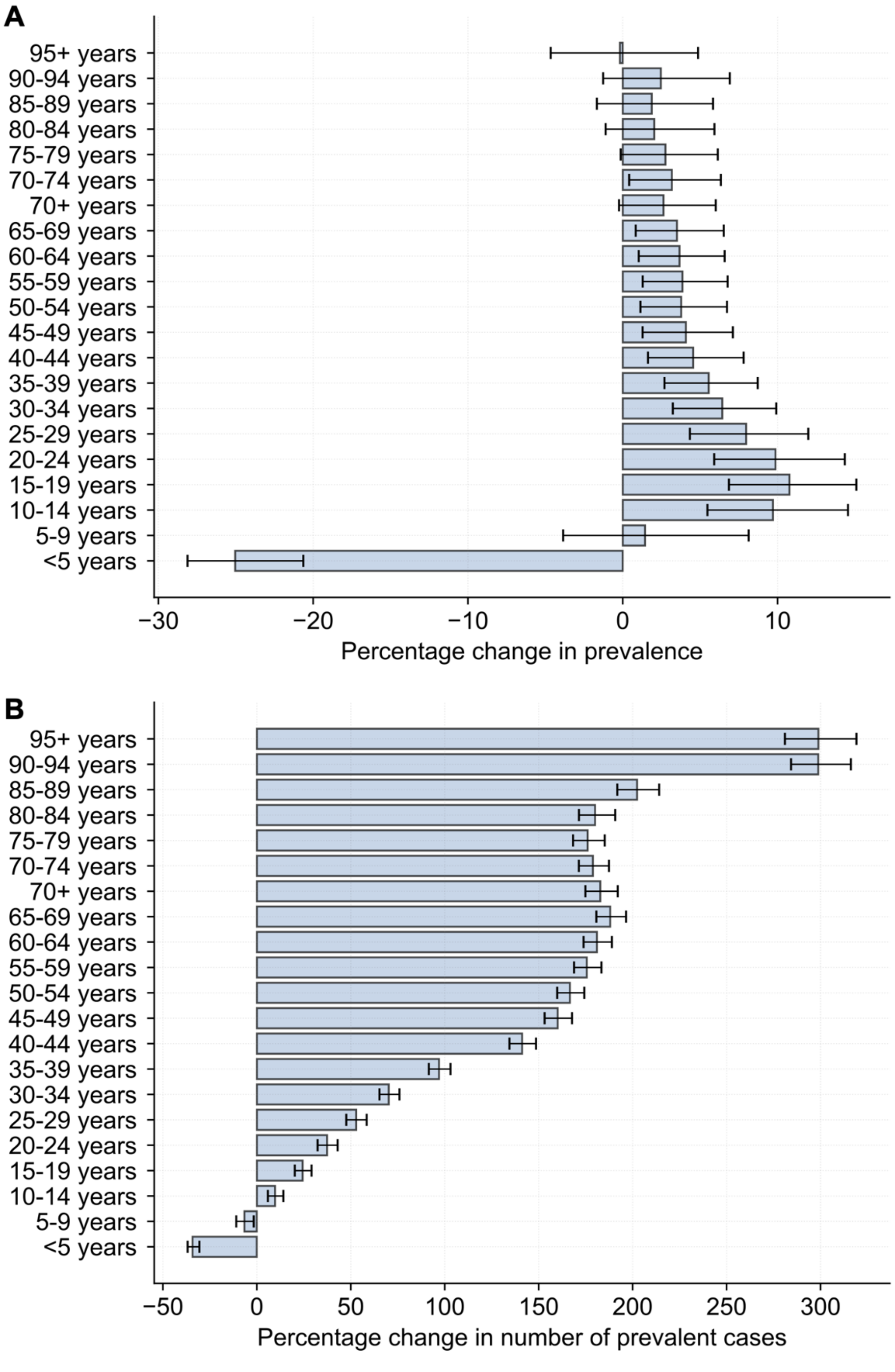

Figure S4 | Panel A: Percentage change in prevalence of mental disorders from 1990 to 2021 in ASEAN. **Panel B:** Percentage change in the number of prevalent cases of mental disorders from 1990 to 2021 in ASEAN. The error bars represent the 95% uncertainty interval. ASEAN=Association of Southeast Asian Nations.

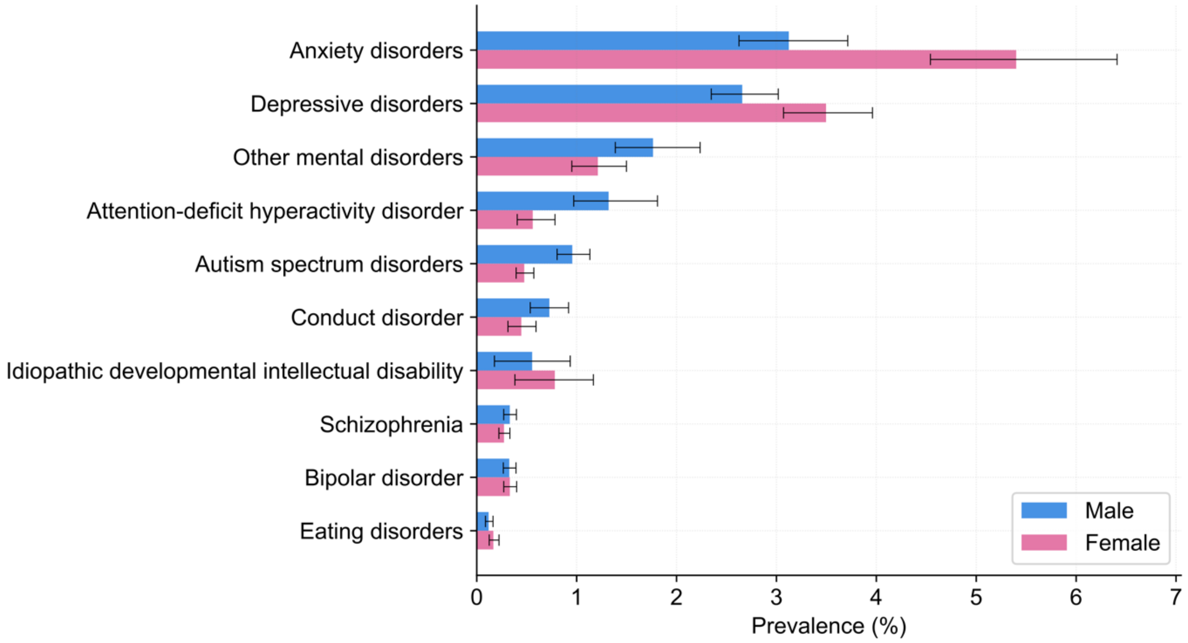

Figure S5 | Age-standardised prevalence of mental disorders in ASEAN, 2021, by sex and condition. The error bars represent the 95% uncertainty interval. ASEAN=Association of Southeast Asian Nations.

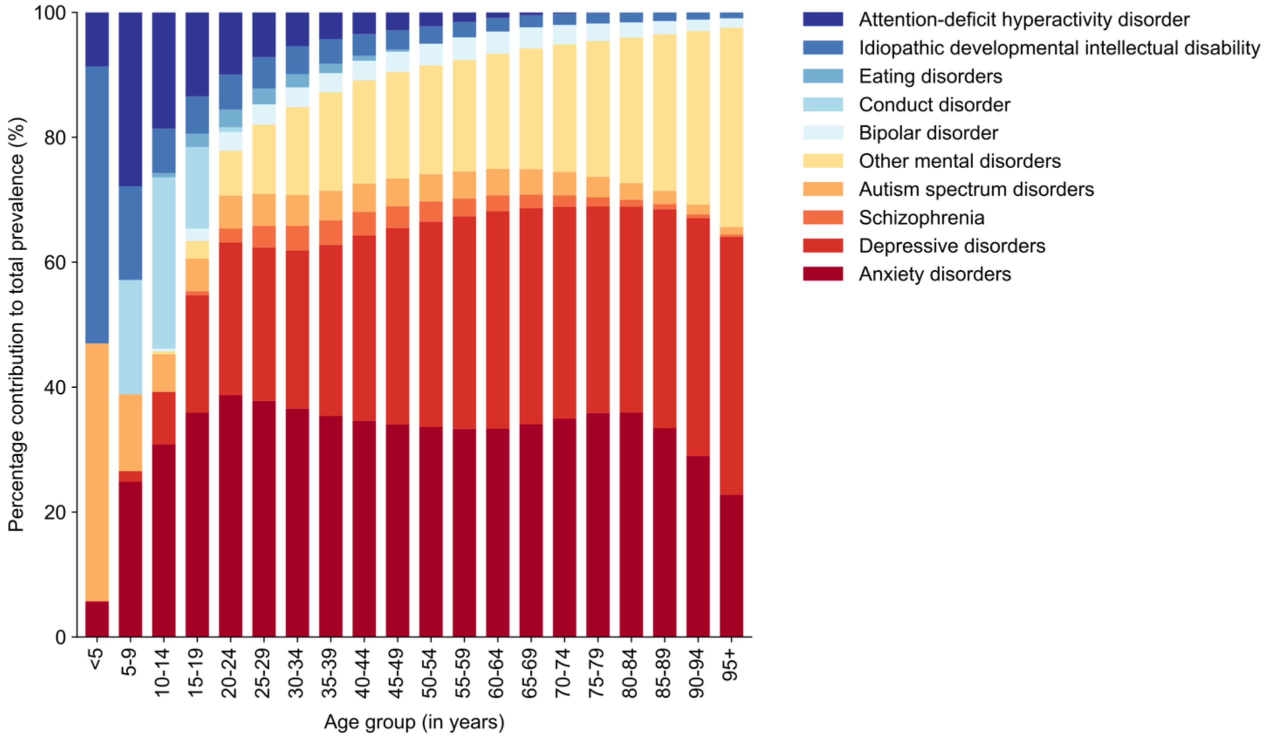

Figure S6 | Relative contribution (%) of Level 3 mental disorders to the total prevalence of mental disorders by age group in ASEAN, 2021. ASEAN=Association of Southeast Asian Nations.

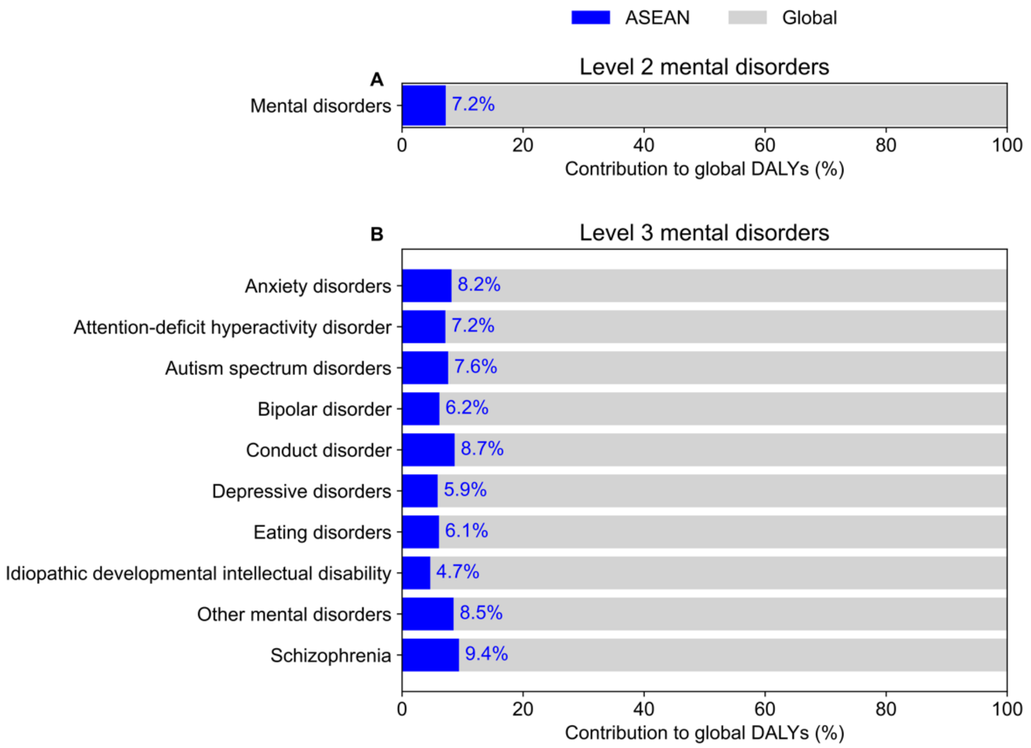

Figure S7 | Contribution of ASEAN DALYs to global DALYs (%) for **(A)** Level 2 mental disorders and (**B**) Level 3 mental disorders, 2021. DALYs=disability-adjusted life-years. ASEAN=Association of Southeast Asian Nations.

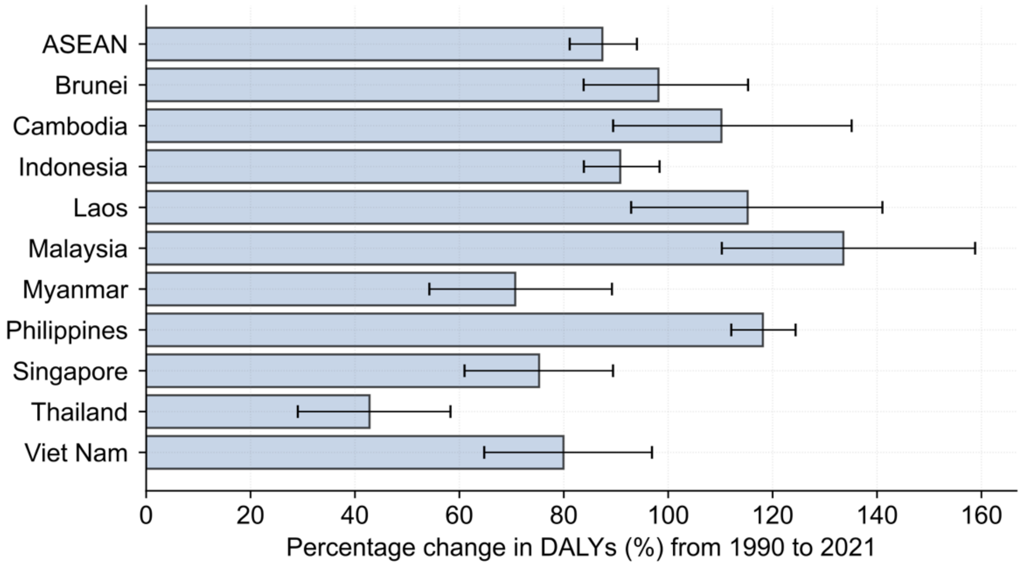

Figure S8 | Percentage change in DALYs (%) attributable to mental disorders from 1990 to 2021. The error bars represent the 95% uncertainty interval. DALYs=disability-adjusted life-years. DALYs=disability-adjusted life-years. ASEAN=Association of Southeast Asian Nations.

Table S2 | Sex- and age-disaggregated number of prevalent cases (in thousands) and prevalence (in %) of mental disorders in 1990, 2019, and 2021, along with the percentage change (in %) from 1990 to 2021 in the ASEAN region. ASEAN=Association of Southeast Asian Nations. The 2019 prevalence data serve as a reference for pre-COVID levels.

	1990		2019		2021		Percentage increase from 1990 to 2021	
	Number of prevalent cases, in thousands (95% UI)	Prevalence (95% UI)	Number of prevalent cases, in thousands (95% UI)	Prevalence (95% UI)	Number of prevalent cases, in thousands (95% UI)	Prevalence (95% UI)	Number of prevalent cases (95% UI)	Prevalence (95% UI)
**<5 years**								
Total	1 519	2.8	1 015	2.1	999	2.1	-34.3	-25.0
	(1 081–1 947)	(2.0–3.6)	(743–1 299)	(1.5–2.7)	(725–1 269)	(1.5–2.7)	(-36.8–-30.6)	(-28.1–-20.6)
Male	782	2.8	559	2.3	548	2.3	-29.9	-19.5
	(562–997)	(2.0–3.6)	(419–704)	(1.7–2.9)	(411–685)	(1.7–2.9)	(-33.2–-23.9)	(-23.3–-13.0)
Female	738	2.8	456	1.9	451	1.9	-38.9	-30.8
	(516–952)	(2.0–3.6)	(320–592)	(1.3–2.5)	(315–583)	(1.4–2.5)	(-41.5–-36.2)	(-33.9–-27.3)
**5-9 years**								
Total	3 565	6.5	3 126	6.2	3 330	6.6	-6.6	1.4
	(2 950–4 286)	(5.4–7.8)	(2 545–3 755)	(5.0–7.4)	(2 737–3 980)	(5.4–7.9)	(-10.9–-1.6)	(-3.9–8.1)
Male	2 012	7.2	1 817	7.1	1 884	7.4	-6.4	2.3
	(1 654–2 419)	(5.9–8.7)	(1 487–2 189)	(5.8–8.6)	(1 535–2 268)	(6.0–9.0)	(-10.5–-2.2)	(-3.2–8.1)
Female	1 554	5.8	1 309	5.2	1 446	5.8	-6.9	0.4
	(1 259–1 843)	(4.7–6.9)	(1 074–1 571)	(4.3–6.3)	(1 192–1 737)	(4.8–7.0)	(-12.1–-0.6)	(-5.8–8.4)
**10-14 years**								
Total	5 992	11.5	5 921	11.5	6 575	12.7	9.7	9.7
	(5 067–6 984)	(9.8–13.4)	(4 993–6 916)	(9.7–13.3)	(5 583–7 630)	(10.8–14.7)	(5.9–14.1)	(5.5–14.6)
Male	3 249	12.3	3 249	12.6	3 496	13.4	7.6	8.8
	(2 759–3 771)	(10.5–14.3)	(2 743–3 777)	(10.7–14.6)	(2 987–4 084)	(11.6–15.5)	(4.2–11.4)	(4.5–13.3)
Female	2 744	10.7	2 672	10.4	3 079	11.9	12.2	10.9
	(2 302–3 214)	(9.0–12.6)	(2 238–3 154)	(8.8–12.3)	(2 571–3 626)	(10.0–14.1)	(7.2–17.5)	(5.9–16.3)
**15-19 years**								
Total	5 755	12.2	6 302	11.9	7 158	13.6	24.4	10.8
	(5 035–6 496)	(10.7–13.8)	(5 551–7 126)	(10.5–13.5)	(6 319–8 141)	(12.0–15.4)	(20.2–29.1)	(6.9–15.1)
Male	2 879	12.4	3 236	12.2	3 566	13.4	23.9	8.8
	(2 506–3 221)	(10.8–13.8)	(2 844–3 624)	(10.7–13.8)	(3 139–4 009)	(11.9–15.2)	(20.0–28.3)	(4.9–13.0)
Female	2 876	12.1	3 066	11.7	3 591	13.7	24.9	12.8
	(2 516–3 299)	(10.6–13.9)	(2 665–3 507)	(10.2–13.3)	(3 102–4 144)	(11.9–15.8)	(20.1–30.3)	(8.4–17.7)
**20-24 years**								
Total	5 124	12.0	6 128	11.5	7 041	13.2	37.4	9.9
	(4 442–5 879)	(10.4–13.8)	(5 331–7 074)	(10.0–13.2)	(6 045–8 158)	(11.3–15.3)	(32.3–43.0)	(5.9–14.3)
Male	2 383	11.5	2 952	11.0	3 323	12.4	39.4	7.9
	(2 075–2 725)	(10.0–13.1)	(2 591–3 376)	(9.7–12.6)	(2 894–3 816)	(10.8–14.3)	(34.1–44.7)	(3.7–12.1)
Female	2 741	12.5	3 175	11.9	3 718	14.0	35.7	12.0
	(2 350–3 174)	(10.7–14.4)	(2 702–3 725)	(10.1–13.9)	(3 140–4 390)	(11.8–16.5)	(30.0–41.8)	(7.4–17.1)
**25-29 years**								
Total	4 792	12.4	6 448	11.9	7 325	13.4	52.9	8.0
	(4 196–5 508)	(10.9–14.3)	(5 685–7 386)	(10.5–13.6)	(6 413–8 475)	(11.7–15.5)	(47.6–58.5)	(4.3–12.0)
Male	2 171	11.7	3 045	11.2	3 397	12.4	56.5	6.3

	(1 904–2 456)	(10.2–13.2)	(2 689–3 429)	(9.9–12.6)	(2 992–3 871)	(10.9–14.2)	(51.6–61.5)	(2.9–9.7)
Female	2 621	13.1	3 403	12.6	3 928	14.5	49.9	10.0
	(2 274–3 052)	(11.4–15.3)	(2 947–3 972)	(10.9–14.7)	(3 396–4 617)	(12.5–17.0)	(44.1–56.4)	(5.6–14.9)
**30-34 years**								
Total	4 312	12.9	6 464	12.4	7 340	13.7	70.2	6.4
	(3 834–4 847)	(11.5–14.5)	(5 760–7 266)	(11.0–13.9)	(6 537–8 230)	(12.2–15.4)	(65.3–75.9)	(3.2–9.9)
Male	1 971	12.0	3 039	11.6	3 392	12.6	72.1	4.8
	(1 757–2 193)	(10.7–13.4)	(2 717–3 391)	(10.4–13.0)	(3 044–3 809)	(11.3–14.2)	(67.6–77.0)	(2.0–8.0)
Female	2 342	13.8	3 425	13.2	3 948	14.9	68.6	8.2
	(2 060–2 646)	(12.1–15.5)	(3 015–3 866)	(11.6–14.9)	(3 483–4 497)	(13.1–16.9)	(63.0–75.1)	(4.6–12.4)
**35-39 years**								
Total	3 639	13.4	6 448	12.9	7 169	14.1	97.0	5.6
	(3 276–4 065)	(12.1–15.0)	(5 814–7 216)	(11.7–14.4)	(6 452–8 007)	(12.7–15.8)	(91.5–103.1)	(2.7–8.7)
Male	1 663	12.4	3 005	12.0	3 282	12.9	97.3	4.2
	(1 495–1 856)	(11.1–13.8)	(2 716–3 345)	(10.8–13.3)	(2 950–3 652)	(11.5–14.3)	(92.5–102.8)	(1.6–7.1)
Female	1 976	14.4	3 442	13.9	3 887	15.4	96.7	7.0
	(1 767–2 230)	(12.9–16.3)	(3 073–3 857)	(12.4–15.5)	(3 458–4 383)	(13.7–17.4)	(90.4–103.5)	(3.5–10.6)
**40-44 years**								
Total	2 846	13.8	6 112	13.3	6 866	14.4	141.2	4.6
	(2 518–3 213)	(12.2–15.6)	(5 413–6 908)	(11.8–15.0)	(6 086–7 777)	(12.8–16.3)	(134.5–148.6)	(1.6–7.8)
Male	1 289	12.6	2 809	12.2	3 111	13.0	141.3	3.3
	(1 153–1 452)	(11.3–14.2)	(2 505–3 172)	(10.9–13.8)	(2 757–3 502)	(11.5–14.7)	(135.4–148.0)	(0.7–6.2)
Female	1 557	14.9	3 303	14.3	3 754	15.8	141.2	5.8
	(1 371–1 769)	(13.2–17.0)	(2 912–3 749)	(12.6–16.3)	(3 292–4 271)	(13.8–18.0)	(133.2–149.5)	(2.3–9.4)
**45-49 years**								
Total	2 394	13.9	5 654	13.4	6 228	14.4	160.2	4.1
	(2 133–2 702)	(12.4–15.7)	(5 007–6 379)	(11.9–15.1)	(5 523–6 994)	(12.8–16.2)	(153.2–167.8)	(1.3–7.1)
Male	1 063	12.7	2 585	12.3	2 804	13.1	163.7	3.1
	(951–1 197)	(11.3–14.3)	(2 315–2 903)	(11.0–13.8)	(2 494–3 149)	(11.6–14.7)	(157.7–170.9)	(0.8–6.0)
Female	1 330	15.0	3 070	14.5	3 424	15.8	157.4	5.2
	(1 179–1 510)	(13.3–17.1)	(2 717–3 495)	(12.8–16.5)	(2 993–3 911)	(13.8–18.1)	(148.9–166.2)	(1.7–8.8)
**50-54 years**								
Total	2 097	13.8	5 043	13.4	5 593	14.3	166.7	3.8
	(1 888–2 338)	(12.4–15.4)	(4 553–5 616)	(12.1–14.9)	(5 021–6 246)	(12.8–16.0)	(159.9–174.3)	(1.1–6.7)
Male	925	12.6	2 286	12.3	2 505	13.0	170.8	3.1
	(838–1 018)	(11.4–13.9)	(2 072–2 540)	(11.2–13.7)	(2 279–2 775)	(11.8–14.4)	(164.7–178.0)	(0.8–5.9)
Female	1 172	14.9	2 757	14.4	3 088	15.6	163.4	4.6
	(1 055–1 321)	(13.4–16.8)	(2 469–3 106)	(12.9–16.2)	(2 754–3 457)	(13.9–17.4)	(155.5–172.4)	(1.5–8.2)
**55-59 years**								
Total	1 725	13.6	4 273	13.3	4 755	14.1	175.6	3.9
	(1 568–1 913)	(12.3–15.0)	(3 893–4 753)	(12.1–14.8)	(4 314–5 277)	(12.8–15.6)	(168.8–183.4)	(1.3–6.8)
Male	757	12.4	1 923	12.3	2 111	12.8	178.8	3.6
	(686–841)	(11.2–13.8)	(1 742–2 130)	(11.1–13.6)	(1 916–2 331)	(11.7–14.2)	(172.3–186.2)	(1.2–6.4)
Female	968	14.6	2 350	14.2	2 644	15.2	173.2	4.3
	(874–1 078)	(13.2–16.3)	(2 127–2 632)	(12.9–16.0)	(2 378–2 963)	(13.7–17.1)	(165.1–182.0)	(1.2–7.6)
**60-64 years**								
Total	1 349	13.2	3 339	13.1	3 791	13.7	181.0	3.7
	(1 199–1 535)	(11.8–15.1)	(2 967–3 807)	(11.6–14.9)	(3 366–4 325)	(12.2–15.7)	(173.9–189.0)	(1.0–6.6)

Male	594	12.2	1 485	12.1	1 667	12.6	180.6	3.5
	(525–666)	(10.8–13.6)	(1 323–1 667)	(10.8–13.6)	(1 485–1 872)	(11.2–14.1)	(173.6–188.4)	(1.0–6.4)
Female	755	14.2	1 855	13.9	2 124	14.8	181.4	3.8
	(665–863)	(12.5–16.3)	(1 629–2 134)	(12.3–16.0)	(1 874–2 448)	(13.0–17.0)	(172.5–190.9)	(0.5–7.3)
**65-69 years**								
Total	930	12.7	2 317	12.7	2 680	13.2	188.1	3.5
	(821–1 060)	(11.3–14.5)	(2 032–2 662)	(11.1–14.6)	(2 365–3 088)	(11.6–15.2)	(180.7–196.5)	(0.8–6.5)
Male	401	11.7	1 010	11.8	1 152	12.1	187.0	3.4
	(357–454)	(10.4–13.3)	(900–1 142)	(10.5–13.3)	(1 023–1 303)	(10.8–13.7)	(179.8–194.5)	(0.8–6.1)
Female	529	13.6	1 307	13.5	1 528	14.1	189.0	3.6
	(461–608)	(11.9–15.7)	(1 132–1 516)	(11.7–15.6)	(1 330–1 774)	(12.3–16.4)	(180.1–199.1)	(0.4–7.2)
**70-74 years**								
Total	590	12.3	1 437	12.2	1 645	12.6	178.9	3.2
	(523–670)	(10.9–13.9)	(1 269–1 642)	(10.8–14.0)	(1 454–1 879)	(11.2–14.4)	(171.4–187.4)	(0.4–6.3)
Male	246	11.3	603	11.3	688	11.6	179.4	2.9
	(221–277)	(10.1–12.7)	(540–676)	(10.1–12.7)	(615–774)	(10.4–13.1)	(172.8–186.9)	(0.5–5.7)
Female	344	13.1	834	13.0	957	13.5	178.5	3.4
	(300–397)	(11.4–15.1)	(725–967)	(11.3–15.1)	(834–1 114)	(11.8–15.7)	(169.8–188.8)	(0.2–7.2)
**75-79 years**								
Total	345	11.7	871	11.7	952	12.1	176.1	2.8
	(307–389)	(10.4–13.3)	(774–992)	(10.4–13.4)	(844–1 081)	(10.7–13.7)	(168.3–185.2)	(-0.1–6.1)
Male	138	10.8	343	10.8	373	11.1	170.0	2.2
	(124–155)	(9.7–12.1)	(308–383)	(9.7–12.1)	(333–417)	(9.9–12.4)	(162.8–177.9)	(-0.5–5.2)
Female	207	12.5	528	12.4	579	12.8	180.1	2.9
	(182–237)	(11.0–14.3)	(463–611)	(10.9–14.4)	(506–671)	(11.2–14.9)	(170.9–190.5)	(-0.4–6.8)
**80-84 years**								
Total	193	11.4	514	11.4	541	11.6	180.1	2.0
	(171–218)	(10.1–12.9)	(452–579)	(10.1–12.9)	(473–612)	(10.2–13.2)	(171.4–190.7)	(-1.1–5.9)
Male	74.4	10.5	186	10.5	195	10.6	161.5	1.0
	(66.0–83.6)	(9.3–11.8)	(165–210)	(9.3–11.8)	(172–219)	(9.4–11.9)	(153.3–170.8)	(-2.1–4.6)
Female	119	12.1	327	12.1	346	12.3	191.7	2.1
	(104–135)	(10.6–13.7)	(286–372)	(10.6–13.7)	(299–395)	(10.6–14.1)	(181.5–204.1)	(-1.5–6.4)
**85-89 years**								
Total	85.2	11.2	242	11.3	258	11.5	202.4	1.9
	(75.5–96.0)	(10.0–12.7)	(213–275)	(10.0–12.9)	(225–295)	(10.0–13.1)	(191.9–214.1)	(-1.7–5.8)
Male	31.5	10.3	81.3	10.3	85.7	10.3	172.3	0.4
	(27.8–35.2)	(9.1–11.5)	(71.7–91.9)	(9.1–11.6)	(75.4–97.7)	(9.1–11.8)	(162.4–183.3)	(-3.2–4.5)
Female	53.8	11.9	161	12.0	172	12.1	220.0	1.8
	(47.2–61.3)	(10.4–13.6)	(140–185)	(10.4–13.7)	(149–199)	(10.5–14.0)	(207.9–233.2)	(-2.0–6.1)
**90-94 years**								
Total	23.3	11.1	86.4	11.4	93.0	11.4	298.8	2.5
	(20.3–26.9)	(9.7–12.8)	(74.9–99.9)	(9.9–13.2)	(81.0–108)	(9.9–13.3)	(284.3–316.2)	(-1.3–6.9)
Male	7.04	10.0	28.6	10.4	30.1	10.3	328.1	3.1
	(6.18–8.05)	(8.8–11.5)	(25.0–32.7)	(9.1–11.9)	(26.2–34.4)	(9.0–11.8)	(312.7–345.6)	(-0.6–7.3)
Female	16.3	11.7	57.8	12.0	62.9	12.0	286.2	2.7
	(14.1–18.9)	(10.2–13.6)	(49.7–67.1)	(10.3–13.9)	(54.2–73.9)	(10.3–14.1)	(269.9–305.6)	(-1.7–7.8)
**95+ years**								
Total	6.77	11.5	24.2	11.6	27.0	11.5	298.9	-0.2

	(5.73–7.97)	(9.7–13.6)	(20.5–28.4)	(9.8–13.6)	(22.8–31.7)	(9.7–13.5)	(281.1–319.1)	(-4.7–4.9)
Male	1.45	10.3	8.47	10.7	9.32	10.6	542.2	2.5
	(1.23–1.69)	(8.8–12.1)	(7.14–9.95)	(9.0–12.6)	(7.84–10.9)	(8.9–12.4)	(516.8–571.5)	(-1.6–7.1)
Female	5.31	11.9	15.8	12.1	17.7	12.0	232.5	1.2
	(4.47–6.26)	(10.0–14.0)	(13.2–18.8)	(10.1–14.3)	(14.8–20.9)	(10.0–14.2)	(215.0–251.8)	(-4.1–7.1)

95% UI=95% uncertainty interval.

Table S3 | ASEAN number of prevalent cases (95% UI) (in thousands) of mental disorders in 2021 by disorder, sex, and country. ASEAN=Association of Southeast Asian Nations.

	Number of prevalent cases in thousands (95% UI) in 2021									
	**Anxiety disorders**	**Attention-deficit hyperactivity disorder**	**Autism spectrum disorders**	**Bipolar disorder**	**Conduct disorder**	**Depressive disorders**	**Eating disorders**	**Idiopathic developmental intellectual disability**	**Other mental disorders**	**Schizophrenia**
**ASEAN**										
Total	29 062	6 069	4 679	2 291	3 558	21 296	975	4 297	10 262	2 148
	(24 453–34 467)	(4 485–8 298)	(3 935–5 548)	(1 859–2 767)	(2 585–4 542)	(18 793–24 132)	(718–1 319)	(1 799–6 781)	(8 011–12 882)	(1 744–2 594)
Male	10 502	4 283	3 117	1 117	2 236	8 977	409	1 788	5 961	1 164
	(8 810–12 521)	(3 156–5 855)	(2 629–3 679)	(902–1 350)	(1 645–2 818)	(7 906–10 254)	(301–562)	(566–3 027)	(4 669–7 559)	(948–1 402)
Female	18 560	1 786	1 562	1 175	1 321	12 319	566	2 509	4 300	984
	(15 610–22 095)	(1 296–2 475)	(1 296–1 876)	(956–1 421)	(924–1 755)	(10 814–14 006)	(423–757)	(1 221–3 752)	(3 368–5 325)	(799–1 192)
**Brunei**										
Total	15.9	5.61	6.27	2.60	2.09	9.17	2.64	0.12	8.18	1.39
	(11.2–22.0)	(4.07–7.73)	(5.28–7.47)	(1.95–3.40)	(1.50–2.70)	(7.51–11.3)	(2.00–3.45)	(0.00–0.67)	(6.43–10.4)	(1.10–1.76)
Male	6.19	4.33	4.47	1.24	1.39	3.86	1.02	0.09	5.17	0.72
	(4.26–8.54)	(3.16–6.01)	(3.71–5.31)	(0.93–1.64)	(1.03–1.78)	(3.14–4.72)	(0.74–1.38)	(0.00–0.51)	(4.02–6.57)	(0.55–0.92)
Female	9.69	1.27	1.80	1.36	0.70	5.31	1.62	0.02	3.01	0.68
	(6.84–13.4)	(0.91–1.79)	(1.48–2.15)	(1.02–1.77)	(0.48–0.95)	(4.29–6.64)	(1.23–2.12)	(0.00–0.17)	(2.39–3.87)	(0.53–0.86)
**Cambodia**										
Total	796	152	117	51.8	97.6	561	19.5	169	236	45.8
	(564–1 084)	(112–208)	(97.6–140)	(38.7–67.6)	(69.9–127)	(450–674)	(14.4–26.6)	(89.3–257)	(184–301)	(34.6–58.7)
Male	276	106	78.3	24.5	62.4	237	8.13	70.4	133	24.3
	(196–381)	(77.6–148)	(64.7–93.8)	(18.0–32.3)	(45.2–79.6)	(189–286)	(5.78–11.4)	(31.0–113)	(105–171)	(18.2–31.1)
Female	519	46.0	38.8	27.3	35.2	324	11.4	98.2	102	21.5
	(366–708)	(32.7–66.3)	(31.8–46.7)	(20.5–35.6)	(24.2–47.8)	(261–389)	(8.43–15.3)	(55.3–144)	(79.8–131)	(16.4–27.7)
**Indonesia**										
Total	12 390	2 385	1 820	950	1 506	8 347	410	1 866	4 116	888
	(10 586–14 627)	(1 713–3 269)	(1 529–2 163)	(799–1 112)	(1 096–1 910)	(7 397–9 410)	(301–552)	(802–2 871)	(3 207–5 134)	(737–1 054)
Male	4 577	1 682	1 157	472	938	3 498	174	801	2 419	492
	(3 899–5 425)	(1 212–2 305)	(975–1 368)	(397–554)	(695–1 179)	(3 101–3 935)	(126–239)	(269–1 313)	(1 880–3 058)	(409–581)
Female	7 813	703	663	478	568	4 849	236	1 065	1 697	396
	(6 685–9 196)	(501–972)	(551–793)	(402–560)	(396–750)	(4 271–5 497)	(176–316)	(533–1 569)	(1 323–2 108)	(329–469)
**Laos**										
Total	366	67.0	50.7	22.6	42.8	212	10.2	60.9	99.1	19.9
	(258–501)	(49.2–91.8)	(42.3–60.8)	(16.8–29.3)	(30.7–55.5)	(171–262)	(7.49–13.9)	(29.3–92.2)	(77.2–127)	(15.2–25.0)
Male	143	46.7	34.2	11.2	27.2	103	4.24	24.7	58.6	10.9
	(101–198)	(34.2–65.3)	(28.5–40.9)	(8.21–14.5)	(19.8–34.6)	(81.2–128)	(3.06–5.89)	(8.46–40.1)	(46.1–75.3)	(8.42–13.8)
Female	223	20.3	16.4	11.4	15.6	109	5.95	36.2	40.4	8.94
	(157–307)	(14.5–29.3)	(13.5–20.0)	(8.50–14.8)	(10.8–21.2)	(86.7–134)	(4.43–8.02)	(20.2–52.2)	(31.2–52.0)	(6.75–11.5)
**Malaysia**										
Total	1 738	126	235	106	157	1 224	64.7	106	499	106
	(1 237–2 389)	(89.1–176)	(196–280)	(80.2–138)	(112–203)	(1 003–1 513)	(47.4–88.5)	(21.3–193)	(390–634)	(81.3–136)
Male	651	85.1	163	54.0	101	571	27.8	44.6	304	59.6
	(463–910)	(61.6–117)	(135–194)	(40.4–70.1)	(73.3–128)	(460–709)	(20.3–38.7)	(4.47–88.8)	(239–388)	(45.9–76.7)

Female	1 087	40.5	72.2	52.4	56.4	653	36.9	61.1	195	46.2
	(779–1 497)	(27.0–58.4)	(59.1–87.0)	(39.4–67.1)	(38.8–76.4)	(532–804)	(27.1–49.9)	(17.5–102)	(152–250)	(35.0–60.1)
**Myanmar**										
Total	2 655	482	381	177	310	1 491	71.1	439	819	156
	(1 905–3 523)	(355–660)	(316–457)	(134–224)	(222–403)	(1 225–1 808)	(53.0–96.1)	(194–689)	(640–1 042)	(121–196)
Male	907	332	252	82.2	196	640	28.4	178	457	80.8
	(646–1 256)	(243–463)	(209–303)	(61.0–105)	(143–250)	(523–778)	(20.7–39.3)	(59.9–295)	(361–585)	(61.8–102)
Female	1 748	150	129	95.1	114	851	42.7	261	362	74.8
	(1 221–2 303)	(107–216)	(106–156)	(71.9–121)	(78.6–155)	(697–1 050)	(31.9–57.6)	(136–393)	(283–462)	(58.3–94.1)
**Philippines**										
Total	5 402	1 066	792	349	739	3 710	159	762	1 453	320
	(4 630–6 262)	(764–1 463)	(665–936)	(296–410)	(537–938)	(3 284–4 211)	(117–216)	(290–1 226)	(1 130–1 809)	(263–380)
Male	1 971	753	539	172	460	1 662	68.1	334	851	175
	(1 686–2 284)	(536–1 037)	(454–634)	(145–202)	(340–577)	(1 463–1 899)	(49.6–93.9)	(89.4–573)	(664–1 078)	(143–209)
Female	3 431	312	253	177	279	2 047	90.7	428	602	145
	(2 951–3 985)	(222–436)	(210–303)	(150–208)	(195–369)	(1 812–2 324)	(67.5–122)	(199–654)	(468–740)	(120–171)
**Singapore**										
Total	185	50.6	83.5	40.1	15.3	144	26.2	0.59	123	19.5
	(131–251)	(37.1–69.0)	(70.1–99.2)	(31.5–50.3)	(11.0–19.8)	(118–174)	(19.6–34.2)	(0.00–5.01)	(98.1–155)	(15.2–24.4)
Male	67.9	38.0	59.2	18.3	9.89	62.4	9.97	0.43	75.0	9.09
	(48.7–92.8)	(27.7–52.2)	(49.6–70.2)	(14.6–22.8)	(7.34–12.7)	(51.2–75.4)	(7.14–13.5)	(0.00–3.59)	(59.3–94.9)	(7.04–11.5)
Female	117	12.6	24.3	21.7	5.45	81.1	16.2	0.16	47.8	10.4
	(83.1–161)	(9.14–17.5)	(19.9–29.2)	(16.8–27.7)	(3.74–7.44)	(66.2–99.1)	(12.1–21.4)	(0.00–1.46)	(38.1–60.7)	(8.16–13.1)
**Thailand**										
Total	2 836	941	464	254	221	2 663	90.0	392	1 289	235
	(2 001–3 850)	(705–1 284)	(384–556)	(199–320)	(158–286)	(2 212–3 155)	(66.2–123)	(137–650)	(1 020–1 626)	(186–293)
Male	982	677	311	119	141	1 103	35.6	249	724	122
	(688–1 366)	(498–919)	(258–375)	(92.4–151)	(102–179)	(909–1 318)	(26.2–49.7)	(90.4–410)	(574–923)	(97.1–150)
Female	1 854	264	153	135	80.0	1 560	54.4	143	565	113
	(1 291–2 525)	(197–365)	(126–185)	(107–170)	(55.1–108)	(1 277–1 855)	(39.8–73.8)	(49.0–237)	(443–714)	(88.5–143)
**Viet Nam**										
Total	2 677	795	729	337	466	2 935	122	503	1 619	357
	(1 920–3 586)	(590–1 088)	(608–867)	(258–429)	(334–607)	(2 423–3 523)	(91.3–162)	(227–812)	(1 266–2 053)	(278–442)
Male	921	559	518	162	301	1 095	51.4	86.9	933	190
	(659–1 253)	(412–778)	(428–616)	(122–211)	(217–384)	(894–1 318)	(37.7–69.9)	(8.54–197)	(735–1 190)	(146–236)
Female	1 756	236	210	175	166	1 840	70.3	417	686	167
	(1 245–2 402)	(169–335)	(172–253)	(133–224)	(114–225)	(1 526–2 216)	(52.6–94.3)	(219–618)	(533–871)	(131–209)

95% UI=95% uncertainty interval.

Table S4 | ASEAN age-standardised prevalence (95% UI) (in %) of mental disorders in 2021 by disorder, sex, and country. ASEAN=Association of Southeast Asian Nations.

	Age-standardised prevalence (95% UI) in 2021									
	Anxiety disorders	Attention-deficit hyperactivity disorder	Autism spectrum disorders	Bipolar disorder	Conduct disorder	Depressive disorders	Eating disorders	Idiopathic developmental intellectual disability	Other mental disorders	Schizophrenia
**ASEAN**										
Total	4.28	0.94	0.71	0.33	0.59	3.08	0.14	0.67	1.48	0.30
	(3.61–5.06)	(0.70–1.30)	(0.60–0.85)	(0.27–0.40)	(0.43–0.75)	(2.73–3.50)	(0.11–0.19)	(0.28–1.05)	(1.16–1.86)	(0.24–0.36)
Male	3.12	1.32	0.96	0.33	0.73	2.66	0.12	0.56	1.76	0.33
	(2.63–3.71)	(0.97–1.81)	(0.80–1.13)	(0.27–0.39)	(0.54–0.92)	(2.35–3.02)	(0.09–0.16)	(0.18–0.94)	(1.39–2.24)	(0.27–0.40)
Female	5.40	0.56	0.48	0.33	0.45	3.50	0.17	0.78	1.21	0.27
	(4.54–6.41)	(0.41–0.78)	(0.39–0.57)	(0.27–0.40)	(0.31–0.59)	(3.07–3.96)	(0.12–0.22)	(0.38–1.17)	(0.95–1.50)	(0.22–0.33)
**Brunei**										
Total	3.60	1.40	1.49	0.56	0.62	2.02	0.54	0.03	1.76	0.28
	(2.58–4.92)	(1.00–1.94)	(1.26–1.78)	(0.42–0.73)	(0.44–0.80)	(1.67–2.48)	(0.41–0.71)	(0.00–0.16)	(1.40–2.25)	(0.23–0.35)
Male	2.71	2.08	2.05	0.52	0.79	1.64	0.39	0.04	2.14	0.28
	(1.87–3.79)	(1.49–2.86)	(1.71–2.43)	(0.39–0.68)	(0.58–1.02)	(1.34–1.99)	(0.28–0.53)	(0.00–0.24)	(1.68–2.71)	(0.22–0.35)
Female	4.57	0.66	0.89	0.61	0.43	2.44	0.72	0.01	1.36	0.29
	(3.28–6.30)	(0.47–0.95)	(0.74–1.07)	(0.46–0.79)	(0.30–0.59)	(1.98–3.04)	(0.55–0.93)	(0.00–0.08)	(1.09–1.75)	(0.23–0.37)
**Cambodia**										
Total	4.83	0.86	0.69	0.32	0.55	3.49	0.11	0.98	1.50	0.27
	(3.46–6.51)	(0.63–1.17)	(0.58–0.83)	(0.24–0.41)	(0.39–0.71)	(2.84–4.18)	(0.08–0.14)	(0.52–1.49)	(1.18–1.91)	(0.21–0.35)
Male	3.46	1.19	0.95	0.31	0.69	3.10	0.09	0.82	1.81	0.30
	(2.46–4.75)	(0.87–1.66)	(0.78–1.13)	(0.24–0.41)	(0.50–0.88)	(2.50–3.71)	(0.06–0.12)	(0.36–1.32)	(1.43–2.31)	(0.23–0.38)
Female	6.07	0.52	0.45	0.32	0.40	3.84	0.12	1.13	1.24	0.25
	(4.28–8.24)	(0.37–0.75)	(0.37–0.54)	(0.24–0.41)	(0.28–0.55)	(3.10–4.61)	(0.09–0.17)	(0.64–1.66)	(0.97–1.57)	(0.19–0.32)
**Indonesia**										
Total	4.39	0.90	0.67	0.33	0.61	2.91	0.14	0.70	1.45	0.30
	(3.80–5.17)	(0.64–1.23)	(0.56–0.80)	(0.28–0.38)	(0.44–0.77)	(2.59–3.28)	(0.10–0.19)	(0.30–1.08)	(1.14–1.80)	(0.25–0.35)
Male	3.24	1.25	0.85	0.33	0.74	2.46	0.12	0.60	1.72	0.33
	(2.77–3.80)	(0.89–1.71)	(0.72–1.00)	(0.28–0.38)	(0.55–0.93)	(2.18–2.76)	(0.09–0.16)	(0.20–0.98)	(1.34–2.16)	(0.28–0.39)
Female	5.54	0.54	0.49	0.33	0.47	3.36	0.17	0.80	1.19	0.27
	(4.79–6.52)	(0.38–0.75)	(0.41–0.59)	(0.28–0.38)	(0.33–0.62)	(2.97–3.79)	(0.12–0.22)	(0.40–1.18)	(0.93–1.47)	(0.22–0.32)
**Laos**										
Total	5.24	0.86	0.69	0.33	0.55	3.13	0.13	0.80	1.53	0.28
	(3.75–7.17)	(0.63–1.18)	(0.58–0.83)	(0.25–0.42)	(0.39–0.72)	(2.54–3.86)	(0.09–0.17)	(0.38–1.22)	(1.20–1.94)	(0.22–0.35)
Male	4.11	1.20	0.94	0.33	0.69	3.09	0.10	0.65	1.82	0.31
	(2.93–5.66)	(0.88–1.67)	(0.78–1.11)	(0.25–0.42)	(0.50–0.89)	(2.47–3.76)	(0.08–0.14)	(0.22–1.06)	(1.44–2.32)	(0.24–0.39)
Female	6.35	0.52	0.45	0.33	0.41	3.17	0.15	0.96	1.24	0.25
	(4.51–8.64)	(0.37–0.76)	(0.37–0.54)	(0.25–0.42)	(0.28–0.55)	(2.55–3.90)	(0.11–0.20)	(0.53–1.39)	(0.97–1.58)	(0.19–0.32)
**Malaysia**										
Total	5.51	0.42	0.77	0.33	0.57	3.88	0.19	0.35	1.57	0.32
	(3.96–7.54)	(0.30–0.59)	(0.64–0.92)	(0.25–0.42)	(0.40–0.73)	(3.20–4.77)	(0.14–0.26)	(0.07–0.64)	(1.24–1.99)	(0.25–0.41)
Male	4.04	0.56	1.05	0.33	0.71	3.55	0.16	0.29	1.87	0.35

	(2.89–5.59)	(0.40–0.77)	(0.87–1.25)	(0.25–0.43)	(0.52–0.91)	(2.87–4.39)	(0.12–0.22)	(0.03–0.57)	(1.48–2.39)	(0.27–0.45)
Female	7.03	0.27	0.49	0.33	0.41	4.22	0.22	0.41	1.26	0.29
	(5.02–9.61)	(0.19–0.40)	(0.40–0.59)	(0.25–0.42)	(0.28–0.56)	(3.45–5.17)	(0.17–0.30)	(0.12–0.69)	(0.99–1.61)	(0.22–0.37)
**Myanmar**										
Total	4.76	0.85	0.69	0.32	0.55	2.69	0.12	0.79	1.50	0.28
	(3.41–6.32)	(0.63–1.17)	(0.57–0.83)	(0.24–0.40)	(0.39–0.71)	(2.21–3.24)	(0.09–0.17)	(0.35–1.24)	(1.18–1.91)	(0.22–0.35)
Male	3.42	1.19	0.95	0.31	0.69	2.47	0.10	0.66	1.81	0.31
	(2.46–4.71)	(0.87–1.66)	(0.78–1.13)	(0.24–0.40)	(0.50–0.88)	(2.03–2.99)	(0.07–0.14)	(0.22–1.09)	(1.43–2.30)	(0.23–0.39)
Female	5.95	0.52	0.45	0.32	0.40	2.87	0.14	0.92	1.24	0.25
	(4.15–7.85)	(0.37–0.75)	(0.37–0.54)	(0.24–0.41)	(0.28–0.55)	(2.37–3.52)	(0.11–0.19)	(0.48–1.38)	(0.97–1.58)	(0.20–0.32)
**Philippines**										
Total	4.97	0.90	0.71	0.33	0.61	3.53	0.13	0.67	1.45	0.30
	(4.27–5.72)	(0.64–1.23)	(0.60–0.84)	(0.28–0.38)	(0.44–0.77)	(3.14–3.97)	(0.10–0.18)	(0.25–1.08)	(1.13–1.80)	(0.25–0.35)
Male	3.61	1.24	0.96	0.32	0.74	3.18	0.11	0.58	1.71	0.32
	(3.10–4.15)	(0.89–1.71)	(0.81–1.13)	(0.27–0.38)	(0.55–0.93)	(2.83–3.59)	(0.08–0.15)	(0.15–1.00)	(1.34–2.15)	(0.27–0.38)
Female	6.31	0.54	0.46	0.33	0.47	3.87	0.15	0.77	1.19	0.27
	(5.42–7.27)	(0.38–0.75)	(0.38–0.55)	(0.28–0.39)	(0.33–0.62)	(3.45–4.36)	(0.11–0.20)	(0.35–1.18)	(0.93–1.47)	(0.22–0.32)
**Singapore**										
Total	3.45	1.39	1.65	0.67	0.62	2.49	0.56	0.01	1.79	0.28
	(2.45–4.67)	(1.00–1.93)	(1.39–1.97)	(0.51–0.86)	(0.44–0.81)	(2.04–3.05)	(0.43–0.74)	(0.00–0.11)	(1.41–2.27)	(0.22–0.36)
Male	2.58	2.12	2.33	0.61	0.80	2.16	0.42	0.02	2.19	0.26
	(1.83–3.56)	(1.52–2.92)	(1.96–2.78)	(0.47–0.79)	(0.59–1.03)	(1.79–2.63)	(0.30–0.56)	(0.00–0.16)	(1.72–2.76)	(0.21–0.34)
Female	4.31	0.68	0.97	0.72	0.44	2.81	0.71	0.01	1.39	0.30
	(3.02–5.87)	(0.48–0.97)	(0.79–1.17)	(0.54–0.94)	(0.30–0.60)	(2.28–3.53)	(0.53–0.92)	(0.00–0.06)	(1.11–1.78)	(0.24–0.39)
**Thailand**										
Total	3.97	1.90	0.76	0.33	0.56	3.30	0.17	0.73	1.54	0.30
	(2.77–5.43)	(1.41–2.59)	(0.63–0.91)	(0.25–0.41)	(0.40–0.73)	(2.74–3.92)	(0.12–0.23)	(0.27–1.19)	(1.21–1.96)	(0.23–0.39)
Male	2.89	2.77	1.05	0.32	0.71	2.89	0.14	0.97	1.85	0.33
	(2.02–3.99)	(2.06–3.76)	(0.87–1.26)	(0.25–0.41)	(0.51–0.90)	(2.36–3.46)	(0.10–0.19)	(0.38–1.57)	(1.47–2.36)	(0.26–0.42)
Female	4.96	1.04	0.48	0.33	0.41	3.68	0.19	0.50	1.26	0.28
	(3.43–6.84)	(0.77–1.46)	(0.40–0.58)	(0.25–0.42)	(0.28–0.56)	(3.04–4.43)	(0.14–0.26)	(0.18–0.81)	(0.99–1.61)	(0.21–0.36)
**Viet Nam**										
Total	2.68	0.88	0.76	0.33	0.56	2.85	0.13	0.53	1.54	0.33
	(1.91–3.61)	(0.65–1.20)	(0.63–0.90)	(0.25–0.41)	(0.40–0.73)	(2.36–3.37)	(0.09–0.17)	(0.24–0.86)	(1.21–1.95)	(0.26–0.41)
Male	1.88	1.21	1.09	0.32	0.70	2.19	0.11	0.19	1.84	0.36
	(1.34–2.54)	(0.89–1.70)	(0.90–1.29)	(0.24–0.41)	(0.51–0.90)	(1.80–2.63)	(0.08–0.15)	(0.02–0.42)	(1.46–2.35)	(0.28–0.45)
Female	3.46	0.53	0.43	0.33	0.41	3.46	0.15	0.89	1.25	0.31
	(2.44–4.70)	(0.37–0.76)	(0.36–0.52)	(0.25–0.42)	(0.28–0.56)	(2.85–4.16)	(0.11–0.20)	(0.47–1.31)	(0.98–1.60)	(0.24–0.38)

95% UI=95% uncertainty interval.

Table S5 | ASEAN prevalence (95% UI) (in %) of mental disorders by age group and sex, 2021. ASEAN=Association of Southeast Asian Nations.

	Anxiety disorders	Attention-deficit hyperactivity disorder	Autism spectrum disorders	Bipolar disorder	Conduct disorder	Depressive disorders	Eating disorders	Idiopathic developmental intellectual disability	Other mental disorders	Schizophrenia
**All ages**										
Total	4.44 (3.74–5.25)	0.93 (0.69–1.27)	0.71 (0.60–0.85)	0.35 (0.28–0.42)	0.54 (0.39–0.69)	3.25 (2.87–3.69)	0.15 (0.11–0.20)	0.66 (0.28–1.04)	1.57 (1.22–1.97)	0.33 (0.27–0.40)
Male	3.23 (2.71–3.85)	1.32 (0.97–1.80)	0.96 (0.81–1.14)	0.34 (0.28–0.42)	0.69 (0.51–0.87)	2.76 (2.44–3.15)	0.13 (0.09–0.17)	0.55 (0.17–0.93)	1.83 (1.43–2.33)	0.36 (0.29–0.43)
Female	5.62 (4.74–6.70)	0.54 (0.39–0.75)	0.47 (0.39–0.57)	0.36 (0.29–0.43)	0.40 (0.28–0.53)	3.73 (3.28–4.23)	0.17 (0.13–0.23)	0.76 (0.37–1.14)	1.30 (1.02–1.61)	0.30 (0.24–0.36)
**<5 years**										
Total	0.12 (0.08–0.18)	0.18 (0.12–0.27)	0.88 (0.73–1.05)	0.00 (0.00–0.00)	0.00 (0.00–0.00)	0.00 (0.00–0.00)	0.00 (0.00–0.00)	0.94 (0.39–1.49)	0.00 (0.00–0.00)	0.00 (0.00–0.00)
Male	0.09 (0.06–0.13)	0.26 (0.17–0.38)	1.16 (0.97–1.38)	0.00 (0.00–0.00)	0.00 (0.00–0.00)	0.00 (0.00–0.00)	0.00 (0.00–0.00)	0.78 (0.25–1.33)	0.00 (0.00–0.00)	0.00 (0.00–0.00)
Female	0.15 (0.10–0.22)	0.11 (0.07–0.16)	0.58 (0.48–0.70)	0.00 (0.00–0.00)	0.00 (0.00–0.00)	0.00 (0.00–0.00)	0.00 (0.00–0.00)	1.11 (0.54–1.67)	0.00 (0.00–0.00)	0.00 (0.00–0.00)
**5-9 years**										
Total	1.68 (1.12–2.46)	1.88 (1.26–2.78)	0.84 (0.69–1.00)	0.00 (0.00–0.00)	1.23 (0.81–1.77)	0.11 (0.07–0.18)	0.01 (0.00–0.01)	1.01 (0.48–1.55)	0.00 (0.00–0.00)	0.00 (0.00–0.00)
Male	1.27 (0.84–1.84)	2.65 (1.79–3.90)	1.11 (0.92–1.32)	0.00 (0.00–0.00)	1.56 (1.07–2.21)	0.10 (0.06–0.16)	0.01 (0.00–0.01)	0.87 (0.34–1.41)	0.00 (0.00–0.00)	0.00 (0.00–0.00)
Female	2.11 (1.40–3.07)	1.09 (0.72–1.65)	0.55 (0.46–0.66)	0.00 (0.00–0.00)	0.90 (0.57–1.31)	0.13 (0.07–0.20)	0.01 (0.00–0.02)	1.16 (0.62–1.70)	0.00 (0.00–0.00)	0.00 (0.00–0.00)
**10-14 years**										
Total	4.10 (2.83–5.72)	2.47 (1.67–3.60)	0.79 (0.67–0.93)	0.06 (0.04–0.08)	3.64 (2.49–4.98)	1.11 (0.72–1.60)	0.09 (0.06–0.14)	0.95 (0.44–1.44)	0.06 (0.04–0.09)	0.01 (0.01–0.02)
Male	3.12 (2.15–4.34)	3.51 (2.36–5.11)	1.06 (0.90–1.24)	0.06 (0.04–0.09)	4.42 (3.08–5.98)	0.94 (0.61–1.35)	0.08 (0.05–0.13)	0.83 (0.31–1.33)	0.08 (0.05–0.11)	0.01 (0.01–0.02)
Female	5.08 (3.53–7.13)	1.43 (0.95–2.15)	0.52 (0.44–0.62)	0.06 (0.03–0.08)	2.86 (1.86–4.04)	1.28 (0.84–1.85)	0.10 (0.07–0.15)	1.07 (0.56–1.55)	0.05 (0.03–0.07)	0.01 (0.01–0.02)
**15-19 years**										
Total	5.14 (3.93–6.61)	1.92 (1.31–2.74)	0.75 (0.63–0.89)	0.28 (0.19–0.40)	1.87 (1.29–2.67)	2.69 (1.96–3.60)	0.31 (0.19–0.49)	0.85 (0.38–1.31)	0.41 (0.25–0.58)	0.09 (0.06–0.14)
Male	3.84 (2.87–4.97)	2.71 (1.85–3.82)	1.00 (0.84–1.18)	0.28 (0.19–0.41)	2.42 (1.68–3.32)	2.35 (1.71–3.16)	0.26 (0.16–0.44)	0.75 (0.28–1.22)	0.50 (0.31–0.72)	0.10 (0.06–0.15)
Female	6.45 (4.97–8.28)	1.13 (0.76–1.63)	0.50 (0.41–0.59)	0.27 (0.19–0.40)	1.32 (0.88–2.00)	3.04 (2.24–4.04)	0.35 (0.23–0.54)	0.96 (0.50–1.40)	0.31 (0.19–0.45)	0.08 (0.05–0.12)
**20-24 years**										
Total	5.37 (3.93–7.16)	1.38 (0.93–1.95)	0.73 (0.62–0.87)	0.41 (0.28–0.57)	0.11 (0.06–0.17)	3.39 (2.46–4.68)	0.39 (0.24–0.62)	0.78 (0.34–1.21)	1.00 (0.65–1.42)	0.31 (0.20–0.45)
Male	3.94 (2.86–5.24)	1.94 (1.31–2.69)	0.97 (0.82–1.15)	0.41 (0.28–0.57)	0.17 (0.10–0.26)	3.04 (2.21–4.17)	0.32 (0.18–0.54)	0.68 (0.24–1.12)	1.22 (0.80–1.75)	0.34 (0.22–0.49)
Female	6.81 (4.99–9.18)	0.82 (0.55–1.18)	0.49 (0.41–0.59)	0.41 (0.29–0.58)	0.05 (0.02–0.09)	3.74 (2.72–5.16)	0.46 (0.29–0.69)	0.88 (0.45–1.30)	0.77 (0.49–1.09)	0.28 (0.18–0.41)
**25-29 years**										

Total	5.35	1.01	0.72	0.46	0.00	3.47	0.36	0.71	1.57	0.49
	(3.89–7.12)	(0.70–1.43)	(0.61–0.86)	(0.32–0.64)	(0.00–0.00)	(2.77–4.34)	(0.23–0.53)	(0.31–1.11)	(1.14–2.12)	(0.34–0.66)
Male	3.88	1.41	0.96	0.45	0.00	3.02	0.30	0.59	1.91	0.54
	(2.77–5.22)	(0.96–2.02)	(0.81–1.14)	(0.32–0.63)	(0.00–0.00)	(2.41–3.78)	(0.19–0.45)	(0.20–0.99)	(1.38–2.57)	(0.37–0.72)
Female	6.84	0.61	0.48	0.46	0.00	3.93	0.42	0.83	1.23	0.45
	(4.97–9.13)	(0.42–0.89)	(0.40–0.58)	(0.33–0.64)	(0.00–0.00)	(3.12–4.94)	(0.28–0.63)	(0.42–1.23)	(0.89–1.67)	(0.31–0.60)
**30-34 years**										
Total	5.30	0.78	0.72	0.46	0.00	3.67	0.31	0.65	2.04	0.57
	(4.04–6.79)	(0.52–1.10)	(0.60–0.85)	(0.33–0.62)	(0.00–0.00)	(2.93–4.57)	(0.21–0.45)	(0.28–1.02)	(1.55–2.63)	(0.43–0.74)
Male	3.82	1.09	0.95	0.45	0.00	3.11	0.27	0.52	2.45	0.62
	(2.93–4.94)	(0.71–1.53)	(0.80–1.13)	(0.33–0.61)	(0.00–0.00)	(2.47–3.87)	(0.16–0.41)	(0.16–0.88)	(1.85–3.17)	(0.47–0.80)
Female	6.81	0.47	0.48	0.47	0.00	4.24	0.35	0.78	1.63	0.52
	(5.16–8.73)	(0.32–0.70)	(0.40–0.57)	(0.34–0.63)	(0.00–0.00)	(3.39–5.31)	(0.24–0.50)	(0.39–1.16)	(1.21–2.12)	(0.39–0.68)
**35-39 years**										
Total	5.29	0.64	0.71	0.46	0.00	4.10	0.23	0.59	2.36	0.59
	(4.08–6.71)	(0.43–0.91)	(0.60–0.84)	(0.34–0.60)	(0.00–0.00)	(3.33–4.92)	(0.15–0.33)	(0.24–0.94)	(1.80–3.00)	(0.46–0.75)
Male	3.80	0.89	0.94	0.45	0.00	3.40	0.19	0.47	2.80	0.64
	(2.90–4.82)	(0.59–1.28)	(0.79–1.11)	(0.34–0.60)	(0.00–0.00)	(2.79–4.14)	(0.12–0.29)	(0.13–0.81)	(2.14–3.56)	(0.50–0.82)
Female	6.81	0.39	0.47	0.47	0.00	4.81	0.26	0.71	1.90	0.54
	(5.27–8.50)	(0.25–0.55)	(0.39–0.57)	(0.35–0.62)	(0.00–0.00)	(3.85–5.75)	(0.17–0.38)	(0.34–1.07)	(1.44–2.43)	(0.42–0.69)
**40-44 years**										
Total	5.28	0.53	0.70	0.48	0.00	4.53	0.12	0.53	2.52	0.57
	(3.83–6.80)	(0.35–0.77)	(0.59–0.83)	(0.35–0.63)	(0.00–0.00)	(3.56–5.76)	(0.08–0.18)	(0.20–0.86)	(1.96–3.20)	(0.46–0.70)
Male	3.78	0.74	0.93	0.46	0.00	3.73	0.09	0.42	2.98	0.62
	(2.66–5.03)	(0.48–1.08)	(0.78–1.10)	(0.34–0.61)	(0.00–0.00)	(2.94–4.65)	(0.05–0.14)	(0.10–0.73)	(2.31–3.79)	(0.50–0.76)
Female	6.79	0.32	0.47	0.49	0.00	5.32	0.15	0.64	2.06	0.52
	(4.98–8.68)	(0.21–0.46)	(0.39–0.56)	(0.36–0.65)	(0.00–0.00)	(4.17–6.74)	(0.10–0.22)	(0.29–0.98)	(1.59–2.60)	(0.42–0.64)
**45-49 years**										
Total	5.20	0.44	0.68	0.50	0.00	4.81	0.05	0.47	2.61	0.53
	(3.78–6.77)	(0.28–0.64)	(0.57–0.81)	(0.37–0.65)	(0.00–0.00)	(3.97–5.90)	(0.03–0.08)	(0.17–0.78)	(2.02–3.30)	(0.43–0.65)
Male	3.74	0.61	0.91	0.48	0.00	4.00	0.03	0.37	3.06	0.58
	(2.70–4.89)	(0.39–0.87)	(0.76–1.07)	(0.36–0.63)	(0.00–0.00)	(3.29–4.94)	(0.02–0.05)	(0.08–0.67)	(2.38–3.90)	(0.47–0.71)
Female	6.66	0.27	0.46	0.51	0.00	5.61	0.07	0.58	2.15	0.49
	(4.82–8.72)	(0.17–0.39)	(0.38–0.55)	(0.38–0.67)	(0.00–0.00)	(4.64–6.90)	(0.04–0.11)	(0.26–0.89)	(1.68–2.72)	(0.39–0.59)
**50-54 years**										
Total	5.09	0.34	0.66	0.52	0.00	4.97	0.00	0.42	2.64	0.49
	(3.95–6.51)	(0.22–0.49)	(0.56–0.79)	(0.39–0.67)	(0.00–0.00)	(4.21–5.94)	(0.00–0.00)	(0.14–0.70)	(2.04–3.31)	(0.39–0.59)
Male	3.68	0.47	0.89	0.51	0.00	4.19	0.00	0.33	3.09	0.53
	(2.85–4.61)	(0.30–0.69)	(0.75–1.05)	(0.38–0.67)	(0.00–0.00)	(3.56–5.02)	(0.00–0.00)	(0.06–0.60)	(2.39–3.90)	(0.43–0.65)
Female	6.46	0.21	0.44	0.53	0.00	5.72	0.00	0.51	2.20	0.44
	(4.93–8.37)	(0.13–0.30)	(0.37–0.53)	(0.40–0.69)	(0.00–0.00)	(4.83–6.89)	(0.00–0.00)	(0.21–0.80)	(1.70–2.75)	(0.36–0.54)
**55-59 years**										
Total	4.95	0.23	0.64	0.54	0.00	5.06	0.00	0.37	2.65	0.43
	(3.95–6.28)	(0.14–0.34)	(0.54–0.76)	(0.40–0.72)	(0.00–0.00)	(4.21–6.10)	(0.00–0.00)	(0.11–0.62)	(2.08–3.32)	(0.35–0.52)
Male	3.63	0.32	0.87	0.53	0.00	4.33	0.00	0.28	3.10	0.47
	(2.83–4.66)	(0.20–0.47)	(0.73–1.03)	(0.39–0.70)	(0.00–0.00)	(3.61–5.23)	(0.00–0.00)	(0.05–0.53)	(2.42–3.91)	(0.38–0.57)
Female	6.21	0.14	0.43	0.54	0.00	5.74	0.00	0.45	2.23	0.39
	(4.92–7.79)	(0.08–0.21)	(0.36–0.51)	(0.40–0.72)	(0.00–0.00)	(4.78–6.97)	(0.00–0.00)	(0.17–0.71)	(1.75–2.79)	(0.32–0.47)

**60-64 years**										
Total	4.83	0.13	0.62	0.51	0.00	5.04	0.00	0.32	2.67	0.36
	(3.64–6.49)	(0.07–0.20)	(0.51–0.73)	(0.38–0.66)	(0.00–0.00)	(4.09–6.20)	(0.00–0.00)	(0.09–0.55)	(2.11–3.35)	(0.30–0.44)
Male	3.58	0.18	0.84	0.51	0.00	4.39	0.00	0.24	3.11	0.40
	(2.58–4.91)	(0.10–0.28)	(0.70–1.00)	(0.38–0.66)	(0.00–0.00)	(3.58–5.40)	(0.00–0.00)	(0.03–0.47)	(2.44–3.95)	(0.33–0.48)
Female	5.98	0.08	0.41	0.51	0.00	5.64	0.00	0.39	2.26	0.33
	(4.58–8.12)	(0.04–0.13)	(0.34–0.49)	(0.39–0.66)	(0.00–0.00)	(4.56–6.91)	(0.00–0.00)	(0.13–0.63)	(1.79–2.84)	(0.28–0.40)
**65-69 years**										
Total	4.73	0.06	0.57	0.47	0.00	4.80	0.00	0.27	2.68	0.30
	(3.49–6.52)	(0.03–0.10)	(0.47–0.67)	(0.35–0.60)	(0.00–0.00)	(3.96–5.88)	(0.00–0.00)	(0.07–0.47)	(2.17–3.38)	(0.25–0.36)
Male	3.48	0.08	0.79	0.47	0.00	4.25	0.00	0.20	3.13	0.33
	(2.51–4.82)	(0.04–0.15)	(0.65–0.93)	(0.35–0.61)	(0.00–0.00)	(3.49–5.30)	(0.00–0.00)	(0.03–0.40)	(2.50–3.94)	(0.27–0.39)
Female	5.82	0.04	0.37	0.46	0.00	5.28	0.00	0.33	2.29	0.28
	(4.34–8.09)	(0.02–0.07)	(0.31–0.44)	(0.35–0.60)	(0.00–0.00)	(4.31–6.50)	(0.00–0.00)	(0.11–0.55)	(1.84–2.89)	(0.23–0.33)
**70-74 years**										
Total	4.64	0.03	0.50	0.41	0.00	4.50	0.00	0.24	2.71	0.24
	(3.50–6.33)	(0.01–0.05)	(0.41–0.59)	(0.31–0.54)	(0.00–0.00)	(3.66–5.45)	(0.00–0.00)	(0.07–0.42)	(2.21–3.35)	(0.20–0.29)
Male	3.35	0.04	0.71	0.42	0.00	4.05	0.00	0.18	3.15	0.26
	(2.53–4.57)	(0.01–0.07)	(0.58–0.84)	(0.32–0.55)	(0.00–0.00)	(3.30–4.88)	(0.00–0.00)	(0.03–0.36)	(2.55–3.91)	(0.21–0.32)
Female	5.71	0.02	0.32	0.41	0.00	4.87	0.00	0.29	2.34	0.22
	(4.28–7.87)	(0.00–0.04)	(0.26–0.39)	(0.31–0.53)	(0.00–0.00)	(3.96–5.91)	(0.00–0.00)	(0.11–0.47)	(1.89–2.90)	(0.18–0.27)
**75-79 years**										
Total	4.53	0.01	0.41	0.35	0.00	4.17	0.00	0.22	2.75	0.19
	(3.41–6.01)	(0.00–0.02)	(0.33–0.49)	(0.27–0.45)	(0.00–0.00)	(3.34–5.06)	(0.00–0.00)	(0.06–0.38)	(2.24–3.37)	(0.15–0.23)
Male	3.18	0.01	0.61	0.36	0.00	3.81	0.00	0.16	3.21	0.20
	(2.39–4.12)	(0.00–0.03)	(0.49–0.73)	(0.28–0.46)	(0.00–0.00)	(3.05–4.64)	(0.00–0.00)	(0.02–0.31)	(2.60–3.94)	(0.17–0.24)
Female	5.53	0.01	0.26	0.35	0.00	4.44	0.00	0.26	2.40	0.18
	(4.16–7.39)	(0.00–0.02)	(0.21–0.32)	(0.27–0.45)	(0.00–0.00)	(3.55–5.38)	(0.00–0.00)	(0.10–0.42)	(1.96–2.94)	(0.14–0.21)
**80-84 years**										
Total	4.37	0.00	0.33	0.30	0.00	4.00	0.00	0.19	2.83	0.14
	(3.23–5.62)	(0.00–0.00)	(0.25–0.40)	(0.22–0.39)	(0.00–0.00)	(3.13–5.00)	(0.00–0.00)	(0.06–0.33)	(2.30–3.46)	(0.11–0.17)
Male	2.97	0.00	0.51	0.30	0.00	3.65	0.00	0.14	3.31	0.15
	(2.14–3.90)	(0.00–0.00)	(0.40–0.63)	(0.23–0.40)	(0.00–0.00)	(2.86–4.56)	(0.00–0.00)	(0.03–0.28)	(2.70–4.04)	(0.12–0.18)
Female	5.28	0.00	0.20	0.29	0.00	4.23	0.00	0.23	2.51	0.13
	(3.93–6.88)	(0.00–0.00)	(0.16–0.26)	(0.22–0.38)	(0.00–0.00)	(3.30–5.29)	(0.00–0.00)	(0.08–0.36)	(2.04–3.07)	(0.11–0.17)
**85-89 years**										
Total	3.99	0.00	0.25	0.25	0.00	4.19	0.00	0.17	3.00	0.10
	(2.80–5.22)	(0.00–0.00)	(0.19–0.33)	(0.18–0.34)	(0.00–0.00)	(3.42–5.26)	(0.00–0.00)	(0.05–0.28)	(2.43–3.64)	(0.08–0.13)
Male	2.59	0.00	0.42	0.26	0.00	3.74	0.00	0.13	3.51	0.10
	(1.81–3.46)	(0.00–0.00)	(0.31–0.54)	(0.18–0.34)	(0.00–0.00)	(3.01–4.71)	(0.00–0.00)	(0.03–0.24)	(2.85–4.27)	(0.08–0.13)
Female	4.81	0.00	0.15	0.25	0.00	4.45	0.00	0.19	2.70	0.10
	(3.41–6.25)	(0.00–0.00)	(0.11–0.20)	(0.17–0.33)	(0.00–0.00)	(3.60–5.59)	(0.00–0.00)	(0.07–0.30)	(2.19–3.28)	(0.08–0.13)
**90-94 years**										
Total	3.45	0.00	0.19	0.21	0.00	4.52	0.00	0.14	3.31	0.07
	(2.37–4.77)	(0.00–0.00)	(0.13–0.26)	(0.15–0.29)	(0.00–0.00)	(3.49–5.69)	(0.00–0.00)	(0.04–0.24)	(2.65–4.08)	(0.05–0.09)
Male	2.13	0.00	0.33	0.22	0.00	4.01	0.00	0.11	3.84	0.07
	(1.44–3.02)	(0.00–0.00)	(0.23–0.45)	(0.15–0.29)	(0.00–0.00)	(3.12–5.06)	(0.00–0.00)	(0.02–0.22)	(3.08–4.75)	(0.05–0.09)
Female	4.18	0.00	0.11	0.21	0.00	4.81	0.00	0.15	3.01	0.07

	(2.84–5.81)	(0.00–0.00)	(0.07–0.16)	(0.14–0.28)	(0.00–0.00)	(3.73–6.09)	(0.00–0.00)	(0.06–0.25)	(2.41–3.72)	(0.05–0.09)
**95+ years**										
Total	2.72	0.00	0.14	0.18	0.00	4.93	0.00	0.11	3.81	0.05
	(1.77–4.06)	(0.00–0.00)	(0.09–0.21)	(0.12–0.25)	(0.00–0.00)	(3.44–6.67)	(0.00–0.00)	(0.03–0.20)	(2.97–5.04)	(0.03–0.07)
Male	1.60	0.00	0.26	0.18	0.00	4.41	0.00	0.10	4.38	0.04
	(0.99–2.42)	(0.00–0.00)	(0.16–0.37)	(0.12–0.26)	(0.00–0.00)	(3.12–6.00)	(0.00–0.00)	(0.02–0.20)	(3.40–5.72)	(0.03–0.07)
Female	3.39	0.00	0.08	0.18	0.00	5.25	0.00	0.12	3.47	0.05
	(2.17–5.03)	(0.00–0.00)	(0.04–0.12)	(0.12–0.25)	(0.00–0.00)	(3.65–7.10)	(0.00–0.00)	(0.04–0.20)	(2.71–4.61)	(0.03–0.07)

95% UI=95% uncertainty interval.

Table S6 | Premature deaths (95% UI) attributable to eating disorders in ASEAN and its member states. ASEAN=Association of Southeast Asian Nations.

	Deaths in 1990 (95% UI)	Deaths in 2019 (95% UI)	Deaths in 2021 (95% UI)	Percentage change from 1990 to 2021	Contribution to global deaths (in %) in 2021
**ASEAN**	1.46 (0.73–2.47)	3.27 (1.41–5.27)	3.42 (1.52–5.36)	1.35 (0.72–2.43)	1.48
**Brunei**	0.01 (0.00–0.05)	0.06 (0.02–0.13)	0.05 (0.02–0.11)	2.66 (0.50–12.87)	0.02
**Cambodia**	0.00 (0.00–0.00)	0.03 (0.01–0.08)	0.03 (0.01–0.09)	72.19 (20.75–11232.21)	0.01
**Indonesia**	0.26 (0.08–0.60)	1.48 (0.61–2.66)	1.53 (0.60–2.84)	4.97 (2.44–11.09)	0.66
**Laos**	0.00 (0.00–0.00)	0.01 (0.00–0.03)	0.01 (0.00–0.04)	30.71 (8.60–877.60)	0.01
**Malaysia**	0.04 (0.01–0.10)	0.16 (0.03–0.37)	0.17 (0.03–0.36)	3.10 (1.01–10.50)	0.07
**Myanmar**	0.02 (0.00–0.06)	0.15 (0.04–0.36)	0.16 (0.04–0.38)	6.71 (1.44–60.37)	0.07
**Philippines**	0.81 (0.43–1.21)	0.37 (0.23–0.45)	0.43 (0.27–0.56)	-0.47 (-0.66–-0.06)	0.19
**Singapore**	0.12 (0.10–0.15)	0.15 (0.12–0.20)	0.15 (0.11–0.20)	0.24 (-0.12–0.70)	0.07
**Thailand**	0.13 (0.02–0.35)	0.42 (0.13–0.85)	0.42 (0.14–0.86)	2.14 (0.44–8.05)	0.18
**Viet Nam**	0.06 (0.01–0.16)	0.44 (0.12–0.94)	0.47 (0.12–0.98)	6.23 (1.81–31.32)	0.20

95% UI=95% uncertainty interval.

Table S7 | YLLs (95% UI) attributable to eating disorders in ASEAN and its member states.

YLLs = Years of Life Lost. ASEAN=Association of Southeast Asian Nations.

	1990		2019		2021		Percentage increase from 1990 to 2021	
	Total YLLs (95% UI)	Age-standardized YLLs per 100 000 (95% UI)	Total YLLs (95% UI)	Age-standardized YLLs per 100 000 (95% UI)	Total YLLs (95% UI)	Age-standardized YLLs per 100 000 (95% UI)	Total YLLs (95% UI)	Age-standardized YLLs per 100,000 (95% UI)
**ASEAN**	83.0	0.02	178.7	0.02	186.4	0.03	124.6	32.1
	(41.6–141.2)	(0.01–0.03)	(76.3–292.3)	(0.01–0.04)	(81.8–295.1)	(0.01–0.04)	(63.3–231.7)	(-3.2–95.3)
**Brunei**	0.88	0.28	3.45	0.64	2.96	0.53	235.9	88.2
	(0.15–3.19)	(0.05–1.03)	(1.18–7.33)	(0.21–1.37)	(1.08–6.32)	(0.19–1.15)	(32.3–1 171.9)	(-28.1–591.5)
**Cambodia**	0.03	0.00	1.73	0.01	1.87	0.01	6 844.9	3 450.6
	(0.00–0.13)	(0.00–0.00)	(0.32–4.70)	(0.00–0.03)	(0.32–5.16)	(0.00–0.03)	(1 933.3–883 246.4)	(956.4–603 694.2)
**Indonesia**	14.9	0.01	80.8	0.03	83.5	0.03	460.4	232.3
	(4.79–35.0)	(0.00–0.02)	(32.4–146.9)	(0.01–0.05)	(31.9–158.2)	(0.01–0.05)	(217.9–1 062.4)	(92.6–579.0)
**Laos**	0.02	0.00	0.61	0.01	0.68	0.01	2 845.2	1 344.6
	(0.00–0.11)	(0.00–0.00)	(0.09–1.87)	(0.00–0.02)	(0.10–2.05)	(0.00–0.03)	(775.4–75 765.4)	(337.7–41 405.2)
**Malaysia**	2.31	0.01	8.70	0.03	8.97	0.03	287.8	93.4
	(0.36–6.05)	(0.00–0.03)	(1.74–20.0)	(0.01–0.06)	(1.75–19.1)	(0.00–0.06)	(91.3–963.6)	(-3.8–420.4)
**Myanmar**	1.20	0.00	8.37	0.01	8.84	0.01	634.6	401.7
	(0.07–3.78)	(0.00–0.01)	(2.09–20.9)	(0.00–0.04)	(2.19–21.6)	(0.00–0.04)	(127.9–5 698.9)	(55.6–3 955.6)
**Philippines**	45.2	0.08	20.5	0.02	23.7	0.02	-47.7	-74.4
	(23.9–67.7)	(0.04–0.12)	(12.4–24.9)	(0.01–0.02)	(14.8–31.1)	(0.01–0.03)	(-66.7–-8.5)	(-83.6–-55.0)
**Singapore**	7.27	0.19	8.80	0.16	8.77	0.18	20.5	-3.5
	(5.63–8.84)	(0.15–0.23)	(6.79–11.2)	(0.12–0.21)	(6.49–11.4)	(0.13–0.26)	(-13.8–68.3)	(-33.0–45.6)
**Thailand**	7.44	0.01	22.0	0.03	22.1	0.03	197.0	160.4
	(1.24–19.3)	(0.00–0.03)	(7.04–43.7)	(0.01–0.06)	(7.19–46.3)	(0.01–0.07)	(37.4–742.1)	(16.0–639.8)
**Viet Nam**	3.70	0.01	23.7	0.02	25.0	0.02	577.3	265.3
	(0.41–9.19)	(0.00–0.02)	(5.97–50.2)	(0.01–0.05)	(6.35–52.0)	(0.01–0.05)	(163.4–3 033.0)	(38.5–1 510.1)

95% UI=95% uncertainty interval.

Table S8 | ASEAN number and rate of DALYs attributable to mental disorders in 1990, 2019, and 2021, and the percentage change from 1990 to 2021, by country and sex. The 2019 DALYs data serve as a reference for pre-COVID levels. DALYs=disability-adjusted life-years. ASEAN=Association of Southeast Asian Nations.

	**1990**		**2019**		**2021**		**Percentage change from 1990 to 2021**	
	DALYs, in thousands (95% UI)	Age-standardised DALYs per 100 000 (95% UI)	DALYs, in thousands (95% UI)	Age-standardised DALYs per 100 000 (95% UI)	DALYs, in thousands (95% UI)	Age-standardised DALYs per 100 000 (95% UI)	DALYs (95% UI)	Age-standardised DALYs (95% UI)
**ASEAN**								
Total	5 993.4	1 433.4	9 893.0	1 431.6	11 231.3	1 583.7	87.4	10.5
	(4 503.5–7 566.9)	(1 086.4–1 804.9)	(7 521.8–12 527.2)	(1 089.3–1 813.5)	(8 542.1–14 307.6)	(1 203.3–2 016.3)	(81.1–94.0)	(7.6–13.6)
Male	2 812.8	1 356.9	4 690.1	1 363.0	5 231.2	1 479.7	86.0	9.1
	(2 134.4–3 525.4)	(1 033.3–1 703.5)	(3 588.3–5 898.4)	(1 043.0–1 712.2)	(3 973.0–6 631.9)	(1 122.2–1 874.9)	(80.1–92.1)	(6.4–11.8)
Female	3 180.6	1 504.3	5 202.9	1 497.9	6 000.1	1 686.1	88.6	12.1
	(2 366.6–4 059.2)	(1 130.6–1 910.6)	(3 903.0–6 612.1)	(1 121.3–1 904.1)	(4 530.0–7 707.2)	(1 275.3–2 169.2)	(81.6–95.7)	(8.8–15.6)
**Brunei**								
Total	3.83	1 477.6	7.04	1 472.4	7.58	1 553.4	98.1	5.1
	(2.92–4.79)	(1 129.2–1 852.6)	(5.37–8.82)	(1 123.4–1 850.4)	(5.80–9.61)	(1 187.0–1 959.0)	(83.8–115.3)	(-2.1–13.7)
Male	1.97	1 420.4	3.54	1 416.3	3.78	1 473.9	91.6	3.8
	(1.52–2.42)	(1 093.8–1 747.7)	(2.71–4.37)	(1 077.2–1 747.0)	(2.92–4.66)	(1 139.8–1 823.4)	(77.4–106.2)	(-3.5–11.1)
Female	1.85	1 545.6	3.49	1 534.8	3.80	1 643.6	105.1	6.3
	(1.39–2.38)	(1 153.2–1 974.2)	(2.64–4.49)	(1 161.3–1 964.9)	(2.82–4.96)	(1 220.0–2 131.2)	(87.8–127.3)	(-2.5–17.0)
**Cambodia**								
Total	139.1	1 596.0	252.2	1 540.1	292.4	1 717.3	110.2	7.6
	(104.7–177.4)	(1 196.4–2 033.5)	(189.6–323.5)	(1 161.7–1 976.8)	(216.9–382.0)	(1 278.9–2 235.9)	(89.5–135.1)	(-2.6–19.4)
Male	60.4	1 483.3	116.8	1 464.3	133.5	1 604.9	121.0	8.2
	(45.9–76.3)	(1 124.3–1 870.0)	(88.9–148.1)	(1 113.9–1 853.1)	(101.4–172.1)	(1 226.9–2 076.3)	(100.6–144.2)	(-1.0–19.3)
Female	78.7	1 684.2	135.4	1 605.5	158.9	1 818.6	101.9	8.0
	(57.6–101.3)	(1 243.3–2 172.3)	(100.5–175.4)	(1 192.9–2 075.1)	(115.8–211.7)	(1 325.3–2 412.9)	(79.7–129.6)	(-3.6–21.6)
**Indonesia**								
Total	2 400.9	1 385.5	3 973.6	1 392.2	4 581.4	1 555.6	90.8	12.3
	(1 802.4–3 024.8)	(1 044.5–1 744.0)	(3 008.8–5 010.6)	(1 051.0–1 749.3)	(3 448.1–5 838.4)	(1 170.7–1 986.7)	(83.8–98.3)	(9.1–16.1)
Male	1 123.0	1 299.6	1 885.2	1 310.8	2 132.1	1 435.7	89.9	10.5
	(848.1–1 409.5)	(983.4–1 628.4)	(1 435.6–2 368.5)	(998.9–1 647.7)	(1 611.2–2 692.8)	(1 085.7–1 811.4)	(82.6–97.1)	(7.5–13.8)
Female	1 277.8	1 467.6	2 088.4	1 473.7	2 449.2	1 677.1	91.7	14.3
	(954.8–1 628.0)	(1 104.7–1 862.8)	(1 568.6–2 656.7)	(1 104.2–1 873.9)	(1 832.0–3 146.6)	(1 256.7–2 156.2)	(83.9–99.6)	(10.1–18.8)
**Laos**								
Total	57.5	1 596.3	110.5	1 572.3	123.8	1 698.3	115.2	6.4
	(43.2–73.4)	(1 194.9–2 033.9)	(82.4–140.1)	(1 180.1–1 998.4)	(91.8–159.6)	(1 256.5–2 197.2)	(92.9–141.0)	(-4.0–18.7)
Male	27.6	1 567.9	54.9	1 558.7	61.4	1 677.0	122.6	7.0
	(20.9–35.2)	(1 185.8–2 002.6)	(41.8–69.5)	(1 187.9–1 974.3)	(46.1–77.7)	(1 259.8–2 134.8)	(101.6–145.4)	(-3.0–17.8)
Female	30.0	1 621.4	55.5	1 585.6	62.5	1 719.3	108.4	6.0
	(22.0–38.5)	(1 207.6–2 065.2)	(40.7–71.0)	(1 168.8–2 023.3)	(45.3–81.4)	(1 245.1–2 248.9)	(83.1–138.9)	(-6.7–22.3)
**Malaysia**								
Total	267.0	1 659.3	575.6	1 769.4	623.7	1 866.7	133.6	12.5
	(200.0–342.1)	(1 243.4–2 113.3)	(433.4–742.0)	(1 333.9–2 276.1)	(458.9–831.2)	(1 377.2–2 476.7)	(110.3–158.8)	(1.7–24.5)
Male	127.5	1 565.6	281.9	1 675.5	302.1	1 747.8	137.0	11.6
	(95.6–161.9)	(1 172.8–1 997.7)	(214.7–362.2)	(1 277.4–2 145.9)	(222.4–396.2)	(1 282.8–2 293.6)	(114.5–159.7)	(1.5–22.4)
Female	139.5	1 751.9	293.7	1 869.9	321.6	1 994.2	130.4	13.8

	(102.1–181.7)	(1 282.4–2 266.9)	(218.0–384.0)	(1 393.6–2 435.0)	(232.4–431.2)	(1 447.1–2 668.1)	(103.2–160.8)	(0.4–27.9)
**Myanmar**								
Total	520.3	1 370.6	759.8	1 357.1	888.2	1 545.3	70.7	12.7
	(391.0–661.1)	(1 032.4–1 737.4)	(580.4–961.2)	(1 035.5–1 717.6)	(649.4–1 147.5)	(1 130.7–1 996.7)	(54.3–89.2)	(1.8–24.8)
Male	248.7	1 329.2	353.5	1 322.3	405.0	1 473.7	62.8	10.9
	(188.8–313.7)	(1 010.9–1 677.2)	(272.1–443.0)	(1 018.6–1 656.2)	(300.3–518.2)	(1 092.6–1 888.8)	(48.4–77.6)	(0.9–21.4)
Female	271.6	1 408.6	406.3	1 386.3	483.2	1 608.9	77.9	14.2
	(199.7–346.6)	(1 037.0–1 779.3)	(304.3–517.1)	(1 039.2–1 765.5)	(341.3–632.1)	(1 136.7–2 105.3)	(57.2–100.3)	(1.6–28.3)
**Philippines**								
Total	900.8	1 611.5	1 674.8	1 544.2	1 965.0	1 738.8	118.1	7.9
	(679.0–1 143.1)	(1 211.3–2 034.5)	(1 263.4–2 121.3)	(1 161.9–1 953.5)	(1 476.8–2 495.5)	(1 305.4–2 201.4)	(112.1–124.4)	(5.6–10.7)
Male	439.3	1 554.5	810.2	1 476.0	932.4	1 630.5	112.2	4.9
	(334.1–556.7)	(1 178.0–1 962.9)	(614.6–1 022.7)	(1 114.8–1 856.7)	(706.8–1 183.6)	(1 236.1–2 059.8)	(106.5–117.7)	(2.9–7.3)
Female	461.5	1 667.1	864.6	1 611.0	1 032.6	1 846.8	123.7	10.8
	(345.1–588.6)	(1 247.6–2 121.5)	(646.5–1 102.2)	(1 205.3–2 049.1)	(768.3–1 323.8)	(1 381.8–2 358.9)	(117.1–131.1)	(7.9–13.8)
**Singapore**								
Total	57.8	1 733.7	97.6	1 598.0	101.3	1 666.0	75.3	-3.9
	(43.5–73.3)	(1 306.7–2 190.4)	(74.5–121.8)	(1 218.4–1 997.4)	(75.6–129.2)	(1 254.1–2 143.8)	(61.0–89.4)	(-11.7–3.0)
Male	26.7	1 607.5	48.0	1 556.4	49.2	1 611.0	84.6	0.2
	(20.2–33.5)	(1 226.9–2 007.3)	(37.0–59.2)	(1 194.6–1 916.9)	(37.3–61.9)	(1 222.3–2 052.2)	(71.5–97.6)	(-6.4–7.2)
Female	31.1	1 854.9	49.6	1 638.8	52.0	1 721.1	67.3	-7.2
	(23.1–40.0)	(1 379.2–2 370.5)	(37.3–63.4)	(1 234.1–2 103.5)	(38.2–68.0)	(1 263.8–2 248.0)	(50.8–85.2)	(-15.8–1.5)
**Thailand**								
Total	845.3	1 486.9	1 132.6	1 496.2	1 207.0	1 600.7	42.8	7.7
	(642.4–1 072.0)	(1 133.1–1 879.0)	(862.4–1 444.6)	(1 140.0–1 897.0)	(906.5–1 562.7)	(1 207.9–2 082.3)	(29.0–58.3)	(-1.9–18.5)
Male	404.2	1 437.2	524.4	1 455.8	550.7	1 535.2	36.3	6.8
	(309.2–511.0)	(1 097.5–1 812.4)	(402.2–663.2)	(1 123.0–1 836.3)	(416.4–711.8)	(1 172.4–1 984.9)	(23.9–50.2)	(-1.7–16.5)
Female	441.1	1 531.5	608.2	1 530.8	656.2	1 659.5	48.8	8.4
	(329.9–563.3)	(1 151.1–1 958.1)	(457.7–785.1)	(1 142.8–1 956.3)	(480.8–853.2)	(1 228.3–2 170.9)	(32.5–66.8)	(-3.0–21.0)
**Viet Nam**								
Total	800.8	1 296.4	1 309.2	1 268.8	1 441.1	1 369.7	79.9	5.6
	(609.1–1 011.8)	(987.7–1 632.9)	(999.4–1 660.1)	(968.5–1 609.7)	(1 084.7–1 854.3)	(1 031.6–1 763.5)	(64.8–96.9)	(-2.8–14.0)
Male	353.4	1 183.6	611.6	1 198.5	661.0	1 266.6	87.0	7.0
	(272.3–441.6)	(912.1–1 476.1)	(473.7–764.5)	(928.0–1 496.7)	(504.9–841.3)	(967.1–1 609.9)	(73.9–100.8)	(0.1–14.1)
Female	447.4	1 391.5	697.7	1 333.4	780.1	1 468.3	74.4	5.5
	(334.1–570.8)	(1 039.8–1 762.2)	(524.9–897.0)	(1 004.2–1 715.4)	(574.8–1 016.0)	(1 078.9–1 918.9)	(57.3–94.3)	(-4.6–16.7)

95% UI=95% uncertainty interval.

Table S9 | ASEAN percentage of DALYs attributable to mental disorders in 1990, 2019, and 2021, and the percentage change from 1990 to 2021, by country and sex. The 2019 DALYs data serve as a reference for pre-COVID levels. DALYs=disability-adjusted life-years. ASEAN=Association of Southeast Asian Nations.

	Percent DALYs in 1990 (95% UI)	Percent DALYs in 2019 (95% UI)	Percent DALYs in 2021 (95% UI)	Change in percent DALYs from 1990 to 2021 (95% UI)
**ASEAN**				
Total	3.0 (2.4–3.7)	4.9 (4.0–6.0)	4.9 (3.9–6.1)	62.8 (49.1–77.8)
Male	2.6 (2.1–3.2)	4.3 (3.4–5.1)	4.1 (3.3–5.1)	57.6 (41.9–75.8)
Female	3.5 (2.8–4.3)	5.7 (4.7–6.9)	5.9 (4.6–7.4)	69.0 (54.8–84.9)
**Brunei**				
Total	6.5 (5.3–7.8)	7.4 (6.1–8.8)	7.6 (6.1–9.2)	16.2 (4.8–30.3)
Male	5.8 (4.7–7.0)	6.9 (5.7–8.2)	6.9 (5.5–8.3)	18.3 (5.9–33.2)
Female	7.4 (6.1–9.0)	8.0 (6.7–9.6)	8.4 (6.7–10.2)	12.6 (1.5–26.5)
**Cambodia**				
Total	1.8 (1.4–2.2)	4.6 (3.5–5.9)	4.9 (3.6–6.4)	176.1 (128.4–237.9)
Male	1.5 (1.1–1.8)	4.0 (3.0–5.2)	4.2 (3.1–5.6)	190.6 (138.5–257.1)
Female	2.1 (1.6–2.7)	5.1 (3.9–6.5)	5.6 (4.2–7.4)	164.9 (119.7–224.4)
**Indonesia**				
Total	2.8 (2.2–3.5)	4.8 (3.9–5.9)	4.8 (3.6–6.2)	70.9 (46.6–95.9)
Male	2.5 (2.0–3.1)	4.3 (3.2–5.3)	4.1 (3.1–5.3)	62.7 (34.4–95.9)
Female	3.2 (2.5–3.9)	5.5 (4.3–6.7)	5.7 (4.2–7.3)	81.3 (56.8–107.1)
**Laos**				
Total	1.4 (1.0–1.8)	4.5 (3.3–5.8)	4.8 (3.4–6.3)	242.7 (173.9–337.1)
Male	1.3 (0.9–1.6)	4.1 (3.0–5.3)	4.3 (3.0–5.7)	245.2 (175.0–339.9)
Female	1.6 (1.2–2.0)	4.9 (3.6–6.3)	5.3 (3.8–7.0)	242.4 (173.2–329.7)
**Malaysia**				
Total	5.9 (4.8–7.2)	7.2 (5.9–8.8)	6.7 (5.3–8.4)	14.5 (2.9–26.8)
Male	5.1 (4.1–6.2)	6.3 (5.1–7.7)	5.7 (4.4–7.1)	11.7 (1.1–23.6)
Female	6.8 (5.4–8.4)	8.4 (6.8–10.2)	8.1 (6.4–10.1)	18.8 (6.0–33.3)
**Myanmar**				
Total	1.7 (1.3–2.2)	3.7 (2.9–4.7)	4.0 (2.9–5.2)	129.8 (88.2–181.2)
Male	1.5 (1.1–1.9)	3.3 (2.4–4.1)	3.3 (2.4–4.4)	121.3 (81.4–171.4)
Female	2.0 (1.5–2.6)	4.3 (3.3–5.5)	4.7 (3.4–6.3)	136.8 (94.0–188.6)
**Philippines**				
Total	3.7 (2.9–4.6)	5.1 (4.0–6.2)	5.0 (3.9–6.1)	35.5 (23.1–49.2)
Male	3.1 (2.5–3.9)	4.4 (3.5–5.4)	4.2 (3.2–5.2)	32.5 (16.2–49.1)
Female	4.4 (3.5–5.5)	6.0 (4.7–7.4)	6.1 (4.9–7.6)	38.4 (25.0–54.0)
**Singapore**				
Total	8.5 (7.1–10.1)	9.4 (7.9–10.9)	9.2 (7.6–10.8)	8.2 (-1.7–18.1)
Male	7.4 (6.1–8.8)	8.9 (7.5–10.3)	8.6 (7.1–10.1)	16.5 (6.8–26.5)
Female	9.9 (8.1–11.8)	10.0 (8.4–11.8)	10.0 (8.2–11.8)	0.7 (-9.6–11.6)
**Thailand**				
Total	4.8 (3.9–5.9)	5.2 (4.2–6.3)	5.1 (3.9–6.4)	5.7 (-12.2–25.3)
Male	4.1 (3.2–5.0)	4.3 (3.4–5.3)	4.2 (3.1–5.2)	3.0 (-16.0–26.2)
Female	5.9 (4.7–7.2)	6.4 (5.1–7.6)	6.3 (4.8–7.8)	7.2 (-12.3–27.9)
**Viet Nam**				
Total	3.4 (2.6–4.3)	5.0 (4.0–6.1)	5.1 (4.0–6.5)	50.1 (29.8–78.9)

Male	2.7 (2.1–3.4)	4.1 (3.2–5.1)	4.1 (3.2–5.2)	51.8 (30.0–80.8)
Female	4.3 (3.3–5.3)	6.2 (4.9–7.6)	6.5 (5.1–8.1)	50.7 (28.5–80.5)

95% UI=95% uncertainty interval.

Table S10 | Contribution of DALYs (in %) attributable to specific mental disorders to the DALYs attributable to mental disorders in ASEAN, 2021.
DALYs=disability-adjusted life-years. ASEAN=Association of Southeast Asian Nations.

	Total	Female	Male
**Anxiety disorders**	31.0	36.8	24.3
**Attention-deficit hyperactivity disorder**	0.7	0.4	1.0
**Autism spectrum disorders**	7.8	4.9	11.3
**Bipolar disorder**	4.4	4.2	4.7
**Conduct disorder**	3.9	2.7	5.2
**Depressive disorders**	29.6	31.6	27.2
**Eating disorders**	1.9	2.0	1.7
**Idiopathic developmental intellectual disability**	1.6	1.7	1.5
**Other mental disorders**	6.8	5.3	8.5
**Schizophrenia**	12.4	10.5	14.6

Table S11 | Proportion of DALYs (95% UI) (in %) attributable to mental disorders relative to the DALYs for all causes across ASEAN and its member states in 2021, by age group and sex. DALYs=disability-adjusted life-years. ASEAN=Association of Southeast Asian Nations.

	ASEAN	Brunei	Cambodia	Indonesia	Laos	Malaysia	Myanmar	Philippines	Singapore	Thailand	Viet Nam
**All ages**											
Total	4.93	7.56	4.87	4.83	4.77	6.75	3.98	4.99	9.25	5.12	5.13
	(3.87–6.12)	(6.12–9.16)	(3.59–6.41)	(3.63–6.17)	(3.40–6.35)	(5.29–8.37)	(2.89–5.23)	(3.94–6.10)	(7.65–10.85)	(3.85–6.40)	(4.05–6.45)
Male	4.13	6.88	4.23	4.10	4.32	5.72	3.35	4.16	8.59	4.18	4.13
	(3.27–5.11)	(5.53–8.33)	(3.10–5.59)	(3.07–5.31)	(3.02–5.72)	(4.45–7.08)	(2.44–4.38)	(3.19–5.23)	(7.09–10.06)	(3.15–5.25)	(3.24–5.21)
Female	5.94	8.38	5.58	5.73	5.31	8.11	4.74	6.12	9.97	6.31	6.46
	(4.64–7.38)	(6.74–10.21)	(4.19–7.38)	(4.22–7.35)	(3.77–6.99)	(6.36–10.06)	(3.41–6.25)	(4.91–7.62)	(8.17–11.77)	(4.85–7.78)	(5.11–8.11)
**<5 years**											
Total	0.46	1.56	0.36	0.41	0.26	1.34	0.27	0.48	6.15	1.34	0.87
	(0.33–0.64)	(1.04–2.20)	(0.25–0.50)	(0.28–0.57)	(0.17–0.37)	(0.97–1.83)	(0.19–0.39)	(0.34–0.67)	(4.39–8.53)	(1.00–1.80)	(0.58–1.23)
Male	0.50	1.91	0.39	0.41	0.28	1.57	0.29	0.54	8.32	1.63	0.94
	(0.35–0.70)	(1.27–2.71)	(0.26–0.53)	(0.28–0.59)	(0.19–0.41)	(1.12–2.17)	(0.19–0.42)	(0.37–0.75)	(5.94–11.50)	(1.20–2.20)	(0.60–1.36)
Female	0.42	1.10	0.33	0.40	0.23	1.07	0.25	0.42	3.86	0.98	0.77
	(0.30–0.57)	(0.76–1.57)	(0.23–0.45)	(0.27–0.56)	(0.16–0.33)	(0.76–1.51)	(0.17–0.35)	(0.29–0.58)	(2.69–5.34)	(0.70–1.35)	(0.52–1.08)
**5-9 years**											
Total	7.38	16.62	7.07	7.41	6.68	11.42	5.96	6.39	20.48	9.54	8.88
	(5.56–9.22)	(12.79–21.15)	(5.04–9.39)	(5.57–9.33)	(4.67–8.83)	(8.41–15.09)	(4.18–8.08)	(4.78–7.97)	(15.69–25.80)	(7.36–12.04)	(6.71–11.35)
Male	7.08	19.53	6.73	6.91	6.44	11.35	5.42	6.36	23.75	9.91	8.71
	(5.34–8.87)	(15.14–24.38)	(4.71–8.87)	(5.18–8.79)	(4.56–8.34)	(8.32–14.60)	(3.81–7.48)	(4.69–7.95)	(18.51–29.76)	(7.61–12.30)	(6.42–11.17)
Female	7.77	13.50	7.53	8.09	7.00	11.50	6.73	6.44	16.91	9.08	9.13
	(5.79–9.84)	(9.77–18.06)	(5.17–10.27)	(6.01–10.23)	(4.86–9.63)	(8.23–15.89)	(4.54–9.40)	(4.72–8.12)	(12.24–22.42)	(6.81–11.86)	(6.72–11.86)
**10-14 years**											
Total	16.30	25.53	15.69	16.72	14.91	20.73	13.83	15.52	28.16	17.20	16.87
	(12.71–20.54)	(20.23–31.24)	(11.59–20.54)	(13.00–21.37)	(10.94–19.86)	(15.83–26.91)	(10.21–18.02)	(11.88–19.58)	(22.42–34.85)	(13.18–22.17)	(12.81–21.78)
Male	15.53	27.29	14.87	15.77	14.84	19.63	13.10	15.02	31.30	16.77	15.99
	(12.07–19.46)	(22.17–33.06)	(10.93–19.42)	(12.13–19.90)	(10.92–19.48)	(14.92–24.60)	(9.67–16.89)	(11.51–18.73)	(25.50–37.72)	(12.76–21.28)	(11.95–20.43)
Female	17.17	23.65	16.60	17.81	15.00	21.93	14.66	16.09	25.37	17.70	17.93
	(13.23–22.09)	(17.90–29.91)	(12.09–22.29)	(13.63–23.06)	(10.83–20.64)	(16.64–29.17)	(10.65–19.66)	(12.16–20.61)	(19.38–32.55)	(13.01–23.60)	(13.15–23.62)
**15-19 years**											
Total	13.65	24.09	13.41	13.66	13.59	17.77	10.87	14.83	25.33	12.64	13.47
	(10.60–16.96)	(19.66–29.34)	(9.97–17.85)	(10.47–17.24)	(9.58–17.77)	(13.62–22.98)	(7.95–14.34)	(11.82–18.44)	(20.60–30.58)	(9.34–16.45)	(10.16–17.10)
Male	11.75	23.93	11.74	11.94	13.17	14.72	9.19	13.41	26.73	10.01	10.89
	(9.16–14.54)	(19.53–28.80)	(8.61–15.72)	(9.05–15.24)	(9.27–17.45)	(11.16–18.87)	(6.66–12.22)	(10.25–16.78)	(22.12–32.09)	(7.08–13.09)	(8.16–13.60)
Female	16.06	24.26	15.44	15.81	14.03	21.97	13.07	16.50	23.96	16.91	17.10
	(12.36–20.33)	(19.22–29.82)	(10.94–20.51)	(11.77–20.32)	(9.70–18.76)	(16.72–29.02)	(9.43–17.23)	(13.00–20.90)	(19.09–29.89)	(12.53–22.19)	(12.51–21.93)
**20-24 years**											
Total	12.26	19.38	12.34	12.54	12.27	16.23	9.81	12.53	25.13	10.89	12.31
	(9.56–15.37)	(15.73–23.50)	(9.08–16.23)	(9.49–16.05)	(8.52–16.80)	(12.48–21.05)	(7.22–12.75)	(9.83–15.97)	(20.68–30.83)	(7.98–14.08)	(9.35–15.54)
Male	10.19	17.69	10.64	10.69	11.72	13.58	8.24	10.62	23.13	8.14	9.46
	(7.95–12.78)	(14.38–21.42)	(7.82–14.33)	(8.11–13.97)	(8.21–15.83)	(10.33–17.84)	(6.00–10.91)	(7.98–13.74)	(19.13–28.12)	(5.86–10.58)	(6.99–12.06)
Female	14.94	21.26	14.48	14.82	12.86	19.88	11.75	15.01	27.06	15.62	16.55
	(11.55–19.00)	(16.97–26.18)	(10.49–19.06)	(10.81–19.30)	(8.62–17.92)	(15.17–26.13)	(8.49–15.68)	(11.88–19.27)	(21.80–34.06)	(11.49–20.20)	(12.70–21.20)
**25-29 years**											
Total	11.83	17.06	11.75	12.39	11.52	15.45	9.90	11.11	23.01	9.74	12.67

	(9.49–14.69)	(13.96–20.55)	(8.79–15.37)	(9.52–15.50)	(8.48–15.29)	(12.31–19.60)	(7.50–13.00)	(8.96–13.73)	(18.81–27.91)	(7.45–12.42)	(9.96–16.13)
Male	9.95	13.32	10.23	10.81	10.80	13.45	8.53	9.19	20.84	7.19	10.30
	(7.93–12.33)	(10.74–16.00)	(7.62–13.49)	(8.15–13.58)	(7.83–14.42)	(10.70–16.94)	(6.36–11.01)	(7.18–11.53)	(17.11–24.95)	(5.35–9.02)	(7.91–13.06)
Female	14.19	23.01	13.58	14.25	12.34	18.05	11.45	13.72	24.90	13.87	15.93
	(11.37–17.73)	(18.57–27.99)	(10.12–17.99)	(10.99–18.14)	(8.94–16.69)	(14.07–23.36)	(8.64–15.37)	(10.91–17.10)	(20.12–30.65)	(10.56–18.06)	(12.31–20.40)
**30-34 years**											
Total	10.57	14.30	10.37	11.04	10.33	13.29	9.13	9.50	21.87	8.70	11.74
	(8.61–12.84)	(11.62–17.19)	(7.67–13.59)	(8.52–13.69)	(7.66–13.53)	(10.78–16.83)	(6.89–12.00)	(7.68–11.65)	(18.10–26.12)	(6.67–10.90)	(9.26–14.50)
Male	8.84	12.58	8.94	9.59	9.39	11.39	7.69	7.74	21.18	6.42	9.70
	(7.05–10.82)	(10.20–15.03)	(6.48–11.89)	(7.09–12.17)	(6.91–12.49)	(9.04–14.28)	(5.70–10.14)	(6.05–9.65)	(17.49–24.86)	(4.91–8.04)	(7.32–12.05)
Female	12.80	16.51	12.09	12.76	11.46	15.97	10.84	12.04	22.51	12.42	14.52
	(10.37–15.75)	(13.42–20.48)	(9.34–15.47)	(9.93–16.03)	(8.61–15.32)	(12.70–20.35)	(8.23–13.69)	(9.69–14.93)	(18.30–27.56)	(9.51–15.57)	(11.48–17.80)
**35-39 years**											
Total	9.10	10.92	8.98	9.41	9.04	11.38	7.95	8.08	18.38	8.18	10.11
	(7.27–11.14)	(8.85–13.20)	(6.77–11.67)	(7.36–11.80)	(6.73–11.71)	(8.97–14.42)	(5.93–10.39)	(6.49–9.83)	(15.29–21.49)	(6.12–10.29)	(7.79–12.59)
Male	7.55	9.77	7.59	8.16	8.07	9.63	6.56	6.54	17.82	6.05	8.21
	(5.97–9.35)	(7.87–11.86)	(5.56–10.17)	(6.04–10.42)	(5.90–10.68)	(7.55–12.11)	(4.81–8.60)	(5.08–8.22)	(14.68–20.68)	(4.43–7.73)	(6.10–10.32)
Female	11.10	12.45	10.63	10.86	10.24	13.87	9.64	10.31	18.96	11.59	12.73
	(8.95–13.59)	(9.94–15.13)	(8.12–13.45)	(8.35–13.43)	(7.68–13.21)	(10.66–17.52)	(7.32–12.53)	(8.46–12.74)	(15.40–22.51)	(8.84–14.48)	(10.08–15.86)
**40-44 years**											
Total	7.34	9.00	7.18	7.39	7.34	9.18	6.47	6.47	16.45	7.49	8.08
	(5.67–9.12)	(7.27–10.91)	(5.17–9.52)	(5.54–9.51)	(5.44–9.89)	(7.05–11.75)	(4.63–8.56)	(5.07–8.04)	(13.43–19.54)	(5.48–9.57)	(6.23–10.39)
Male	5.97	8.07	5.85	6.30	6.40	7.58	5.19	5.14	16.46	5.61	6.28
	(4.62–7.50)	(6.55–9.74)	(4.11–8.04)	(4.55–8.22)	(4.62–8.74)	(5.80–9.68)	(3.63–6.89)	(3.91–6.55)	(13.46–19.35)	(4.01–7.23)	(4.69–8.06)
Female	9.14	10.21	8.75	8.70	8.54	11.51	8.08	8.44	16.44	10.43	10.72
	(7.08–11.41)	(7.99–12.53)	(6.35–11.41)	(6.40–11.11)	(6.36–11.43)	(8.69–14.61)	(5.94–10.66)	(6.71–10.53)	(12.91–20.09)	(7.92–13.08)	(8.13–13.71)
**45-49 years**											
Total	5.84	6.45	5.64	5.74	5.90	7.32	5.17	5.17	11.64	6.73	6.28
	(4.53–7.35)	(5.07–7.91)	(4.03–7.55)	(4.20–7.42)	(4.26–8.03)	(5.47–9.28)	(3.70–6.85)	(4.03–6.52)	(9.51–13.81)	(4.95–8.49)	(4.70–8.11)
Male	4.69	5.46	4.51	4.84	5.10	5.92	4.09	4.06	11.02	5.14	4.67
	(3.60–5.93)	(4.33–6.66)	(3.19–6.15)	(3.45–6.35)	(3.54–7.09)	(4.44–7.51)	(2.85–5.47)	(3.07–5.23)	(9.02–12.97)	(3.68–6.61)	(3.45–6.04)
Female	7.39	7.78	6.98	6.85	6.92	9.32	6.55	6.88	12.37	9.09	8.85
	(5.76–9.32)	(6.09–9.67)	(4.92–9.06)	(4.90–9.01)	(5.12–9.35)	(6.96–11.98)	(4.82–8.64)	(5.33–8.68)	(10.00–15.02)	(6.69–11.46)	(6.67–11.36)
**50-54 years**											
Total	4.51	4.72	4.28	4.26	4.48	5.67	3.93	4.08	9.36	5.83	4.76
	(3.47–5.70)	(3.65–5.89)	(3.05–5.86)	(3.12–5.59)	(3.15–6.12)	(4.21–7.18)	(2.78–5.20)	(3.11–5.13)	(7.48–11.19)	(4.16–7.40)	(3.52–6.27)
Male	3.54	3.99	3.30	3.52	3.86	4.52	3.02	3.13	8.75	4.46	3.37
	(2.69–4.50)	(3.07–4.91)	(2.35–4.65)	(2.48–4.68)	(2.65–5.23)	(3.35–5.72)	(2.12–4.16)	(2.32–4.07)	(7.14–10.41)	(3.15–5.82)	(2.49–4.43)
Female	5.86	5.65	5.43	5.22	5.26	7.32	5.10	5.60	10.10	7.81	7.13
	(4.47–7.44)	(4.34–7.07)	(3.91–7.33)	(3.72–6.93)	(3.79–7.17)	(5.42–9.36)	(3.78–6.76)	(4.35–7.15)	(7.92–12.56)	(5.70–9.83)	(5.29–9.35)
**55-59 years**											
Total	3.49	3.83	3.24	3.17	3.36	4.33	2.98	3.19	7.07	5.01	3.70
	(2.65–4.51)	(2.93–4.80)	(2.31–4.48)	(2.25–4.23)	(2.30–4.55)	(3.19–5.51)	(2.07–4.07)	(2.40–4.02)	(5.60–8.57)	(3.57–6.46)	(2.70–4.93)
Male	2.68	3.24	2.45	2.56	2.87	3.40	2.25	2.39	6.15	3.87	2.51
	(2.00–3.49)	(2.51–4.01)	(1.73–3.44)	(1.78–3.50)	(1.93–3.97)	(2.48–4.40)	(1.57–3.13)	(1.76–3.15)	(4.92–7.34)	(2.61–5.14)	(1.84–3.39)
Female	4.64	4.58	4.28	4.00	4.01	5.70	3.95	4.47	8.28	6.59	5.69
	(3.46–5.97)	(3.53–5.92)	(3.06–5.80)	(2.77–5.46)	(2.83–5.39)	(4.16–7.34)	(2.78–5.36)	(3.40–5.70)	(6.53–10.27)	(4.87–8.47)	(4.13–7.49)
**60-64 years**											

Total	2.60	2.74	2.47	2.28	2.47	3.29	2.19	2.38	5.14	3.96	2.76
	(1.92–3.41)	(2.08–3.41)	(1.72–3.42)	(1.59–3.06)	(1.66–3.44)	(2.36–4.25)	(1.52–3.04)	(1.78–3.06)	(4.01–6.29)	(2.80–5.22)	(1.96–3.71)
Male	1.98	2.25	1.82	1.82	2.12	2.57	1.66	1.75	4.39	3.13	1.84
	(1.46–2.63)	(1.75–2.78)	(1.27–2.55)	(1.24–2.53)	(1.45–2.92)	(1.83–3.39)	(1.15–2.32)	(1.27–2.30)	(3.50–5.29)	(2.15–4.16)	(1.35–2.46)
Female	3.47	3.37	3.18	2.93	2.95	4.31	2.86	3.38	6.18	5.04	4.22
	(2.51–4.59)	(2.53–4.39)	(2.22–4.43)	(1.99–4.07)	(2.01–4.09)	(3.14–5.66)	(2.00–3.97)	(2.54–4.31)	(4.72–7.86)	(3.63–6.50)	(3.00–5.65)
**65-69 years**											
Total	1.89	2.14	1.82	1.62	1.79	2.47	1.57	1.77	4.04	2.98	1.95
	(1.39–2.49)	(1.66–2.70)	(1.29–2.54)	(1.15–2.17)	(1.21–2.48)	(1.80–3.21)	(1.09–2.15)	(1.32–2.29)	(3.20–4.95)	(2.15–3.96)	(1.42–2.63)
Male	1.43	1.87	1.34	1.28	1.54	1.92	1.19	1.29	3.43	2.39	1.30
	(1.07–1.90)	(1.45–2.34)	(0.93–1.90)	(0.90–1.77)	(1.04–2.15)	(1.38–2.51)	(0.83–1.61)	(0.94–1.67)	(2.76–4.16)	(1.68–3.24)	(0.96–1.72)
Female	2.49	2.43	2.28	2.08	2.12	3.25	2.01	2.49	4.84	3.70	2.88
	(1.79–3.25)	(1.87–3.12)	(1.60–3.17)	(1.42–2.89)	(1.45–2.91)	(2.38–4.28)	(1.39–2.83)	(1.84–3.19)	(3.73–6.12)	(2.65–4.90)	(2.07–3.92)
**70-74 years**											
Total	1.39	1.58	1.30	1.16	1.32	1.84	1.13	1.36	3.07	2.24	1.37
	(1.05–1.82)	(1.24–2.01)	(0.93–1.79)	(0.84–1.55)	(0.91–1.82)	(1.37–2.39)	(0.80–1.53)	(1.02–1.74)	(2.41–3.77)	(1.61–2.94)	(1.02–1.80)
Male	1.08	1.40	0.98	0.93	1.15	1.46	0.88	0.98	2.53	1.82	0.93
	(0.81–1.39)	(1.10–1.77)	(0.71–1.36)	(0.67–1.28)	(0.79–1.60)	(1.10–1.92)	(0.62–1.19)	(0.72–1.27)	(2.01–3.08)	(1.30–2.42)	(0.71–1.22)
Female	1.78	1.77	1.57	1.45	1.52	2.34	1.39	1.85	3.79	2.72	1.93
	(1.30–2.30)	(1.33–2.28)	(1.13–2.20)	(1.01–1.97)	(1.06–2.08)	(1.73–3.07)	(0.97–1.95)	(1.39–2.39)	(2.93–4.82)	(1.94–3.56)	(1.41–2.64)
**75-79 years**											
Total	1.06	0.98	0.96	0.86	1.00	1.33	0.85	1.07	2.28	1.70	0.99
	(0.80–1.35)	(0.76–1.25)	(0.70–1.32)	(0.63–1.16)	(0.71–1.37)	(0.97–1.76)	(0.59–1.15)	(0.79–1.37)	(1.77–2.86)	(1.24–2.25)	(0.74–1.30)
Male	0.83	0.85	0.75	0.70	0.90	1.09	0.67	0.76	1.83	1.39	0.68
	(0.62–1.08)	(0.65–1.10)	(0.55–1.01)	(0.51–0.95)	(0.63–1.21)	(0.79–1.44)	(0.48–0.91)	(0.56–1.01)	(1.44–2.27)	(0.98–1.87)	(0.51–0.87)
Female	1.29	1.13	1.13	1.03	1.12	1.61	1.01	1.37	2.76	2.02	1.31
	(0.96–1.66)	(0.85–1.50)	(0.80–1.57)	(0.75–1.39)	(0.79–1.55)	(1.17–2.19)	(0.70–1.42)	(1.02–1.77)	(2.09–3.50)	(1.43–2.66)	(0.96–1.76)
**80-84 years**											
Total	0.82	0.74	0.73	0.65	0.78	1.03	0.64	0.84	1.65	1.30	0.74
	(0.63–1.07)	(0.56–0.96)	(0.54–1.01)	(0.48–0.88)	(0.55–1.06)	(0.74–1.37)	(0.45–0.89)	(0.62–1.09)	(1.27–2.09)	(0.94–1.74)	(0.55–0.99)
Male	0.66	0.64	0.58	0.53	0.70	0.92	0.52	0.62	1.26	1.07	0.51
	(0.49–0.85)	(0.49–0.82)	(0.43–0.80)	(0.39–0.73)	(0.48–0.96)	(0.66–1.24)	(0.36–0.71)	(0.45–0.81)	(0.97–1.59)	(0.75–1.45)	(0.38–0.66)
Female	0.96	0.84	0.83	0.75	0.85	1.13	0.74	1.00	2.02	1.49	0.91
	(0.73–1.26)	(0.62–1.10)	(0.59–1.14)	(0.55–1.01)	(0.59–1.17)	(0.81–1.50)	(0.52–1.04)	(0.73–1.30)	(1.51–2.62)	(1.07–1.99)	(0.67–1.23)
**85-89 years**											
Total	0.68	0.58	0.57	0.50	0.62	0.84	0.51	0.68	1.30	1.13	0.59
	(0.52–0.88)	(0.43–0.75)	(0.41–0.77)	(0.37–0.67)	(0.44–0.87)	(0.61–1.11)	(0.35–0.71)	(0.51–0.88)	(0.98–1.66)	(0.82–1.52)	(0.44–0.78)
Male	0.56	0.35	0.45	0.40	0.54	0.83	0.41	0.61	0.99	0.94	0.40
	(0.42–0.73)	(0.26–0.45)	(0.32–0.60)	(0.30–0.55)	(0.39–0.76)	(0.60–1.11)	(0.28–0.57)	(0.46–0.79)	(0.76–1.22)	(0.68–1.26)	(0.30–0.52)
Female	0.77	0.76	0.65	0.58	0.69	0.85	0.59	0.72	1.54	1.26	0.68
	(0.58–0.99)	(0.57–1.00)	(0.47–0.89)	(0.42–0.78)	(0.48–0.96)	(0.60–1.14)	(0.40–0.82)	(0.52–0.93)	(1.14–2.01)	(0.89–1.72)	(0.50–0.92)
**90-94 years**											
Total	0.53	0.47	0.43	0.37	0.47	0.61	0.38	0.45	0.94	0.81	0.46
	(0.38–0.69)	(0.34–0.60)	(0.30–0.60)	(0.27–0.50)	(0.33–0.64)	(0.43–0.83)	(0.26–0.54)	(0.34–0.58)	(0.69–1.26)	(0.57–1.11)	(0.33–0.62)
Male	0.45	0.21	0.33	0.29	0.40	0.60	0.30	0.46	0.69	0.69	0.30
	(0.33–0.59)	(0.15–0.27)	(0.22–0.44)	(0.21–0.40)	(0.27–0.56)	(0.42–0.81)	(0.20–0.42)	(0.34–0.59)	(0.52–0.89)	(0.49–0.93)	(0.22–0.39)
Female	0.58	0.51	0.49	0.43	0.51	0.61	0.43	0.45	1.09	0.89	0.52
	(0.41–0.76)	(0.37–0.66)	(0.33–0.67)	(0.30–0.57)	(0.35–0.70)	(0.42–0.83)	(0.29–0.61)	(0.33–0.58)	(0.78–1.48)	(0.62–1.23)	(0.37–0.70)

**95+ years**											
Total	0.43	0.20	0.33	0.27	0.34	0.42	0.28	0.32	0.66	0.62	0.37
	(0.30–0.58)	(0.14–0.28)	(0.22–0.47)	(0.19–0.37)	(0.23–0.48)	(0.28–0.60)	(0.19–0.39)	(0.23–0.43)	(0.45–0.91)	(0.41–0.88)	(0.26–0.53)
Male	0.39	0.12	0.24	0.21	0.29	0.48	0.22	0.35	0.48	0.55	0.22
	(0.27–0.52)	(0.09–0.16)	(0.16–0.35)	(0.15–0.30)	(0.19–0.41)	(0.32–0.69)	(0.14–0.31)	(0.25–0.46)	(0.34–0.66)	(0.37–0.80)	(0.15–0.29)
Female	0.45	0.23	0.36	0.30	0.37	0.35	0.30	0.29	0.74	0.66	0.41
	(0.31–0.61)	(0.16–0.31)	(0.23–0.51)	(0.20–0.41)	(0.25–0.52)	(0.23–0.49)	(0.20–0.42)	(0.21–0.38)	(0.49–1.03)	(0.43–0.95)	(0.28–0.57)

95% UI=95% uncertainty interval.

GATHER checklist

Item #	Checklist item	Reporting location
**Objectives and funding**		
1	Define the indicator(s), populations (including age, sex, and geographic entities), and time period(s) for which estimates were made.	Main text – Methods, Overview and Case definitions subsections
2	List the funding sources for the work.	Main text – Acknowledgement section
**Data Inputs**		
For all data inputs from multiple sources that are synthesized as part of the study:		
3	Describe how the data were identified and how the data were accessed.	Main text – Methods, Overview, p. 8;<br>Appendix – Supplemental Methods, Figure S1 and Table S1
4	Specify the inclusion and exclusion criteria. Identify all ad-hoc exclusions.	Main text – Methods, Overview and Case definitions subsections, appendix supplemental methods p. 14
5	Provide information on all included data sources and their main characteristics. For each data source used, report reference information or contact name/institution, population represented, data collection method, year(s) of data collection, sex and age range, diagnostic criteria or measurement method, and sample size, as relevant.	Appendix – Supplemental Methods, Figure S1 and Table S1
6	Identify and describe any categories of input data that have potentially important biases (e.g., based on characteristics listed in item 5).	Main text – Limitations subsection<br>Appendix – Supplemental Methods, Table S1
For data inputs that contribute to the analysis but were not synthesized as part of the study:		
7	Describe and give sources for any other data inputs.	N/A
For all data inputs:		
8	Provide all data inputs in a file format from which data can be efficiently extracted (e.g., a spreadsheet rather than a PDF), including all relevant meta-data listed in item 5. For any data inputs that cannot be shared because of ethical or legal reasons, such as third-party ownership, provide a contact name or the name of the institution that retains the right to the data.	Data inputs in a spreadsheet format are available on the GHDx at https://ghdx.healthdata.org/gbd-2021/sources
**Data analysis**		
9	Provide a conceptual overview of the data analysis method. A diagram may be helpful.	Main text – Methods, Overview and Case definitions subsections;<br>Appendix – Supplemental Methods, Model flow charts
10	Provide a detailed description of all steps of the analysis, including mathematical formulae. This description should cover, as relevant, data cleaning, data pre-processing, data adjustments and weighting of data sources, and mathematical or statistical model(s).	Appendix – Supplemental Methods, Model flow charts
11	Describe how candidate models were evaluated and how the final model(s) were selected.	Appendix – Supplemental Methods, Model flow charts
12	Provide the results of an evaluation of model performance, if done, as well as the results of any relevant sensitivity analysis.	N/A
13	Describe methods for calculating uncertainty of the estimates. State which sources of uncertainty were, and were not, accounted for in the uncertainty analysis.	Main text – Methods, Case definitions, Disability-adjusted life years (DALYs) subsection
14	State how analytic or statistical source code used to generate estimates can be accessed.	The source code can be found on the GHDx at https://ghdx.healthdata.org/gbd-2021/code
**Results and Discussion**		
15	Provide published estimates in a file format from which data can be efficiently extracted.	The results can be efficiently extracted at https://vizhub.healthdata.org/gbd-results/
16	Report a quantitative measure of the uncertainty of the estimates (e.g. uncertainty intervals).	95% Uncertainty Intervals (UIs) given for all findings, including in the text, figures, and tables in the main text and Supplemental Appendix; online viz tools (see information above)
17	Interpret results in light of existing evidence. If updating a previous set of estimates, describe the reasons for changes in estimates.	Main text – Discussion section, 2nd-5th paragraphs
18	Discuss limitations of the estimates. Include a discussion of any modelling assumptions or data limitations that affect interpretation of the estimates.	Main text – Discussion, Limitations subsection

References

1 Major depressive disorder | Institute for Health Metrics and Evaluation. https://www.healthdata.org/gbd/methods-appendices-2021/major-depressive-disorder (accessed Feb 7, 2025).

2 Anxiety disorders | Institute for Health Metrics and Evaluation. https://www.healthdata.org/gbd/methods-appendices-2021/anxiety-disorders (accessed Feb 7, 2025).
