# Supplementary material for: The epidemiology and burden of ten mental disorders in countries of the Association of Southeast Asian Nations (ASEAN), 1990–2021: findings from the Global Burden of Disease Study 2021
Source: Lancet Public Health. 2025 May 27;10(6):e480–91. doi: 10.1016/S2468-2667(25)00098-2 (PMC12127263; doi:10.1016/S2468-2667(25)00098-2)
Supplement: Supplementary appendix [file mmc2.pdf]

THE LANCET
Public Health

Supplementary appendix 2

This appendix formed part of the original submission and has been peer reviewed. We post it as supplied by the authors.

Supplement to: GBD 2021 ASEAN Mental Disorders Collaborators. The epidemiology and burden of ten mental disorders in countries of the Association of Southeast Asian Nations (ASEAN), 1990–2021: findings from the Global Burden of Disease Study 2021. *Lancet Public Health* 2025; **10:** e480–91.

Appendix 2

Authors' affiliations

Division of Family Medicine (A Szücs MD, S C C van der Lubbe PhD), Centre for Research in Health Systems Performance (Prof J Valderas PhD), Department of Surgery (J Lau MPH), Department of Psychological Medicine (Prof A Rodriguez PhD), Yong Loo Lin School of Medicine (M Ng PhD), National University of Singapore, Singapore, Singapore; Care in Long Term Conditions Division (J Arias de la Torre PhD), King's College London, London, UK; CIBER Epidemiology and Public Health (CIBERESP), Madrid, Spain (J Arias de la Torre PhD); Institute for Health Metrics and Evaluation (Prof S I Hay FMedSci, C Bisignano MPH, B W Morgan MSPH, Prof C J L Murray DPhil, D F Santomauro PhD, M Ng PhD), Department of Health Metrics Sciences, School of Medicine (Prof S I Hay FMedSci, Prof C J L Murray DPhil), University of Washington, Seattle, WA, USA; Department of Oral Pathology and Microbiology (S Acharya MDS), JSS Academy of Higher Education and Research, Mysuru, India; Department of Public Health (Q Adnani PhD), Universitas Padjadjaran (Padjadjaran University), Bandung, Indonesia; School of Medicine and Public Health (G C Apostol MD), School of Medicine and Public Health, Center for Research and Innovation (L C Sumpaico-Tanchanco MD), Ateneo De Manila University, Pasig City, Philippines; Inter-Agency Committee on Environmental Health (G C Apostol MD), Health Technology Assessment Unit (Y H Zuniga BS), Department of Health Philippines, Manila, Philippines; School of Traditional Chinese Medicine (M Aslam PhD, Y Kim PhD), Xiamen University Malaysia, Sepang, Malaysia; Nursing Department (Y Asri PhD), Faculty of Health Science (Y Asri PhD), Institute of Technology and Health Science RS dr Soepraoen, Malang, Indonesia; Burden of Disease Research Program (Z Aung MPH), International Health Policy Program, Nonthaburi, Thailand; Faculty of Nursing (G E Aurizki MSc), Advanced Nursing Department (F Efendi PhD), Universitas Airlangga (Airlangga University), Surabaya, Indonesia; Division of Nursing, Midwifery and Social Work, School of Health Sciences (G E Aurizki MSc), University of Manchester, Manchester, UK; International Medical School (A A Baig PhD), Management and Science University, Alam, Malaysia; Department of Epidemiology and Biostatistics (A C Bermudez MD), Department of Environmental and Occupational Health (C M Estrada PhD), Department of Health Policy and Administration (Prof F B Garcia PhD), Department of Neurosciences (Prof R G Jamora PhD), National Institutes of Health (A Loreche MS), University of the Philippines Manila, Manila, Philippines; Department of Epidemiology (A C Bermudez MD), Brown University, Providence, RI, USA; College of Public Health, Medical, and Veterinary Sciences (M Cenderadewi MPHTM), James Cook University, Townsville, QLD, Australia; Department of Public Health (M Cenderadewi MPHTM), University of Mataram, Mataram, Indonesia; Department of Internal Medicine (P Danpanichkul MD), Texas Tech

University, Lubbock, TX, USA; Centre for Public Health, Equity and Human Flourishing (N K Fauk PhD), Torrens University Australia, Adelaide, SA, Australia; Institute of Resource Governance and Social Change, Kupang, Indonesia (N K Fauk PhD); Graduate Institute of Injury Prevention and Control (N Fridayani MSc), School of Nursing (M Kurniasari PhD, A L Wicaksana MS), Taipei Medical University, Taipei, Taiwan; Faculty of Nursing (F Hasan PhD), Center of Excellence in Genomics and Precision Dentistry (T Porntaveetus PhD), Chulalongkorn University, Bangkok, Thailand; Faculty of Pharmacy (U I Ibrahim PhD), Sultan Zainal Abidin University, Malaysia, Terengganu, Malaysia; Department of Neurosurgery, National Brain Center Hospital Prof. Dr. dr. Mahar Mardjono (M F Ilyas MD), Universitas Sebelas Maret, Jakarta, Indonesia; Department of Clinical Pharmacy & Pharmacy Practice (Prof N Ismail PhD), Asian Institute of Medicine, Science and Technology, Bedong, Malaysia; Malaysian Academy of Pharmacy, Puchong, Malaysia (Prof N Ismail PhD); Department of Primary Care Medicine (J Jamaluddin MMed), Faculty of Pharmacy (K Khan PhD), University of Malaya, Kuala Lumpur, Malaysia; Institute for Neurosciences (Prof R G Jamora PhD), St. Luke's Medical Center, Bonifacio Global City, Philippines; Rothschild Foundation Hospital (Prof J B Jonas MD), Institut Français de Myopie, Paris, France; Singapore Eye Research Institute (Prof J B Jonas MD), Singapore Eye Research Institute, Singapore, Singapore; Faculty of Dentistry (Prof K K Kanmodi DDS, A A Salami BDS), University of Puthisastra, Phnom Penh, Cambodia; Office of the Executive Director (Prof K K Kanmodi DDS), Cephas Health Research Initiative Inc, Ibadan, Nigeria; Poltekkes Kemenkes Semarang (F H Khotimah Bdn), Ministry of Health of the Republic of Indonesia, Semarang, Indonesia; Faculty of Medicine and Health Science (M Kurniasari PhD), Universitas Kristen Satya Wacana, Salatiga, Indonesia; Institute for Health Sciences (C Y Kustanti PhD), STIKES Bethesda Yakkum Yogyakarta Indonesia, Yogyakarta, Indonesia; Department of Public Health and Epidemiology (D Kusuma DSc), Khalifa University of Science and Technology, Abu Dhabi, United Arab Emirates; Faculty of Public Health (D Kusuma DSc), University of Indonesia, Depok, Indonesia; Department of Physiotherapy (T Laksono MS), Universitas Aisyiyah Yogyakarta, Yogyakarta, Indonesia; Institute of Allied Health Sciences (T Laksono MS), National Cheng Kung University, Tainan, Taiwan; Ateneo School of Government (A Loreche MS), Ateneo De Manila University, Quezon City, Philippines; Centre for Public Health and Wellbeing (Z Ma PhD), University of the West of England, Bristol, UK; School of Public Health (J C Maravilla PhD, D F Santomauro PhD), The University of Queensland, Brisbane, QLD, Australia; Far Eastern University, Manila, Philippines (J C Maravilla PhD); Faculty of Humanities and Health Sciences (Prof R R Marzo MD), Curtin University, Sarawak, Malaysia; Jeffrey Cheah School of Medicine and Health Sciences (Prof R R Marzo MD), School of Pharmacy (Y Wong PhD), Monash University, Subang Jaya, Malaysia; Department of Epidemiology (Prof R J Maude PhD), Mahidol-Oxford Tropical Medicine Research Unit, Bangkok, Thailand; Nuffield Department of Medicine (Prof R J Maude PhD), University of Oxford, Oxford, UK; Department of Nutrition Science (S Rahmawaty PhD), Muhammadiyah University of

Surakarta, Surakarta, Indonesia; School of Allied Health (Prof A Rodriguez PhD), Anglia Ruskin University, Chelmsford, UK; Faculty of Medicine (B Roy PhD), Quest International University Perak, Ipoh, Malaysia; Department of Oral and Maxillofacial Surgery (A A Salami BDS), University College Hospital, Ibadan, Ibadan, Nigeria; Institute of Epidemiology and Preventive Medicine (Y L Samodra PhD), National Taiwan University, Taipei, Taiwan; Benang Merah Research Center (BMRC), Minahasa Utara, Indonesia (Y L Samodra PhD); Department of Public Health and Community Medicine (Prof C T Sreeramareddy MD), International Medical University, Kuala Lumpur, Malaysia; Medical and Diagnostic Research Centre (Prof C T Sreeramareddy MD), University of Hail, Hail, Saudi Arabia; Department of Medical Sciences (Prof V Subramaniyan PhD), Sunway University, Subang Jaya, Malaysia; Praboromarajchanok Institute (T Sukaew PhD), Ministry of Public Health, Nonthaburi, Thailand; Faculty of Health Science (D Sulistiyorini MSc), Universitas Indonesia Maju, Jakarta, Indonesia; Department of Medical Surgical Nursing (A L Wicaksana MS), Gadjah Mada University, Yogyakarta, Indonesia; Department of Health Policy and Management (Prof M Z Younis PhD), Jackson State University, Jackson, MS, USA; School of Business & Economics (Prof M Z Younis PhD), Universiti Putra Malaysia (University of Putra Malaysia), Kuala Lumpur, Malaysia; #MentalHealthPH, Inc., Quezon City, Philippines (Y H Zuniga BS); Queensland Centre for Mental Health Research, Wacol, QLD, Australia (D F Santomauro PhD)

Authors' contributions

Providing data or critical feedback on data sources

Swetha Acharya, Qorinah Estiningtyas Sakilah Adnani, Geminn Louis Carace Apostol, Muhammad Shahzad Aslam, Yuni Asri, Zaw Zaw Aung, Gading Ekapuja Aurizki, Atif Amin Baig, Pojsakorn Danpanichkul, Ni Kadek Yuni Fridayani, Fernando Barroga Garcia, Faizul Hasan, Muhana Fawwazy Ilyas, Nahlah Elkudssiah Ismail, Jost B Jonas, Fireza Husnul Khotimah, Yun Jin Kim, Maria Dyah Kurniasari, Zheng Feei Ma, Joemer C Maravilla, Roy Rillera Marzo, Brooks W Morgan, Christopher J L Murray, Thantrira Porntaveetus, Afeez Abolarinwa Salami, Damian F Santomauro, Chandrashekhar T Sreeramareddy, Vetriselvan Subramaniyan, Thitiporn Sukaew, Yen Jun Wong, Mustafa Z Younis, and Yves Miel H Zuniga.

Developing methods or computational machinery

Simon I Hay, Christopher J L Murray, and Damian F Santomauro.

Providing critical feedback on methods or results

Swetha Acharya, Qorinah Estiningtyas Sakilah Adnani, Geminn Louis Carace Apostol, Jorge Arias de la Torre, Muhammad Shahzad Aslam, Yuni Asri, Zaw Zaw Aung, Gading Ekapuja Aurizki, Atif Amin Baig, Amiel Nazer C Bermudez, Muthia Cenderadewi, Pojsakorn Danpanichkul, Ferry Efendi, Crystal Amiel M Estrada, Ni Kadek Yuni Fridayani,

Fernando Barroga Garcia, Faizul Hasan, Simon I Hay, Umar Idris Ibrahim, Muhana Fawwazy Ilyas, Nahlah Elkudssiah Ismail, Jazlan Jamaluddin, Roland Dominic G Jamora, Jost B Jonas, Kehinde Kazeem Kanmodi, Kashif Ullah Khan, Yun Jin Kim, Maria Dyah Kurniasari, Christina Yeni Kustanti, Dian Kusuma, Tri Laksono, Jerrald Lau, Arianna Maever Loreche, Zheng Feei Ma, Joemer C Maravilla, Roy Rillera Marzo, Richard James Maude, Christopher J L Murray, Thantrira Porntaveetus, Setyaningrum Rahmawaty, Alina Rodriguez, Afeez Abolarinwa Salami, Yoseph Leonardo Samodra, Damian F Santomauro, Chandrashekhar T Sreeramareddy, Vetriselvan Subramaniyan, Thitiporn Sukaew, Desy Sulistiyorini, Lourdes Bernadette C Sumpaico-Tanchanco, Jose M Valderas, Stephanie C C van der Lubbe, Anggi Lukman Wicaksana, Yen Jun Wong, Mustafa Z Younis, and Yves Miel H Zuniga.

Drafting the work or revising it critically for important intellectual content

Swetha Acharya, Qorinah Estiningtyas Sakilah Adnani, Geminn Louis Carace Apostol, Jorge Arias de la Torre, Muhammad Shahzad Aslam, Yuni Asri, Gading Ekapuja Aurizki, Atif Amin Baig, Catherine Bisignano, Muthia Cenderadewi, Pojsakorn Danpanichkul, Ferry Efendi, Nelsensius Klau Fauk, Faizul Hasan, Simon I Hay, Umar Idris Ibrahim, Muhana Fawwazy Ilyas, Nahlah Elkudssiah Ismail, Jazlan Jamaluddin, Jost B Jonas, Kehinde Kazeem Kanmodi, Yun Jin Kim, Maria Dyah Kurniasari, Christina Yeni Kustanti, Dian Kusuma, Zheng Feei Ma, Roy Rillera Marzo, Brooks W Morgan, Christopher J L Murray, Marie Ng, Thantrira Porntaveetus, Alina Rodriguez, Bedanta Roy, Afeez Abolarinwa Salami, Damian F Santomauro, Chandrashekhar T Sreeramareddy, Thitiporn Sukaew, Anna Szücs, Jose M Valderas, Stephanie C C van der Lubbe, and Anggi Lukman Wicaksana.

Managing the estimation or publications process

Simon I Hay, Christopher J L Murray, Marie Ng, and Damian F Santomauro.
